# Peridermal fruit skin formation in *Actinidia* sp. (kiwifruit) is associated with genetic *loci* controlling russeting and cuticle formation

**DOI:** 10.1186/s12870-021-03025-2

**Published:** 2021-07-14

**Authors:** Nikolai Macnee, Elena Hilario, Jibran Tahir, Alastair Currie, Ben Warren, Ria Rebstock, Ian C. Hallett, David Chagné, Robert J. Schaffer, Sean M. Bulley

**Affiliations:** 1 The New Zealand Institute for Plant and Food Research Ltd. (PFR), Private Bag 92169, Auckland, 1142 New Zealand; 2 School of Biological Science, The University of Auckland, Auckland, 1146 New Zealand; 3 PFR, Private Bag 11600, Palmerston North, 4442 New Zealand; 4 PFR, 55 Old Mill Road, RD3, Motueka, 7198 New Zealand; 5 PFR, 412 No 1 Road RD 2, Te Puke, 3182 New Zealand

**Keywords:** Kiwifruit, *Actinidia* sp., Pericarp, Periderm, Russet, Cuticle

## Abstract

**Background:**

The skin (exocarp) of fleshy fruit is hugely diverse across species. Most fruit types have a live epidermal skin covered by a layer of cuticle made up of cutin while a few create an outermost layer of dead cells (peridermal layer).

**Results:**

In this study we undertook crosses between epidermal and peridermal skinned kiwifruit, and showed that epidermal skin is a semi-dominant trait. Furthermore, backcrossing these epidermal skinned hybrids to a peridermal skinned fruit created a diverse range of phenotypes ranging from epidermal skinned fruit, through fruit with varying degrees of patches of periderm (russeting), to fruit with a complete periderm. Quantitative trait locus (QTL) analysis of this population suggested that periderm formation was associated with four loci. These QTLs were aligned either to ones associated with russet formation on chromosome 19 and 24, or cuticle integrity and coverage located on chromosomes 3, 11 and 24.

**Conclusion:**

From the segregation of skin type and QTL analysis, it appears that skin development in kiwifruit is controlled by two competing factors, cuticle strength and propensity to russet. A strong cuticle will inhibit russeting while a strong propensity to russet can create a continuous dead skinned periderm.

**Supplementary Information:**

The online version contains supplementary material available at 10.1186/s12870-021-03025-2.

## Background

In plants, the exocarp (skin) of fleshy fruits interfaces with the environment, is essential for protection and support of the internal organs, and often changes to attract herbivores for seed dispersal. The morphology of exocarp types is diverse between fruit species, ranging in color, composition and structure. While most fruits have live epidermal skin, covered in a thick waxy cuticle, some fruit form a peridermal skin (periderm) which forms a dead layer to protect the fruit. The periderm is formed by a multistage process that involves the development of a cork cambium meristem just under the epidermis. The cork cambium meristem creates a number of thin-walled cell layers that are sequentially suberized or lignified and undergo programmed cell death [1]. Periderm formation can either occur over the whole fruit, or only on parts of the fruit forming russeting disorders.

There are a number of fruits that have closely related species that show both epidermal and peridermal skin types including *Actinidia* sp. (kiwifruit), *Pyrus* sp. (pear) and *Malus* sp. (apple). Studies in these species have shown periderm formation (russeting) is controlled by both genetics and environment. The skin of a Japanese pear (*Pyrus pyrifolia* ‘Nakai’) can be russeted with a characteristic peridermal layer, and is preferred by consumers [2]. Early test cross studies [2] predicted russet formation to be a dominant trait with two controlling factors [3]. Later studies using bulked segregant analysis of two Japanese pear segregating populations identified a RAPD marker that explained 92% of the un-russeted green skin phenotype [4]. An interspecific cross between Chinese and Japanese pears identified a locus linked to the russet on LG8 [5, 6]. More recently it has been proposed that russet is a monogenic characteristic, controlled by a dominant gene [7]. This newly updated concept is based on bi-directional russet mutations between green and russet exocarp [8, 9]. These studies suggest russet and semi-russet are inherited independently, with russet obscuring semi-russeted exocarps when co-inherited.

Some *Malus* sp. (apple) also have varieties that have either complete russet such as ‘Merton russet’, or partial russeting disorders such as those found in ‘Golden Delicious’ [10]. A major QTL controlling apple russeting in ‘Renetta Grigia di Torriana” was reported on LG12 [11]. This study proposed that one major gene (*Ru*) controlled russeting and suggested an ABCG family transporter was the most likely *Ru* candidate. In another population, two further QTLs related to russeting were identified on LG2 and LG15 [12]. This study suggested that a SHN1/WIN1 transcription factor (*MdSHN3*) located on LG15 could be a candidate for controlling russeting in this cross. A study of skin development in cultivars predisposed to russeting disorders suggested that skin injury at anthesis caused a russet that persisted until maturity [10].

The most common commercial green kiwifruit *Actinidia chinensis* var. *deliciosa* ‘Hayward’ has a russeted peridermal skin. Less well known kiwifruit species have a wide range of fruit skin types that include epidermal or peridermal skins, or a combination thereof, together with a diversity of fruit hair types [13–15]. Other peridermal skinned kiwifruit include the yellow fleshed *A. chinensis* var. *chinensis* and red fleshed *A. chinensis* var. *chinensis* ‘Hongyang’, while the *A. arguta* and *A. melanandra* species have epidermal skin types (live with cuticle, with varying degrees of hairiness). A detailed study of the peridermal skin of *A. chinensis* [15] showed an intensively suberized tissue layer present, while the *A. arguta* fruit maintained an epidermal skin layer through fruit development. Cell wall changes were studied in *A. arguta* during fruit softening [14], with marked changes in cell wall staining throughout development showing that even a seemingly constant epidermal layer is undergoing constant change and adjustment.

In addition to a wide variety of skin types in kiwifruit species, there are also complexities of ploidy with many species demonstrating a range in ploidy from diploid to decaploid, making genetic studies more complex [16]. Currently the genetic understanding of periderm formation in fruit is limited to a few species. Here we aimed to further our understanding of the genetic loci of skin types by examining skin formation in a tetraploid inter-specific population obtained from a cross of a peridermal *A. chinensis* male with an epidermal *A. melanandra* x *A. chinensis* hybrid female. Using genotyping by sequencing (GBS) we constructed a genetic map to identify loci linked to skin-related traits.

## Results

### Inheritance of skin types

**Fig. 1:**
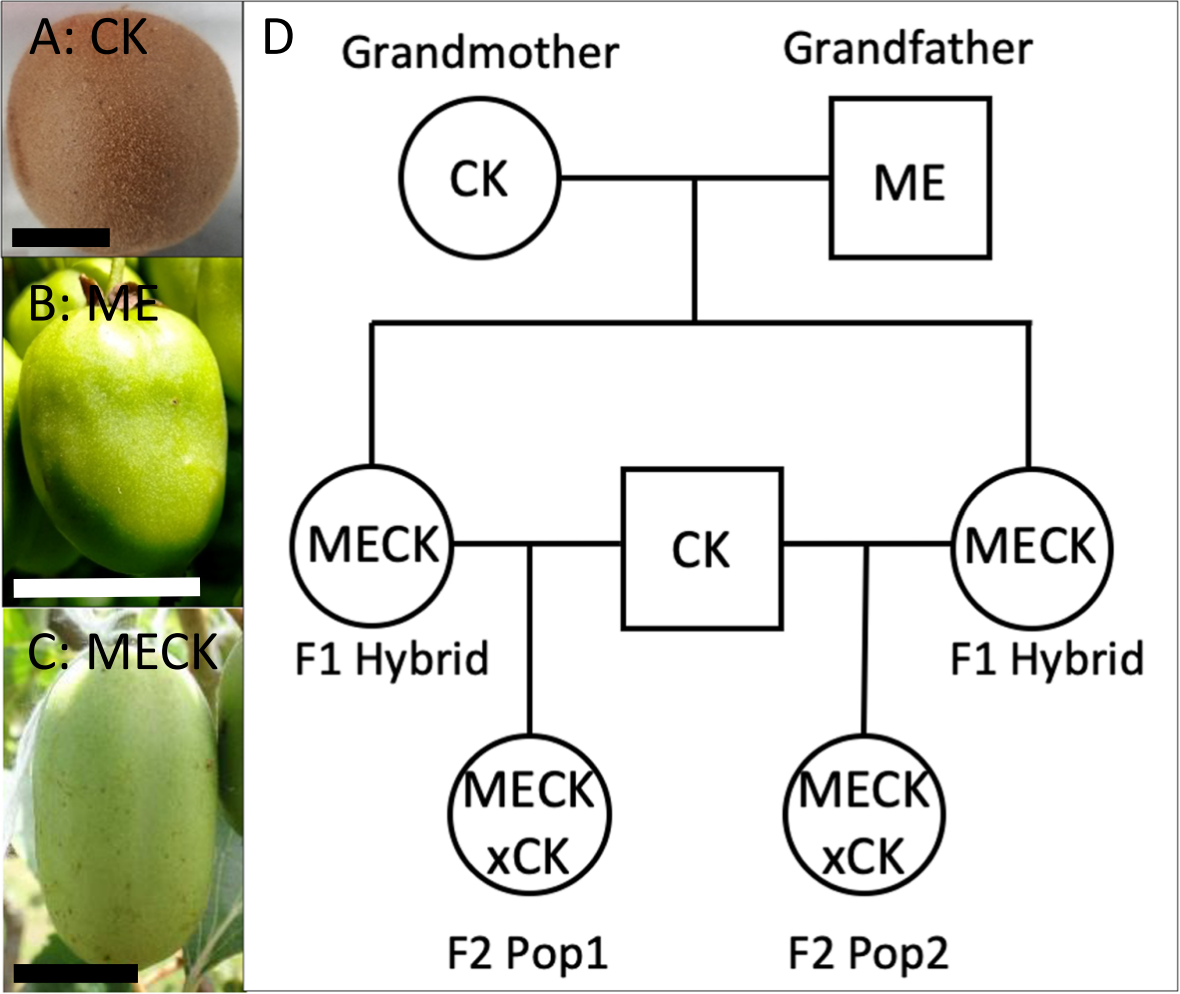
Parental material used in this study **A** Fruit of the *Actinidia chinensis* grandmother (CK) with peridermal exocarp; **B** Fruit of a female *A. melanandra* (ME) showing the sort of epidermal exocarp likely donated by the ME grandfather (actual grandfather was male and thus no fruit); **C** Example of fruit from one of the MECK F1 hybrids showing their epidermal exocarp. **D** Pedigree of populations used for this study. Boxes represent males and circles females. Scale bars in panels **A**/**B**/**C** indicate 20 mm

To assess the inheritance of skin types in kiwifruit, a tetraploid *A. melanandra* (ME) male was used for an interspecific cross to a tetraploid *A. chinensis* (CK) female. The female CK had a peridermal skin type (Fig. 1a). The fruit from sibling female (ME) have an epidermal (live) skinned phenotype (Fig. 1b) and a continuous covering of cuticle, with a low incidence of surface defects such as suberized layers and microscopic cracks.

**Fig. 2:**
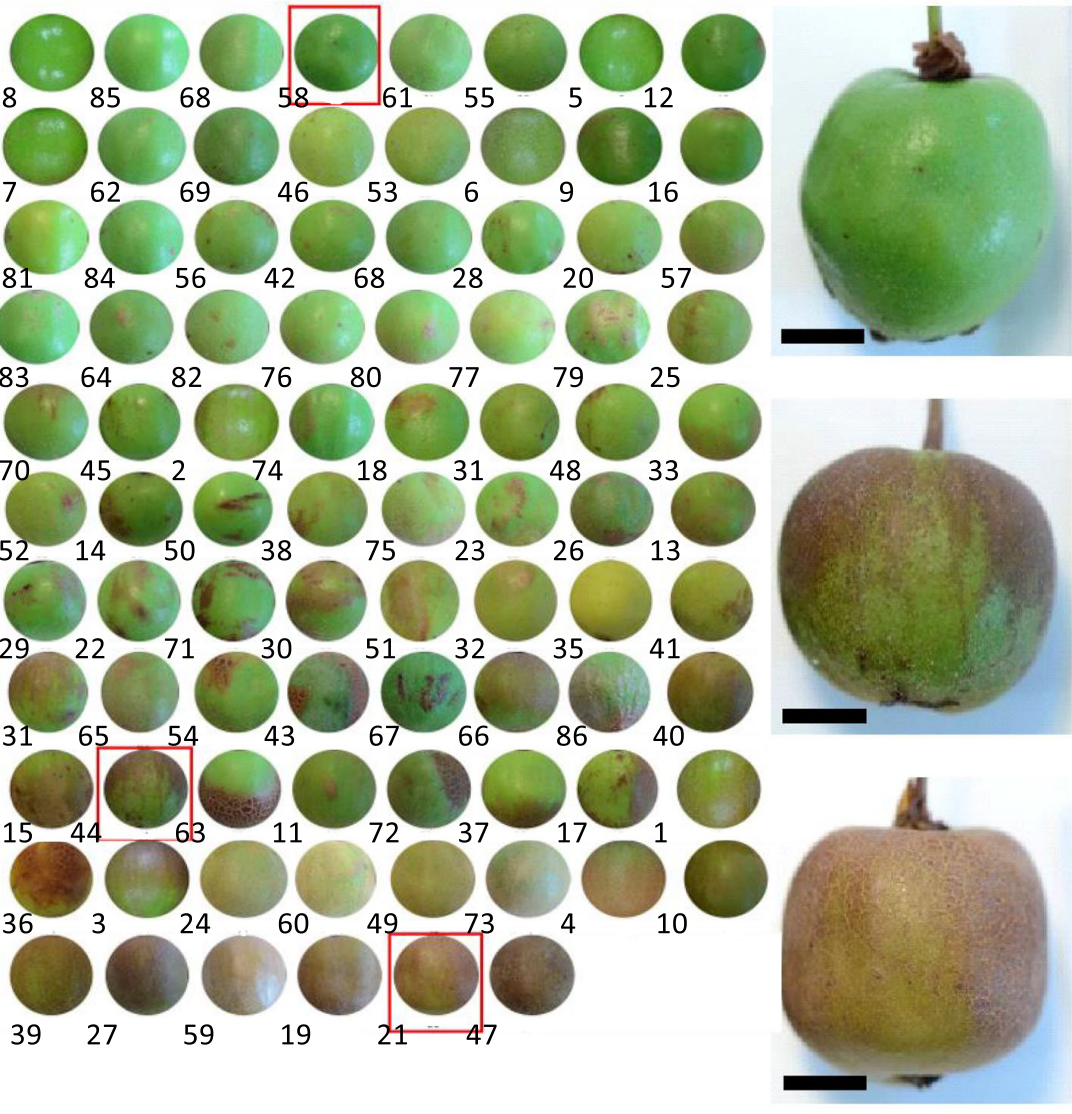
Segregation of skin traits in the backcross populations. Numbered circle images show representative regions of fruit skin from the segregating population to illustrate the range of epidermal, peridermal and russeted skins. Note, the circles are not representative of the true size or shape of each fruit. Larger images on right side for selected genotypes (corresponding to each of the red boxes on left) show the entire fruit for epidermal (top right), peridermal (bottom right) and russetted (middle right) examples. Scale bars indicate 10 mm

The fruit from female plants in the F1 progeny, (herein termed MECK) had a bright green epidermal skin suggesting this epidermal skin is a dominant trait (Fig. 1c). However, in the fruit from these MECK females there was an increased incidence of isolated regions of minor russet, suggesting a less robust epidermal exocarp in these fruit hence the reference to semi-dominance. While the ME fruit is typically a small spherical/ovoid berry, the F1 progeny were larger and more ovate/ellipsoid than their mother (Fig. 1c). As all the F1 progeny had an epidermal exocarp, in order to further investigate peridermal exocarp formation two MECK F1 sibling females were backcrossed to a single CK male resulting in two backcross populations each containing 25% of the original paternal ME genome. Both populations were found to segregate for peridermal and epidermal skin type (Fig. 2). Despite having two backcross populations, only one was found to have a sufficient number of fruiting females for a genetic study, of which there were a total of 76 vines. The MECK x CK backcross progeny demonstrated a range of intermediate skin types (Fig. 2), varying from having an epidermis covered in a thick cuticle to a densely layered peridermal exocarp. Six of the 76 vines were found to be clonal, leaving 70 backcross genotypes, 60 of which had predominantly epidermal exocarp and 10 had a complete peridermal exocarp. These were grouped into bins of 0–25%, 25–50%, 50–75%, 75–100% russet, with each of these bins containing 43, 10, 8, and 9 genotypes, respectively. When bulks of fruit were assessed from each of the 60 backcross individuals with some epidermal skin, all genotypes contained at least one fruit with minor russet marks. From this whole fruit analysis, the variation observed in the backcross progeny not only included the presence of russet but also differences in the appearance of the russet, with some russet patches smooth while others were scab like and dark brown. A large range of variation was also observed in the population for other traits such as hairiness and fruit size and shape (Supplementary data- section 1). A Pearson’s correlation of all traits measured found that fruit size had no significant impact on physiological traits measured (Supplementary data- section 2). The distribution of russet occurred in various positions (Fig. 2), without a clear dominance of pedicel dominant russet and instead a range of onset positions.

### Microstructural variation of skin types

**Fig. 3:**
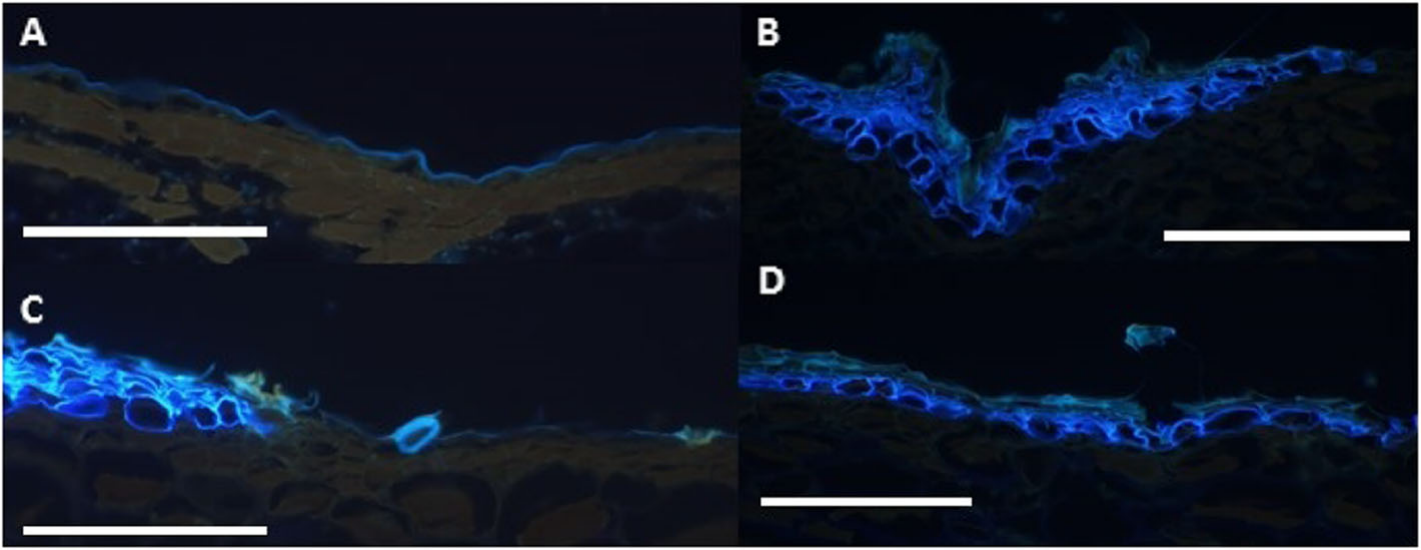
Auto fluorescence from toluidine blue stained sections of skins types seen in the backcross population. Scale bars represent 50 μM. **A** Epidermal skins with weakly fluorescing cuticle; **B** skins with russeted suberized cells and trichomes; **C** and **D** skins with russeted suberized cells; **D** russeted suberized cells covered with lignified cells. Suberin can be identified by bright blue fluorescence. Blue-green auto fluorescence indicates the presence of lignin. Auto fluorescence that occurred as a bright blue single layer above the L1 epidermal layer was taken as fluorescence of the cuticle

**Fig. 4:**
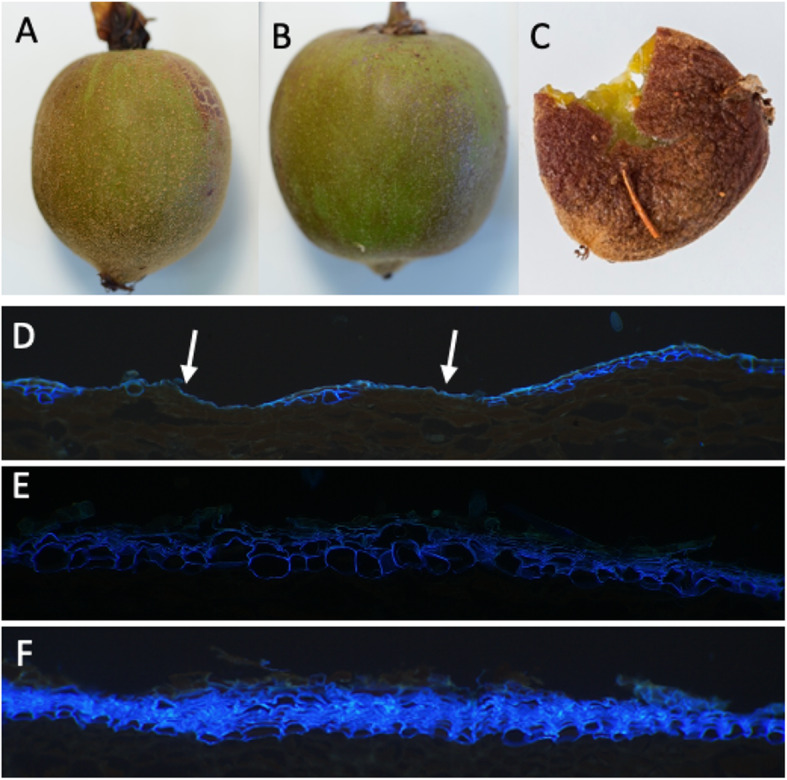
Three types of fruit with an apparent peridermal skin type. **A** to **C** show whole fruit; **D** section of fruit in **A** showing regions of epidermal skin layers (white arrows); **B** and **E** whole fruit and a skin section showing continuous periderm; **C** and **F** whole fruit (broken open to show thickness of skin), and skin section showing extreme numbers of cork cambial cells

A typical fruit from each of the 70 genotypes was selected for microstructural analysis. Despite collecting multiple fruit from many vines it was evident that only a handful of vines had multiple mature fruit, thus while it reduces the power of this study only one fruit of each genotype was examined using microscopy. Where possible, a region with both russeted and epidermal exocarp was selected. Sections were stained with toluidine blue to allow identification of cuticle, suberized layers and lignified layers by auto fluorescence and different staining patterns. Suberin deposition was identifiable by bright blue fluorescence, usually in sub-epidermal layers, and indicated cells that can be considered on a track towards programmed cell death. Blue green auto fluorescence suggests the presence of lignin. Auto fluorescence that occurred as a blue single layer above the L1 epidermal layer was taken as fluorescence of the cuticle. The microstructural examination revealed a much more complex range of phenotypes than originally anticipated. The skin types of some genotypes appeared to be completely different from either parent, with a large range in the numbers of periderm layers and evidence of lignification of the skin surface, on top of the expected suberization (Figs 3 and 4).

**Fig. 5:**
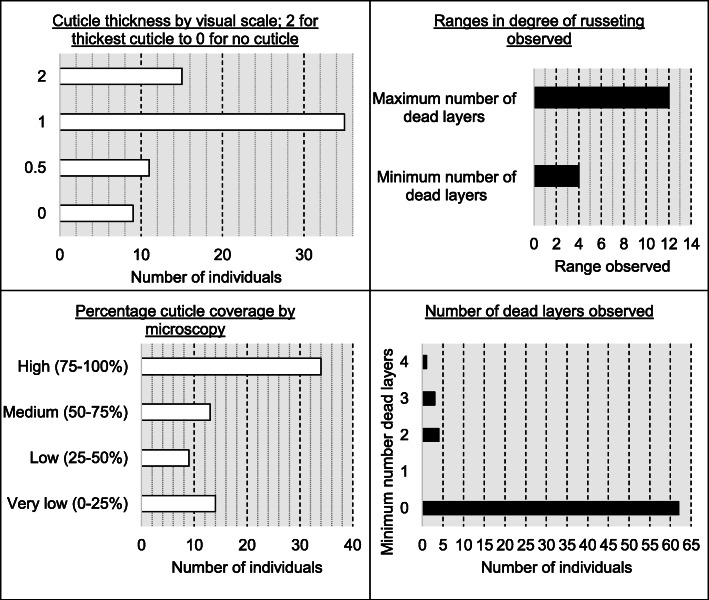
Summary of cuticle and periderm scores for the 70 genotypes used in this study. Data is derived from microscopy study, see Table 2 for further descriptions of phenotyped traits

Of the 70 fruit, eight had total peridermal coverage when observed at the cellular level (with two of the previously characterized peridermal fruit having small regions of epidermal skin only visible at higher magnification; e.g. Fig. 4d). The remaining 62 genotypes had at least one point of observable cuticle. The cuticle thickness was scored when possible (because the cuticle was often damaged during sectioning). A visual scale of cuticle thickness separated the population into four bins: 2, 1, 0.5 and 0; consisting of 15, 35, 11 and 9 individuals respectively. The relative degree of cuticle coverage observed by microscopy (percentage length of fruit surface which had cuticle cover; 0 to 100% scale) was assessed as a continuous trait, but it consisted of 14, 9, 13, and 34 individuals, respectively, if binned into the following four categories: very low cuticle coverage (0–25%), low cuticle coverage (25–50%), medium cuticle coverage (50–75%) and high cuticle coverage (75–100%) (See Fig. 5 for visual summary).

The degree of russeting/cork meristematic growth varied significantly within the population: the maximum number of dead layers observed by microscopy varied from 0 to 12 while the minimum number of layers varied from 0 to 4. Within the 70 individuals comprising the backcross population, 62 had a minimum number of dead layers of 0, whereas no genotypes had only one cell layer, four genotypes had a minimum of two cell layers, three genotypes had at least three cell layers, and one individual had at least four cell layers of periderm across all regions of the exocarp. Thus any genotype with a minimum number of layers equal to or greater than two were considered to have a phenotype of fully peridermal exocarp as described above.

The maximum number of peridermal layers/degree of russeting were split into four bins 1–3, 4–6, 7–9, or 10–12 dead cell layers, which included 12, 37, 16 and 5 genotypes respectively. Other notable features studied by microscopy relate to the formation of trichomes. All individuals (including parents) were found to contain trichomes, however the backcrossed population displayed sub-types ranging from single- to multi-celled, suberized and lignified. Suberized trichomes were represented by 58/70 individuals and lignified trichomes were represented by 65/70 individuals (Supplemental data 1).

### Correlation of macroscopic and microscopic phenotype scores

To test whether there was any correlation between the traits measured, a correlation matrix for each trait was produced. It was found that peridermal phenotype correlated positively with russeting score and the minimum number of dead cell layers, with a Pearson correlation coefficient of 0.85. Cuticle coverage was negatively correlated to peridermal phenotype with a Pearson correlation coefficient of -0.73. A negative correlation between cuticle coverage and the number of cork meristem layers was also observed (Pearson correlation coefficient -0.86). The macroscopically scored russeting percentage was found to negatively correlate with percentage cuticle coverage (Pearson correlation coefficient -0.74), while positively associating with the minimum number of dead layers and the mean number of peridermal layers (Pearson correlation coefficients 0.70 and 0.70 respectively) (Supplemental data 2). There were no significant correlations between other traits such as fruit size and presence of trichomes with periderm or russeting.

### Genotyping by sequencing and genetic map construction

Genotyping by sequencing (GBS) libraries were constructed from DNA extracted from both MECK and CK parents and individuals from the backcrossed population. An average of 5,005,601 sequencing reads for each individual were obtained from each library. Using the *Actinidia chinensis* Red5 genome [17], 92.2% of sequence reads were successfully mapped. A total of 1,389,348 variants were detected, which comprised 52,475 single nucleotide polymorphisms (SNP).

An analysis of the SNPs used in this study found the proportion of SNPs calling across the population to be 88.7% and the median of the mean sample depth was ~ 230x (Supplemental data 3). The SNP call rates and their minor allele frequencies (MAFs) were calculated (Supplemental data 3). The mean co-call rate (for sample pairs) was 0.7934 while the min co-call rate (for sample pairs) was 0.0234. A plot was developed using the KGD package [18] where Hardy-Weinberg disequilibrium was plotted against MAF showing the density of SNP depth. SNP depth had a median value of 105.9 while the mean-self relatedness (G5 diagonal) was at 1.9320 (Supplemental data 3).

Linkage maps of the four homologous chromosomes (CHR) for each parent were constructed using segregating simplex x nulliplex SNP markers that occurred in at least 80% of the individuals used. The linkage map was produced using Joinmap4.0 [19]. In total, 7,568 and 6,062 markers were used for linkage map construction for the female (MECK) and male (CK) parent, respectively. The MECK female map was made up of 157 linkage groups (LG), spanning 8,139 cM in total, with an average distance of 51.84 cM per LG. The map had an average of one SNP marker every 18.23 kb, with the largest gap being 1,320 kb. The CK male map was comprised of 90 LGs, spanning 5,747 cM in total. This represented an average distance of 217.52 kb between SNPs, with the largest gap between markers being 701 kb.

Using these linkage maps, quantitative trait loci (QTL) analysis was conducted for each of the measured traits. These were tested for significance using the non-parametric Kruskal-Wallis analysis on each marker using MapQTL v5.0 [20].

### QTLs related to whole fruit peridermal/epidermal skin (periderm)

**Fig. 6:**
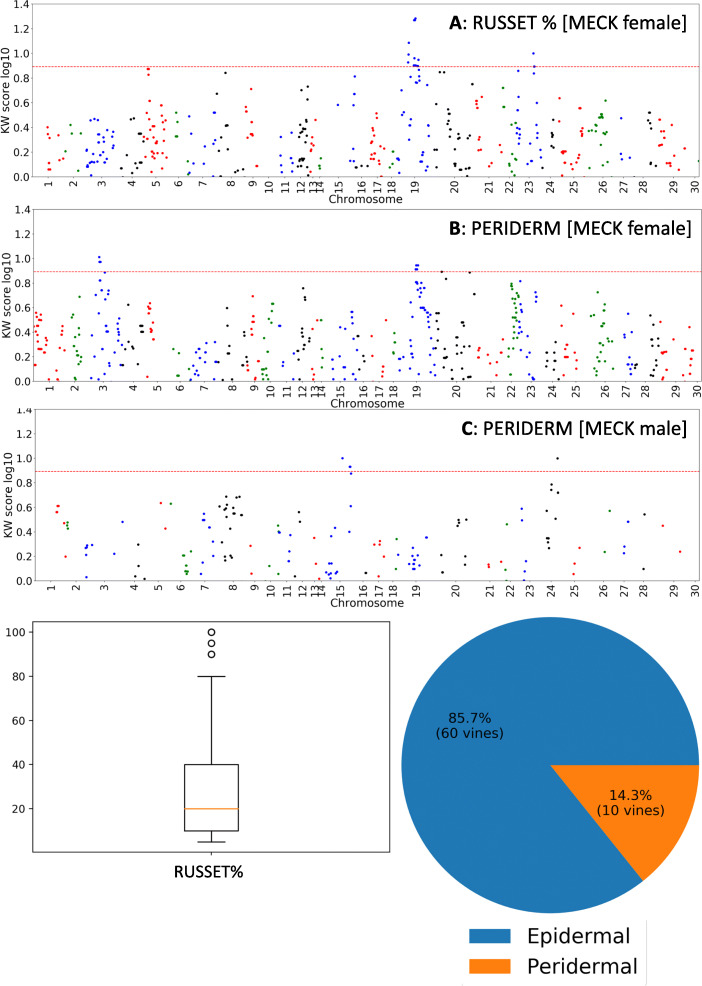
Non parametric Kruskal-Wallis (KW) analysis of genetic markers arranged in order of chromosomal location that associated with percentage coverage of russet (RUSSET %) in panel **A**, and the appearance of native periderm (PERIDERM) in panels **B** and **C** for mother and father maps respectively. A KW score of > 7.8 = *p* < 0.005, and lies above dotted line. The summary phenotype statistics for the trait measured are presented below

Two maternal QTLs were identified on LG154 and LG102 and each associated with three and four markers, respectively (*p* < 0.005). These markers were physically located on CHR3, positioned between 13,504,746 bp and 13,504,785 bp and CHR19 between 12,859,098 bp and 12,859,243 bp) (Fig. 6b, Table 1). Paternal QTLs were identified on LG39 and LG85 (*p*<0.005) which were physically located on CHR15 between 10,394,929 bp and 13,136,869 bp, and LG1 (*p*<0.005) which was physically located on CHR24 at position 12,647,436 bp (Fig. 6c, Table 1).

**Table 1:**
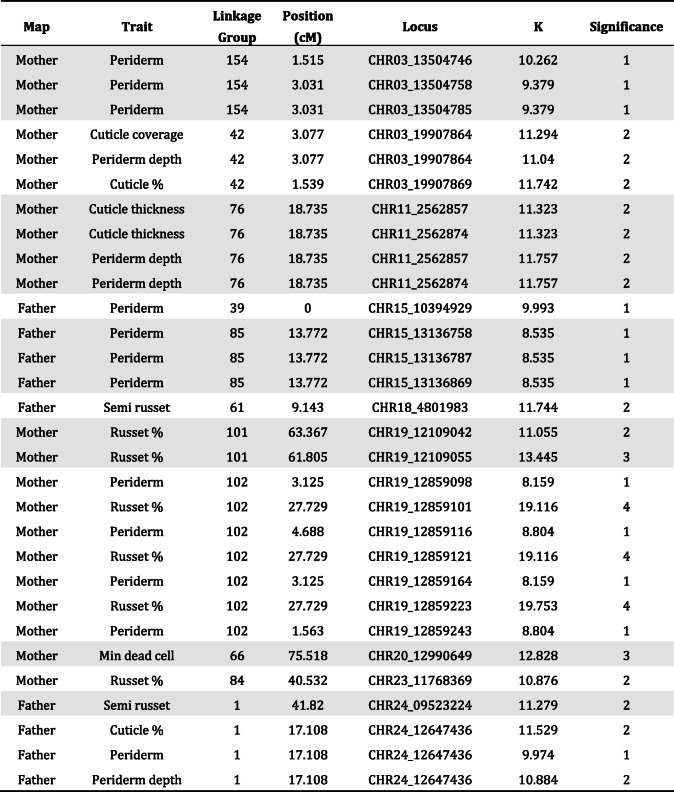
Details of strong skin related QTLs found in this study. Note: Shading is to aid reader by grouping according to chromosomal locations

K values significance probability thresholds (*p*<): 1: 0.005, 2: 0.001, 3: 0.0005, 4: 0.0001

Other skin related QTLs associated with significant markers for other skin related phenotypes are presented in Table 1 (significant QTLs at *p* < 0.001 presented) and QTLs with a minimum threshold of K > 7.8 (*p* < 0.005) are presented in Supplementary data- sections 4 and 5. Linkage map plots are presented in Supplementary data- sections 6 and 7.

### QTLs related to russeting (russet %)

**Fig. 7:**
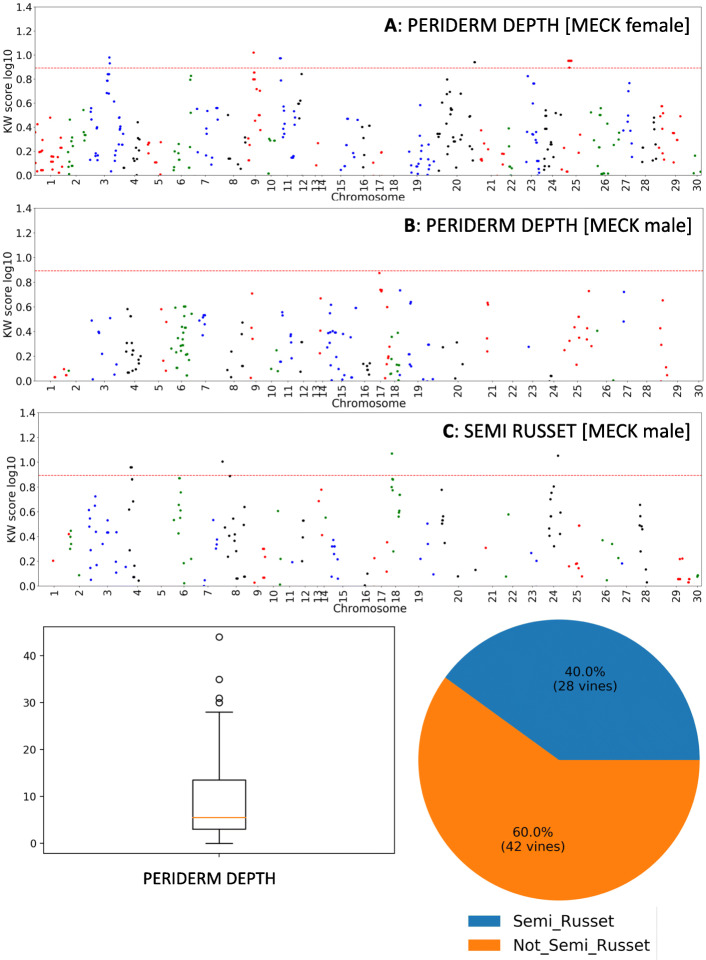
Non parametric Kruskal-Wallis (KW) analysis of genetic markers arranged in order of chromosomal location that associated with the mean number of peridermal layers across the fruit surface (PERIDERM DEPTH) for mother (panel **A**) and father maps (panel **B**), and semi russet appearance (SEMI RUSSET; panel **C**). A KW score of > 7.8 = *p* < 0.005, and lies above dotted line. The summary phenotype statistics for the trait measured are presented below

A strong QTL derived from maternal parent MECK was identified associated with russeting on LG102 on CHR19 with a K score of 19.753 (*p* < 0.0001). The most significant markers were positioned between 12,859,101 and 12,859,223 bp (Fig. 6a, Table 1). Russeting was also mapped to LG84 (*p* <0.005) with the marker with the highest K score on CHR23 at 11,768,369 bp (Fig. 6a). QTLs for mean number of peridermal layers (Periderm Depth) mapped to the MECK maternal map (Fig. 7a) with strong QTLs located on LG42 (CHR3) (most significant marker located at 19,907,864 bp) and LG76 (CHR11; most significant markers positioned at 2,562,857bp and 2,562,874bp). From the CK paternal map one significant QTL controlling the number of peridermal layers was identified on LG1 located on CHR24 at 12,647,436 bp (Table 1). Two QTLs for semi-russet were found in the CK paternal map, one on LG1, which is physically located on CHR24 and with the most significant marker positioned at position 9,523,224 bp, just over 3 Mbp away from the QTL associated with the number of peridermal layers, and one QTL on LG61 which aligned to CHR18 at position 4,801,983 bp (Fig. 7c).

### QTLs associated with cuticle formation

**Fig. 8:**
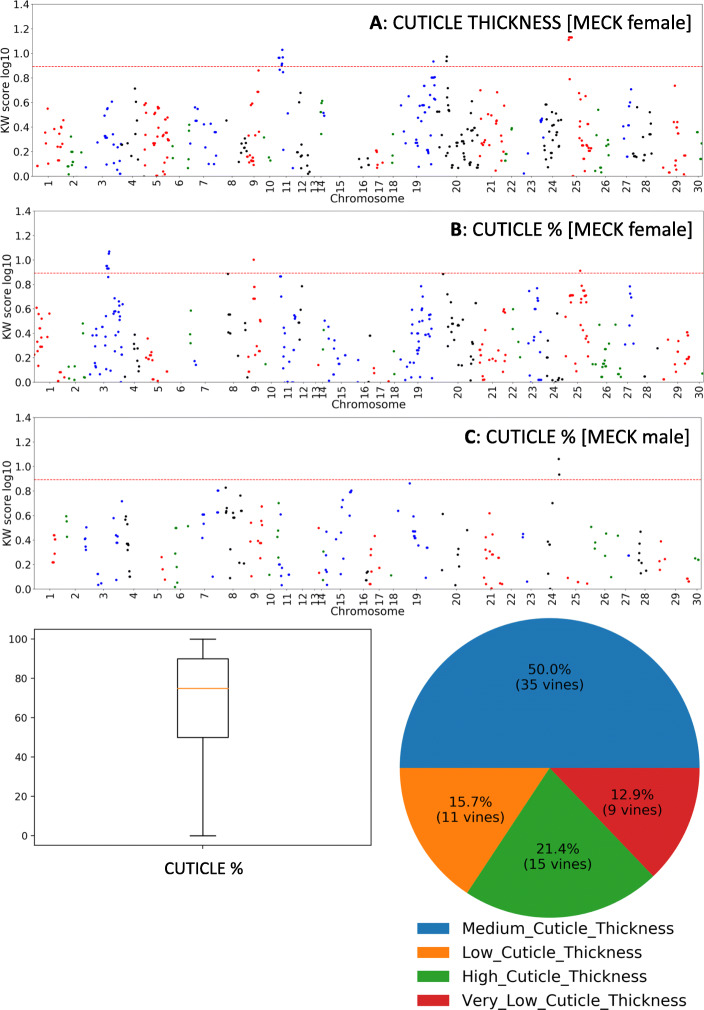
Non parametric Kruskal-Wallis (KW) analysis of genetic markers arranged in order of chromosomal location that associated with cuticle-related traits including thickness of the cuticle (CUTICLE DEPTH = Cuticle thickness; panel **A**) and cuticle coverage over fruit surface (CUTICLE % = cuticle coverage; panels **B** and **C** for mother and father maps, respectively). A KW score of > 7.8 = *p* < 0.005, and lies above dotted line. The summary phenotype statistics for the trait measured are presented below

For cuticle related traits, cuticle thickness had maternal MECK QTLs on LG76 and LG120 (Table 1 and Supplementary data- section 4). The most significant markers from LG76 were physically located on CHR11 at positions 2,562,857 and 2,562,874 bp, while the markers on LG120 were physically located on CHR25 at positions 6,351,221 to 6,351,361 bp (Fig. 8a, Table 1 and Supplementary data-section 4). The CK father had a weaker QTL associated with cuticle thickness on LG35 which was located on CHR26 at position 2,817,564 bp (Supplementary data- section 4).

For cuticle coverage (Cuticle %) the maternal MECK map had a QTL on LG42 which was physically located on CHR3 at position 19,907,864 bp and with lower significance QTLs (LG42 and LG79) on CHR3 and CHR25 (15,529,996 bp), respectively (Fig. 8b, Table 1, and Supplementary data- section 4). Cuticle coverage inherited from the CK father included a QTL on LG1 physically located on CHR24 positioned at 12,647,436 bp (Fig. 8c and Table 1). The trait ‘crack‘ (micro-cracks in cuticle observed under microscopy; Supplementary data 1), had two weak paternal CK QTLs: LG81 (CHR26) and LG8 (CHR20) (Supplementary data- section 4).

QTLs associated with trichome number and density, suberization, lignification and fruit length can be found in Supplementary data- section 5. In summary lignified trichomes (ligtri) mapped to CHR2 (Mother map); suberized trichomes (subtri) mapped to CHRs 1, 23 and 25 (Mother map), and CHRs 5, 6 and 10 (Father map); hair density (hairy) mapped to CHRs 5 and 9 (Mother map) and CHRs 9, 28 and 29 (Father map); fruit cross-sectional area (area) mapped to CHR7 (Mother map) and CHR19 (Father map), and fruit length (length) mapped to CHRs 11 and 29 (Father map).

### Location of pericarp candidate genes from the literature in relation to QTLs identified herein

A number of studies have listed a range of gene candidates involved with secondary meristem formation, russet periderm, cuticle formation, and suberin and cuticular wax biosynthesis [11, 12, 21–41]. When their peptide sequence was BLASTP searched in the recently published kiwifruit genome [17], orthologous kiwifruit genes were found to reside in close proximity to all the mapped marker locations (Supplementary Table 1). A common trend is that multiple candidates lie within 3 Mbp for all of the QTLs. For one of the two markers identified on CHR3 (13.50 Mbp; Mother: Periderm), the proximal candidates are all suberin related while for the QTL at 19.59 Mbp (Mother:Cuticle coverage/Mean periderm depth), an orthologue of KCS11, a 3-ketoacyl-CoA synthase involved in very long chain fatty acids which are components of cuticular wax, is only 0.31 MBp away. For the ‘Mean periderm depth’ and ‘Cuticle thickness’ QTLs identified on CHR11 (both Mother map), the closest candidate was 0.17 MBp away. Its best reverse BLASTP match was to UBIQUITIN-SPECIFIC PROTEASE 16 (UBP16; *AT4G24560*) which is linked to cell proliferation [42]. Other notable proximal candidates include *WOX4-like* and cuticle wax synthesis associated *KCS4-like* orthologues. Chromosome 15 harbors ‘Periderm’ QTLs (Father map) at 10.39 and 13.13 Mbp and a compelling candidate is located between the QTLs at 11.43 Mbp which is a *CER1-like* gene involved in cuticular wax synthesis. The two other candidates listed (in Supplementary Table 1) are less compelling because their best reverse BLASTP matches are to different proteins than the original input query. Several transcription factor type candidates lie close to the Chr15 13.13 Mbp marker, but their best reverse BLASTP matches are to different proteins than the original input query making them less compelling.

For the ‘Semi russet (Father) QTL on CHR18, three compelling candidates are located within 1.9 Mbp, and include *AtMYB85* and *AtMYB103-like* MYBs (both associated with secondary wall biogenesis), and a *BDG*/*CED1-like* an α-β hydrolase which is required for normal cuticle formation [43].

CHR19 harbors QTLs for ‘Russet’ and ‘Periderm’ (both from Mother map) at 12.10 and 12.85 Mbp, respectively, and key cuticle synthesis associated candidates reside in close proximity. One of these is a KAS1-like orthologue (12,405,229 … 12,414,036 bp), where KAS1 is crucial for fatty acid synthesis [23]. Two other cutin synthesis related genes also lie in close proximity (Supplementary Table 1).

For the ‘Min depth’ QTL (minimum number of periderm layers) observed on CHR20, two MYBs (*AtMYB52* and *AtMYB94*-like genes) locate nearby as does as another α-β hydrolase (required for normal cuticle formation). Over expression of *MYB94* causes activation of cuticular wax biosynthesis [44], however the best reverse BLASTP matches to both MYBs are to different MYBs: *AtMYB117* and *AtMYB60*, respectively, and these have less clear relevance to exocarp development.

For the ‘Russet’ QTL (Mother map) on CHR23 a compelling candidate is a poplar *PtSHR2B SHORT-ROOT-like* gene orthologue located only 0.07 Mbp away; in poplar *PtSHR2B* is involved in regulating phellogen activity [45]. Other notable candidates include a fatty acid reductase (*FAR3-like*), involved in cuticular wax biosynthesis [21], and an ATP binding cassette transporter *ABCG34-like* gene. Indeed a similar ABCG family transporter gene was suggested to be the major determinant of the apple skin russetting development gene *Ru* [11] and the ABCG transporter ABCG1 is required for suberin formation in potato tuber periderm [46].

Chromosome 24 harbors a ‘Semi russet’ (Father map) QTL at 9.52 Mbp and ‘Mean periderm’, ‘Cuticle coverage’ and ‘Periderm’ (all from Father map) QTLs around 12.64 Mbp. Two compelling candidates are closer to the 9.52 Mbp QTL and include *ANAC078-like* and *REVOLUTA-like* genes. *ANAC078* is the Arabidopsis match to a potato Nam-like protein transcription factor (GenBank: HO209042.1), which was identified as a potential candidate gene involved regulating periderm development [47], while REVOLUTA regulates meristem initiation at lateral positions [48]. Also on CHR24 and closer to the ‘Cuticle coverage’ and ‘Periderm’ QTLs around 12.64 Mbp are a GPAT5-like gene (BLASTP expect=0), involved in biosynthesis of suberin polyester [49], and a *WOX5-like* gene. WOX5 interacts with CYCD6;1, and CASP1 with SCARECROW and SHORT-ROOT during formation of tissue specific higher order transcription factor complexes during meristem induction [50, 51]. There is the possibility that this sort of process could be happening during phellogen formation in fruit exocarp [41].

## Discussion

In this study we characterized the inheritance and genetic control of peridermal skin formation in kiwifruit skin. This mapping study was limited by both the number of individual vines and the number of fruit per vine. The size of some vines prohibited triplicate sampling, and in some cases led to less mature fruit being sampled. Periderm formation was most clearly indicated by microscopy, which could identify periderm in both mature and semi mature fruit. The number of linkage groups detected was high as the sequenced kiwifruit were tetraploid and additionally because our methods mapped all homologous chromosomes separately. In this case the small population size did not preclude the identification of highly significant markers.

The presence of epidermal exocarp was a semi-dominant trait passed down from the *A. melanandra* grandfather in the F1 generation, however, this dominance was broken down into a continuous spectrum of phenotypes within the backcross population. The population displayed a large variation in both cuticle thickness and cuticle coverage, and notably those fruit with weak and/or thin cuticles tended to develop cork meristematic layers of greater thickness and with greater coverage over the fruit surface. This suggests that fruit periderm formation is a continuous russeting of the skin rather than a developmental switch for periderm formation, and this russeting is associated with the robustness of the cuticle [52]. This mechanism appears to be similar to that found in russet apples [12] and russet pears [8, 9]. Environmental factors could skew sporadic periderm formation (russeting) [53] and we only collected data for one season so this should be taken into account when assessing the QTLs.

Peridermal skin formation (continuous russeting) is recessive, suggesting a loss of gene function is needed to develop dead skinned fruit. There appear to be significant russeting loci on CHR3, CHR19, and CHR23, and one on cuticle formation on CHR3 in the maternal MECK genetics and QTLs on CHR15, CHR18 and CHR24 from the paternal genetics, indicating there are multiple chromosomal locations contributing to skin quality. This is manifested in the range of phenotypes observed. The genetic control of skin formation in apple and pear was initially proposed as a two-factor model [3], however this has been re-examined and it has been suggested that there is one major dominant gene and a variety of other influential genetic regions [7]. This study in kiwifruit has not resolved this, and in kiwifruit it appears to be a complex multi-loci trait that is associated with cuticle synthesis and russeting.

Cross referencing to previously suggested candidates in past literature found compelling candidates for all of the strong skin-related QTLs found in this study. Cutin and suberin synthesis genes feature prominently (CHR3/15/19/23) and some compelling potential regulatory gene candidates have been identified as well. These require further investigation and verification.

Ultimately the type of skin/exocarp relates specifically to cuticle strength and russeting, though the mechanism by which russeting is initiated is still poorly understood. Previous studies have shown that cork and russet appear to be initiated in specific zones, suggesting that this may be associated with wound signals caused by micro cracks in the expanding fruit [1, 54, 55]. There is also evidence that water exposure of cuticles (that are strained during fruit growth) results in formation of microcracks, which in turn causes formation of a periderm [53]. Indeed russeting also results from skin damage in kiwifruit. These various factors highlight a key question regarding exocarp formation: does a reduction in cuticle integrity lead to periderm formation or does the initiation of periderm formation lead to reduced cuticle?

## Conclusions

The skin type of kiwifruit was genetically linked to multiple genetic regions, and for all the QTLs identified herein there are multiple orthologues of previously reported potential candidate genes residing in close vicinity which could account for most of their effects. Functional characterization is required to validate the role of these candidates within QTL regions. It is possible that there are yet to be identified additional controlling genes residing in those regions’ QTLs as well. The physiological traits measured using light microscopy confirmed the segregation of skin type within a kiwifruit population with visibly perturbed skin formation within the backcross progeny. Correlations between physiological traits highlight the tendency of fruit with more cuticle coverage to have less dead cell layers. This is unsurprising because multiple russeting studies demonstrate that a thin damaged cuticle is readily replaced by a more plastic periderm [1, 55]. The tight correlation in respect to kiwifruit highlights the intimate connections between tissue layers, and highlights a potential biological limit whereby a plant will always form periderm where the cuticle has degraded.

## Methods

### Plant material

All plants were generated and grown at The New Zealand Institute for Plant and Food Research Ltd (PFR) Motueka research site, Tasman, New Zealand. *Actinidia melanandra* and *A. chinensis* var. *chinensis* from PFR’s germplasm collection were used in this study. All plant material was grown under standard orchard conditions on a pergola system, where vegetative canes were grown up 45 degree strings and in the following year were pulled down to the horizontal and then grown on to flowering and fruiting. A tetraploid *A. melanandra* male (ME) was crossed to a peridermal skinned female *A. chinensis* var. *chinensis* (CK). A small population of F1 (MECK) females were saved and two epidermal skinned females were subsequently used as females to create a backcross using *A. chinensis* var. *chinensis* pollen (Fig. 1). One of the resultant MECK x CK backcross populations of 70 individuals was examined and used for mapping.

### Phenotyping

The fruit used in this study were harvested approximately 2–4 weeks before commercial harvest when the fruit had a visually mature exocarp tissue. This coincided with the major harvest of mid-March 2016 and additional progeny were collected the following season in the last week of March 2017 to complete the set of 70 genotypes (at this stage not all of the population had progressed past the juvenile stage to flower and set fruit). Fruit availability dictated that of the 70 genotypes, there were 64 triplicate, 3 duplicate, and 3 single biological replicates. Female vines were analyzed for various fruit characteristics including macroscopic features such as skin type (epidermal, russeted or fully peridermal), hairiness and fruit size (three replicates if possible, but some genotypes only had one fruit at harvest). Color photographs were taken of each fruit in a photography studio, and fruit size was measured using a digital caliper.

Microstructural features were studied using light microscopy (trait types listed in Table 2, includes macro structure measured traits). The coverage of cuticle and its thickness was focused on the L1 tissue layer rather than cuticle content in the sub epidermis. The cuticle thickness measurements (by microscopy) were grouped into four bins of 0/1/2/3 (score 0: cuticle not present to 3: thickest cuticle). Sample collection involved using a scalpel to slice transverse sections of the fruit skin (~2cm), including a minimum of three technical replicates for each fruit. The fruit sections were vacuum infiltrated with fixative (formaldehyde 4%/ ethanol 50%/acetic acid 5%) in small scintillation vials. These sections were sequentially dehydrated in ethanol and embedded in wax blocks, then sliced into at least five thin ~5 micron sections using a microtome and then viewed under a light microscope, as described in a previous study of softening in *A. arguta* kiwifruit exocarp [14].

**Table 2:** Phenotypic scores measured during examination of exocarp under 200x magnification as well as macrostructure traits (indicated by ‘macro scale’) using images of whole fruit

Phenotypic Trait	Details
Max. dead cell	Maximum number of peridermal layers observed
Min. dead cell	Minimum number of peridermal layers observed
Cuticle %	Coverage of cuticle over fruit exocarp (0 to 100% scale)
Periderm depth	Mean number of peridermal layers
Trichome density (0:3)	0,1,2,3 scale; 0 no trichomes to 3 high density
Lignified trichome (0/1)	scored 0 (no lignin) or 1 (lignified)
Suberized trichomes (0/1)	scored 0 (no suberization) or 1 (suberized) trichomes
Cuticle thickness (0:3)	0,1,2,3 scale; score 0 (cuticle not present) to 3 (thickest cuticle)
Microcracking (0:3)	0,1,2,3 scale; score 0 (no microcracks) to 3 (extensive microcracking)
Periderm	Epidermal (0) or peridermal (1) phenotype (> 2 peridermal cell layers)
Russet %	Percentage of surface covered with periderm (macro scale)
Semi Russet	Partial russet ranging from 20 to 80% (1) or not semi russet (0) (macro scale)

Sections were stained with toluidine blue (0.5% [w/v] in 0.1% (w/v) sodium carbonate pH 11.1) before being mounted on glass slides. Sections were observed using bright field and epifluorescence on an Olympus Vanox AHTB3 compound microscope with a 100 W halogen light source for bright field observation and the AH3-RFC Reflected Light Fluorescence Attachment that employs a 200W ultra-high pressure mercury burner as its light source for fluorescence observation. In bright field mode when an objective power was selected the condenser elements changed automatically, all other adjustment were manual. Illumination intensity was adjusted by a series of neutral density filters. Observations were carried out using Olympus DPlanApo objectives (x10 NA 0.4, x20 NA 0.7, x40 NA 0.85). Images (Figs. 3 & 4) were captured using ultraviolet fluorescence (excitation 330–385 nm, dichroic mirror 400 nm, emission ≥420 nm – Olympus BH2-DMU filterset). For image capture a Photometrics CoolSnap colour camera (Roper Scientific Ltd, Tucson, Arizona) was connected to the microscope using a NFK 1.67x photo eyepiece and 0.3x C-mount adapter (Olympus MTV-3). Images were acquired using RS Image capture software (Roper Scientific Ltd, Tucson, Arizona).

The auto-fluorescence of lignin, cutin, wax and suberin under ultraviolet light was used to aid in the classification of the exocarp structures within the MECK x CK segregating population. The histological staining patterns pronounced by toluidine blue were related to previous observations regarding the localization of suberin and lignin within kiwifruit exocarp [15].

### DNA extraction and GBS library construction

Young leaf tissue from field grown plants was harvested into 1.5-mL microfuge tubes (Eppendorf, Germany) and snap frozen in liquid nitrogen. Total genomic DNA was extracted using a Qiagen Plant DNeasy Plant Mini kit (Qiagen, Hilden, Germany) following the manufacturer’s protocol. DNA quality was quantified and checked for integrity using a Fragment Analyser (Advanced Analytical, United States). Samples with DNA of less than 10 kilo base pairs were rejected and re-extracted.

Random tagging genotyping by sequencing (rtGBS) was completed using a published protocol (dx.doi.org/10.17504/protocols.io.kzmcx46) with several alterations detailed below. The restriction enzyme chosen was *Pst*I, known to cut at CTGCA^G sites with a predicted frequency of 2.44x10^-4^ within the kiwifruit (*Actinidia* sp.) genome. The *Pst*I enzyme has 366,211 cut sites within the reference genome at ~244 sites per mega bases. The libraries were sequenced across two lanes of Illumina HiSeq2000 sequencing. In total, 76 GBS libraries were sequenced with 100 bp paired-end reads over two lanes of HiSeq Illumina sequencer (Illumina Inc) using the Australian Genome Research Facility (agrf.org.au) as the sequence provider. Samples in lane A had a total of 249,263,071 reads equating to 50.35 giga base pairs while lane B had a total of 242,966,761 reads equating to 49.08 giga base pairs, making up 99.43 giga base pairs in total. The lowest mapping score was 86.25% while the highest was 99.26%.

### Bioinformatics

Sequencing reads were trimmed with Fastx trimmer (http://hannonlab.cshl.edu/fastx_toolkit/index.html), and quality tested with Fastqc (http://www.bioinformatics.babraham.ac.uk/projects/fastqc/) and Multiqc (https://multiqc.info/). Reads were aligned to the kiwifruit Red5 genome [17] (GenBank accession NKQK00000000; genome version NKQK00000000.1; Assembly Name: Red5_PS1_1.69.0) using Bowtie 2 [56] which was set up to utilize paired-end reads. Sequencing blocks of less than 500 bp were removed as well as insertion/deletions. Freebayes [57] was used to call SNPs that had a minimum read depth of 50. SNP calling results were merged together and only SNPs with a read depth of 5000 were retained. The compressed variant call file for GBS mapping [634.1 MB], named ‘CKMEXCK_PS1.1.69.0_K857.vcf.gz’ can be accessed at 10.5281/zenodo.4722054. Furthermore, following these basic filters, reads were then filtered identifying genomic regions of high conservation shared amongst divergent species in the kiwifruit genome. In this approach, researchers identify genomic regions of high conservation shared among divergent lineages, design synthetic oligonucleotide ‘baits’ that are complementary to these regions, hybridize genomic libraries to these oligonucleotide baits, ‘fish’ out the hybridized bait + library structure, remove the bait sequence and sequence the remaining pool of enriched, targeted DNA.

### Genetic map construction and QTL analysis

The population is tetraploid and hence there are four alleles segregating at each locus. The SNPs that were homozygous across all four alleles (0/0/0/0) were considered as haploid homozygous, whereas any sign of heterozygosity e.g. 0/1/1/1, 0/0/0/1, 1/2/2/2 was considered a heterozygous SNP. For each parent only the Simplex X Nulliplex (e.g. 0/0/0/1 x 0/0/0/0) SNPs were retained.

Parental genetic maps were constructed using the double pseudo-testcross mapping strategy [58]. The linkage analysis and the map construction were performed using JoinMap® v3.0c [19] with a LOD score of 5 for grouping and Kosambi’s function for genetic distance calculation. QTL analysis was performed with MapQTL® version 5.0 [20]. The data distribution was verified for each trait before QTL analysis: non-normal and normal distributions were analyzed using primarily the nonparametric Kruskal-Wallis test [59] and an interval mapping (IM) analysis, respectively (Supplementary data 3). For the IM analysis, the LOD threshold for significance of a QTL was calculated at the genome level using 1,000 permutations. Only the QTLs with a LOD score significant at greater than 90% genome-wide were retained. QTLs were detected for live or dead skin using the Kruskal Wallis test, regions with a K value > 8 were considered key regions for further analysis (Table 1).

To identify traits that could be mapped, each measured trait was analyzed using the Kruskal Wallis test against each mapped SNP. The Kruskal Wallis test was chosen over interval mapping because it is suitable for small populations. Interval mapping assumes normal distribution within the dataset which is not generally achieved in populations less than 100 individuals. The Kruskal Wallis test generated many QTLs however only findings with a KW score > 7.8 (*p*<0.005; K values significance probability threshold 1 or greater in Table 1; 4 stars or more in Supplementary data 4 and 5) were reported.

## Supplementary Information

**Additional file 1.** MacNee et al-BMC Plants-Supplementary DATA.

**Additional file 2. **MacNee et al. Mapping paper **Supplementary Table 1.** Cross referencing mapped markers to nearby genes matching genes in the literature already associated with pericarp/suberin/cuticle development

**Additional file 3.** CKMEXCK_PS1.1.69.0_K857.

## Data Availability

All data generated or analysed during this study are included in this article and its supplementary information files. The compressed variant call file (634.1 MB) for SNP variants to the published *A. chinensis* genome [reference 17] is available at 10.5281/zenodo.4722054. File name is: ‘CKMEXCK_PS1.1.69.0_K857.vcf.gz’.

## Abbreviations

QTL: Quantitative trait locus
Chr: Chromosome
RAPD: Random Amplified Polymorphic DNA
sp.: species
ABCG: ATP-binding cassette family G
SHN1/WIN1: SHINE1/WAX INDUCER1
ME: *Actinidia melanandra*
CK: *Actinidia chinensis*
GBS: Genotyping By Sequencing
rtGBS: Random tagging genotyping by sequencing
SNP: Single nucleotide polymorphism
MAFs: Minor allele frequencies
LG: Linkage group
Mbp: Mega base pair
bp: base pair
BLASTP: Basic Local Alignment Search Tool comparing protein sequences
MYB: Transcription factor family of proteins that include the conserved MYB DNA-binding domain (in plants characterised by the R2R3-type MYB domain), named after eponymous gene in Avian myeloblastosis virus
PFR: The New Zealand Institute for Plant and Food Research Ltd
