# Supplementary material for: Peridermal fruit skin formation in Actinidia sp. (kiwifruit) is associated with genetic loci controlling russeting and cuticle formation
Source: BMC Plant Biol. 2021 Jul 14;21:334. doi: 10.1186/s12870-021-03025-2 (PMC8278711; doi:10.1186/s12870-021-03025-2)
Supplement: Supplementary file 1 — Additional file 1. MacNee et al-BMC Plants-Supplementary DATA. [file 12870_2021_3025_MOESM1_ESM.docx]

# Title page

# pagSupplementary data for

Peridermal fruit skin formation in *Actinidia* sp. (kiwifruit) is associated with genetic loci controlling russeting and cuticle formation

**Nikolai Macnee^1,2^, Elena Hilario^1^, Jibran Tahir^3^, Alastair Currie^4^, Ben Warren^1^, Ria Rebstock^1^ Ian C. Hallett^1^, David Chagné^3^, Robert J. Schaffer^2,4^, Sean M. Bulley^5*^**

^1^ The New Zealand Institute for Plant & Food Research Ltd (PFR), Priv. Bag 92169, Auckland, 1146, New Zealand.

^2^ School of Biological Science, The University of Auckland, Auckland 1146, New Zealand.

^3^ PFR, Private Bag 11600, Palmerston North 4442, New Zealand.

^4^ PFR, 55 Old Mill Road, RD3, Motueka 7198, New Zealand.

^5^ PFR, 412 No 1 Road RD 2, Te Puke, 3182, New Zealand.

***** Correspondence: Sean.Bulley@plantandfood.co.nz; Tel.: (+64-7-9289796)

**Key of terms**

| Code | Description |
| --- | --- |
| **maxd** | Maximum number of peridermal layers observed |
| **mind** | Minimum number of peridermal layers observed |
| **cov** | Coverage of cuticle over fruit exocarp (0 to 100% scale) |
| **ndl** | Periderm depth; Randomised count of dead cell layers |
| **tri** | Trichome density; 0 (none), 1, 2, 3 (high density) scale |
| **lig** | Lignified trichome; scored 0 (no lignin) or 1 (lignified) |
| **subtri** | Suberised trichomes; scored 0 (no suberisation) or 1 (suberised) trichomes |
| **cuticle** | Cuticle Thickness; 0, 0.5, 1, 1.5, 2.5, 3 scale; score 0 (cuticle not present) to 3 (thick cuticle) |
| **crack** | Microcracking; 0 , 1, 2, 3 scale; score 0 (no microcracks) to 3 (extensive microcracking) |
| **hairy** | Hairyness; 0 (no hairs), 1, 2, 3 (many hairs) scale measured macroscopically |
| **semi** | Partial russet ranging from 20-80% (1) or not semi russet (0) |
| **russet** | Russeting %; percentage of fruit surface covered by periderm |
| **pheno** | Periderm; Epidermal (0) or peridermal (1) |
| **year** | Year of collection; Samples collected during summer of 2016 (0) or 2017 (1) |
| **length** | Mean length (from stem to style) of whole fruit |
| **width** | Mean width / maximum diameter of whole fruit |
| **area** | Mean length x width of whole fruit sampled |

.

**Supplementary data 1**: Additional phenotype scoring

| **maxd** | **mind** | **cov** | **ndl** | **tri** | **lig** | **subtri** | **cuticle** | **crack** | **hairy** | **semi** | **russet** | **pheno** | **year** | **length** | **width** | **area** |
| --- | --- | --- | --- | --- | --- | --- | --- | --- | --- | --- | --- | --- | --- | --- | --- | --- |
| 3 | 0 | 80 | 4 | 1 | 1 | 1 | 0.5 | 1 | 2 | 1 | 30 | 0 | 2017 | 294.3 | 281.7 | 82903.9 |
| 6 | 0 | 50 | 7 | 2 | 1 | 1 | 0.5 | 1 | 1 | 0 | 10 | 0 | 2016 | 232 | 266.5 | 61828 |
| 10 | 0 | 80 | 7 | 1 | 1 | 1 | 1 | 1 | 2 | 1 | 50 | 0 | 2016 | 258.7 | 265.3 | 68632.9 |
| 8 | 0 | 80 | 7 | 1 | 1 | 0 | 1 | 1 | 1 | 1 | 60 | 0 | 2016 | 336 | 296 | 99456 |
| 5 | 0 | 80 | 3 | 1 | 1 | 1 | 0 | 1 | 1 | 1 | 10 | 0 | 2016 | 316 | 284 | 89744 |
| 3 | 0 | 100 | 0 | 1 | 1 | 0 | 2 | 1 | 1 | 0 | 20 | 0 | 2016 | 405 | 327 | 132435 |
| 9 | 0 | 65 | 12 | 1 | 1 | 1 | 1 | 1 | 1 | 1 | 10 | 0 | 2016 | 336.7 | 262 | 88206.7 |
| 6 | 3 | 30 | 25 | 1 | 1 | 1 | 1 | 1 | 1 | 0 | 90 | 1 | 2016 | 313.7 | 253.3 | 79462.2 |
| 4 | 0 | 90 | 5 | 1 | 1 | 1 | 1 | 1 | 2 | 1 | 5 | 0 | 2016 | 238 | 263 | 62594 |
| 5 | 0 | 95 | 1 | 1 | 1 | 1 | 2 | 1 | 2 | 1 | 5 | 0 | 2016 | 281 | 267.3 | 75120.7 |
| 6 | 0 | 10 | 24 | 1 | 1 | 1 | 0 | 1 | 3 | 0 | 95 | 1 | 2016 | 267.7 | 288 | 77088 |
| 4 | 0 | 80 | 2 | 1 | 1 | 1 | 1 | 1 | 1 | 1 | 20 | 0 | 2016 | 344.3 | 274 | 94347.3 |
| 3 | 0 | 90 | 1 | 1 | 1 | 1 | 2 | 1 | 2 | 1 | 40 | 0 | 2016 | 310.7 | 263.7 | 81912.4 |
| 4 | 0 | 60 | 8 | 2 | 0 | 1 | 1 | 1 | 1 | 1 | 30 | 0 | 2016 | 358 | 279.7 | 100120.7 |
| 3 | 0 | 98 | 1 | 1 | 1 | 0 | 2 | 0 | 1 | 0 | 10 | 0 | 2016 | 312 | 285.3 | 89024 |
| 5 | 0 | 80 | 5 | 1 | 1 | 1 | 1 | 1 | 1 | 1 | 30 | 0 | 2016 | 374.7 | 341.3 | 127886.2 |
| 7 | 0 | 80 | 4 | 1 | 1 | 1 | 1 | 1 | 1 | 0 | 10 | 0 | 2016 | 284 | 254.3 | 72230.7 |
| 9 | 0 | 50 | 17 | 1 | 1 | 1 | 1 | 1 | 1 | 1 | 30 | 0 | 2016 | 298 | 255.7 | 76188.7 |
| 4 | 0 | 90 | 3 | 1 | 1 | 1 | 1 | 1 | 1 | 0 | 20 | 0 | 2016 | 370.7 | 347 | 128621.3 |
| 10 | 0 | 50 | 9 | 1 | 1 | 1 | 1 | 1 | 1 | 0 | 20 | 0 | 2016 | 289.7 | 288.7 | 83617.1 |
| 4 | 0 | 70 | 6 | 1 | 1 | 1 | 1 | 1 | 2 | 0 | 10 | 0 | 2016 | 287 | 256 | 73472 |
| 6 | 3 | 0 | 12 | 1 | 1 | 1 | 0 | 1 | 1 | 0 | 90 | 1 | 2016 | 293.7 | 260 | 76353.3 |
| 8 | 4 | 0 | 16 | 1 | 1 | 1 | 0 | 1 | 2 | 0 | 95 | 1 | 2016 | 302.3 | 318.3 | 96242.8 |
| 6 | 0 | 80 | 7 | 1 | 1 | 1 | 1 | 1 | 3 | 0 | 20 | 0 | 2016 | 261.3 | 243.3 | 63591.1 |
| 3 | 0 | 90 | 2 | 1 | 1 | 0 | 2 | 1 | 1 | 0 | 10 | 0 | 2016 | 327.3 | 278.7 | 91216.9 |
| 5 | 0 | 90 | 2 | 1 | 1 | 1 | 2.5 | 1 | 3 | 0 | 20 | 0 | 2016 | 301 | 248.5 | 74798.5 |

**Supplementary data 1**: Additional phenotype scoring continued

| **maxd** | **mind** | **cov** | **ndl** | **tri** | **lig** | **subtri** | **cuticle** | **crack** | **hairy** | **semi** | **russet** | **pheno** | **year** | **length** | **width** | **area** |
| --- | --- | --- | --- | --- | --- | --- | --- | --- | --- | --- | --- | --- | --- | --- | --- | --- |
| 3 | 0 | 75 | 5 | 1 | 1 | 1 | 1 | 1 | 2 | 1 | 30 | 0 | 2016 | 264.7 | 271.7 | 71901.1 |
| 6 | 0 | 80 | 5 | 1 | 1 | 1 | 2 | 1 | 1 | 0 | 10 | 0 | 2016 | 290.3 | 274.3 | 79648.1 |
| 4 | 0 | 80 | 5 | 1 | 1 | 1 | 2 | 1 | 1 | 1 | 5 | 0 | 2016 | 424.3 | 327.3 | 138898.4 |
| 4 | 0 | 80 | 8 | 1 | 1 | 1 | 1 | 1 | 1 | 1 | 5 | 0 | 2016 | 272.7 | 245.3 | 66894.2 |
| 7 | 0 | 60 | 15 | 1 | 1 | 1 | 1 | 1 | 3 | 1 | 5 | 0 | 2016 | 294.7 | 241.7 | 71211.1 |
| 5 | 0 | 90 | 2 | 1 | 1 | 1 | 1 | 1 | 1 | 0 | 10 | 0 | 2016 | 284.7 | 327.3 | 93180.9 |
| 10 | 0 | 60 | 16 | 1 | 1 | 1 | 1 | 1 | 2 | 0 | 10 | 0 | 2016 | 331 | 327 | 108237 |
| 9 | 0 | 80 | 3 | 1 | 1 | 1 | 0.5 | 1 | 1 | 0 | 20 | 0 | 2016 | 309 | 259.7 | 80237 |
| 7 | 0 | 90 | 3 | 1 | 1 | 1 | 1 | 1 | 1 | 0 | 10 | 0 | 2016 | 262 | 251.7 | 65936.7 |
| 5 | 0 | 75 | 3 | 1 | 0 | 0 | 2 | 0 | 1 | 0 | 20 | 0 | 2016 | 219.3 | 258 | 56588 |
| 4 | 0 | 70 | 3 | 1 | 1 | 1 | 0.5 | 2 | 2 | 1 | 5 | 0 | 2016 | 284.3 | 299 | 85015.7 |
| 6 | 0 | 30 | 22 | 1 | 1 | 1 | 1 | 0 | 2 | 0 | 20 | 0 | 2016 | 269.7 | 293 | 79012.3 |
| 7 | 2 | 0 | 31 | 1 | 1 | 1 | 0 | 1 | 1 | 0 | 100 | 1 | 2016 | 398 | 320.3 | 127492.7 |
| 3 | 0 | 30 | 5 | 1 | 1 | 1 | 0.5 | 2 | 3 | 1 | 20 | 0 | 2016 | 319 | 249 | 79431 |
| 1 | 0 | 100 | 0 | 1 | 1 | 0 | 1 | 1 | 1 | 0 | 5 | 0 | 2016 | 260.7 | 251.3 | 65514.2 |
| 3 | 0 | 95 | 0 | 1 | 0 | 1 | 2 | 1 | 1 | 0 | 10 | 0 | 2016 | 350 | 336.7 | 117833.3 |
| 8 | 2 | 0 | 35 | 1 | 1 | 1 | 0 | 1 | 1 | 1 | 90 | 1 | 2016 | 325.7 | 301.7 | 98242.8 |
| 6 | 0 | 90 | 6 | 1 | 0 | 1 | 2 | 1 | 2 | 0 | 5 | 0 | 2016 | 344 | 335.7 | 115469.3 |
| 12 | 0 | 50 | 14 | 1 | 1 | 1 | 1 | 1 | 1 | 0 | 10 | 0 | 2016 | 315.7 | 302.3 | 95436.6 |
| 4 | 0 | 80 | 5 | 1 | 1 | 1 | 1 | 1 | 1 | 0 | 20 | 0 | 2016 | 296 | 262.3 | 77650.7 |
| 8 | 0 | 20 | 10 | 1 | 1 | 0 | 0.5 | 1 | 2 | 1 | 30 | 0 | 2016 | 225.7 | 264 | 59576 |
| 10 | 2 | 0 | 30 | 1 | 1 | 0 | 0 | 1 | 2 | 0 | 100 | 1 | 2016 | 275.7 | 295.3 | 81413.6 |
| 3 | 0 | 90 | 2 | 1 | 1 | 0 | 1 | 1 | 1 | 0 | 10 | 0 | 2016 | 434.7 | 369.7 | 160681.8 |
| 5 | 0 | 20 | 22 | 1 | 1 | 0 | 0.5 | 1 | 2 | 1 | 40 | 0 | 2016 | 263.7 | 281 | 74090.3 |
| 3 | 0 | 70 | 8 | 1 | 1 | 1 | 1 | 1 | 1 | 0 | 10 | 0 | 2016 | 379 | 240 | 90960 |
| 4 | 0 | 60 | 9 | 1 | 1 | 1 | 0.5 | 1 | 1 | 0 | 20 | 0 | 2016 | 379 | 350 | 132650 |
| 4 | 0 | 20 | 8 | 1 | 1 | 1 | 0.5 | 1 | 1 | 0 | 10 | 0 | 2016 | 320 | 375 | 120000 |
| 2 | 0 | 100 | 0 | 1 | 1 | 0 | 2 | 1 | 3 | 0 | 10 | 0 | 2016 | 308.3 | 269.7 | 83147.2 |

**Supplementary data 1**: Additional phenotype scoring continued

| **maxd** | **mind** | **cov** | **ndl** | **tri** | **lig** | **subtri** | **cuticle** | **crack** | **hairy** | **semi** | **russet** | **pheno** | **year** | **length** | **width** | **area** |
| --- | --- | --- | --- | --- | --- | --- | --- | --- | --- | --- | --- | --- | --- | --- | --- | --- |
| 4 | 0 | 70 | 5 | 1 | 1 | 1 | 0.5 | 1 | 1 | 0 | 5 | 0 | 2016 | 172.7 | 227.5 | 39281.7 |
| 5 | 0 | 20 | 23 | 1 | 1 | 1 | 1 | 1 | 1 | 1 | 60 | 0 | 2016 | 399.3 | 323 | 128984.7 |
| 4 | 0 | 50 | 7 | 1 | 1 | 1 | 1 | 1 | 1 | 1 | 50 | 0 | 2017 | 266.7 | 215.3 | 57407.4 |
| 4 | 0 | 20 | 26 | 1 | 1 | 1 | 1 | 1 | 2 | 1 | 50 | 0 | 2016 | 391 | 282.3 | 110392.3 |
| 8 | 2 | 0 | 28 | 1 | 1 | 1 | 0 | 1 | 1 | 0 | 60 | 1 | 2017 | 437.2 | 297.4 | 130032.9 |
| 7 | 0 | 90 | 5 | 1 | 1 | 1 | 1 | 1 | 2 | 0 | 5 | 0 | 2017 | 412.8 | 298.7 | 123316.9 |
| 6 | 0 | 60 | 5 | 1 | 1 | 1 | 1 | 1 | 3 | 1 | 60 | 0 | 2017 | 211.5 | 269.2 | 56952.7 |
| 6 | 0 | 50 | 12 | 1 | 1 | 1 | 0.5 | 1 | 2 | 0 | 50 | 0 | 2017 | 234.6 | 275.6 | 64669.6 |
| 8 | 3 | 0 | 44 | 1 | 1 | 1 | 0 | 1 | 1 | 1 | 80 | 1 | 2017 | 339.7 | 355.1 | 120652.5 |
| 9 | 0 | 60 | 18 | 1 | 1 | 1 | 1 | 1 | 1 | 0 | 20 | 0 | 2017 | 267.9 | 337.2 | 90346.8 |
| 4 | 0 | 90 | 3 | 1 | 1 | 1 | 2 | 1 | 1 | 1 | 40 | 0 | 2016 | 201.3 | 235 | 47313.3 |
| 9 | 0 | 20 | 18 | 2 | 1 | 1 | 0.5 | 1 | 1 | 0 | 100 | 1 | 2017 | 307.7 | 250 | 76923.1 |
| 5 | 0 | 90 | 2 | 1 | 1 | 1 | 2 | 1 | 1 | 0 | 10 | 0 | 2016 | 219 | 227.7 | 49859 |
| 6 | 0 | 80 | 2 | 1 | 1 | 1 | 1 | 1 | 1 | 0 | 5 | 0 | 2016 | 240.7 | 272 | 65461.3 |
| 4 | 0 | 90 | 2 | 1 | 1 | 1 | 1 | 0 | 1 | 0 | 20 | 0 | 2016 | 236 | 271.7 | 64113.3 |
| 4 | 0 | 95 | 0 | 1 | 1 | 1 | 1 | 1 | 1 | 1 | 40 | 0 | 2016 | 360.3 | 281 | 101253.7 |

**Supplementary data 2**: correlation table of phenotypic traits (Pearson correlation)


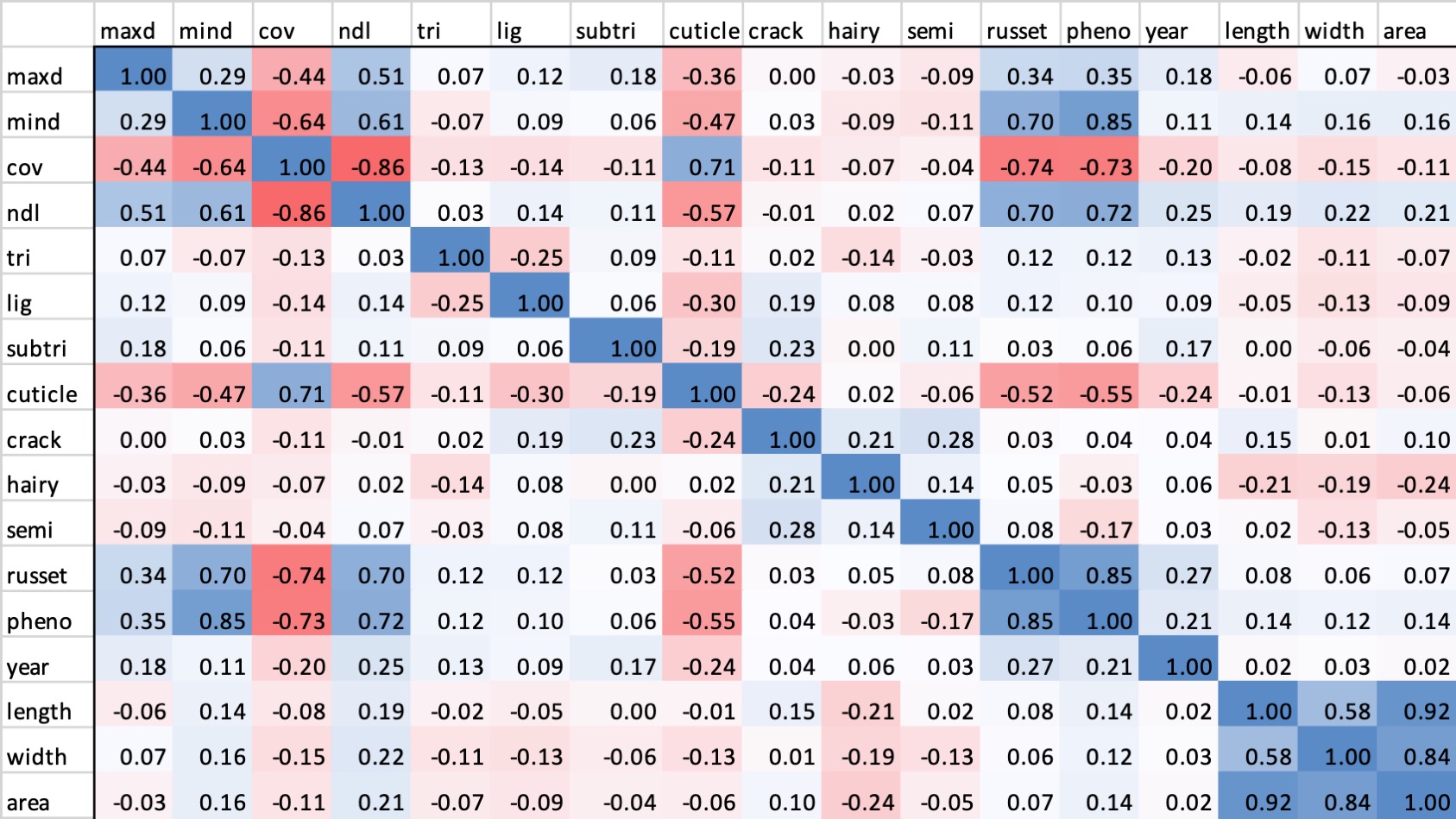


**Supplementary data 3A**. Mapping statistics, Q-Q Plot above, Self-relatedness score below


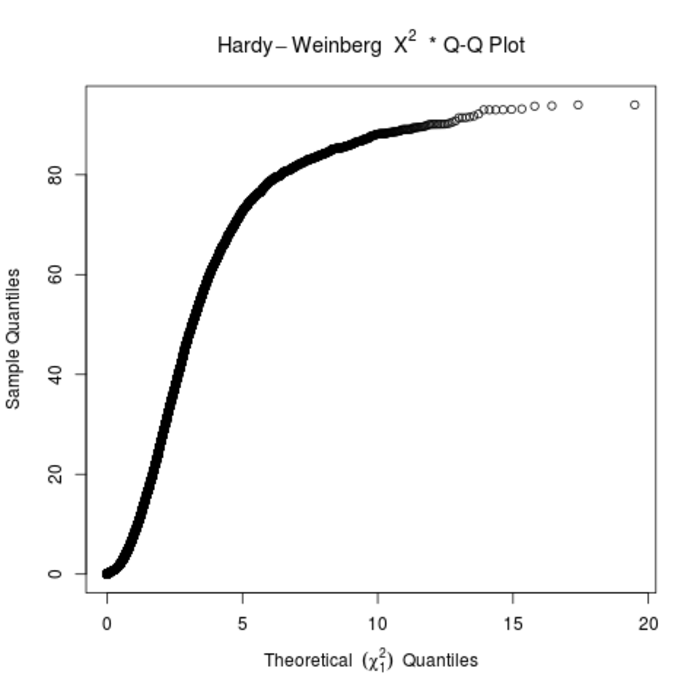


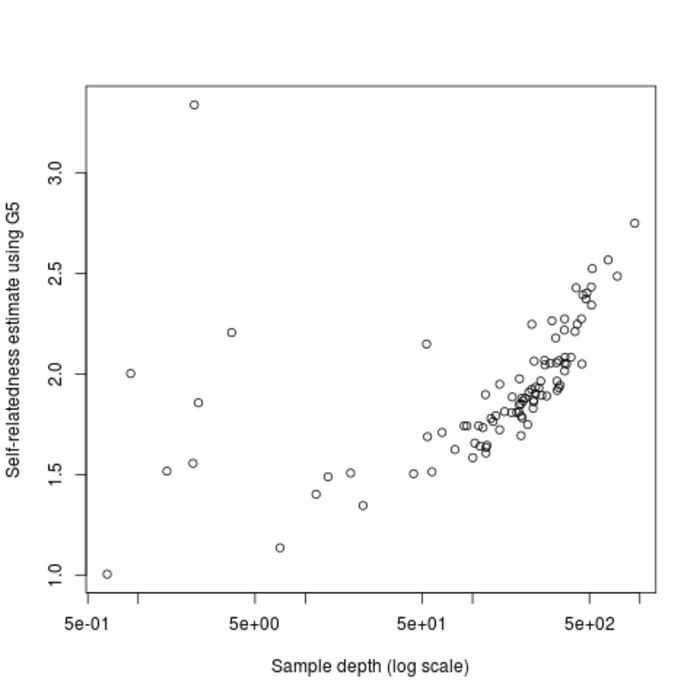


**Supplementary data 3B**. Mapping statistics, SNP call rate above, mean sample depth below


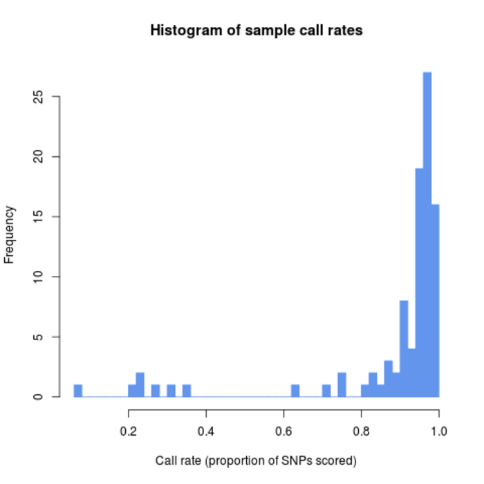


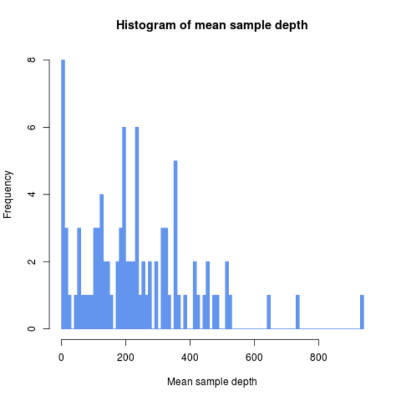


**Supplementary data 3C**. Mapping statistics, SNP call rate


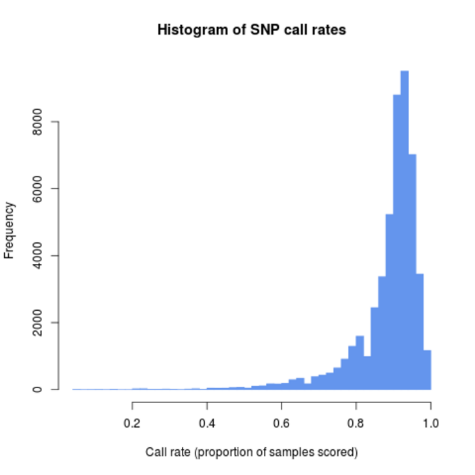


**Supplementary data 3D**. Mapping statistics, Principal component analysis above, MDS coordinate below


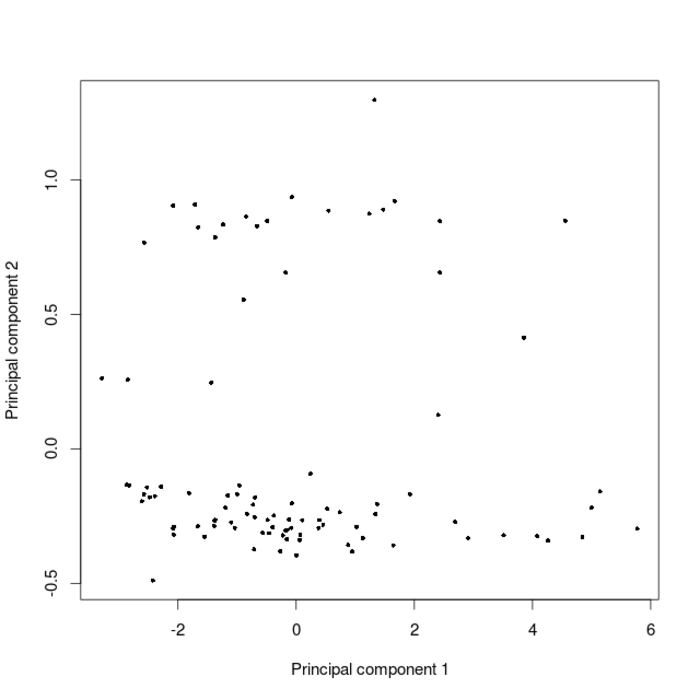


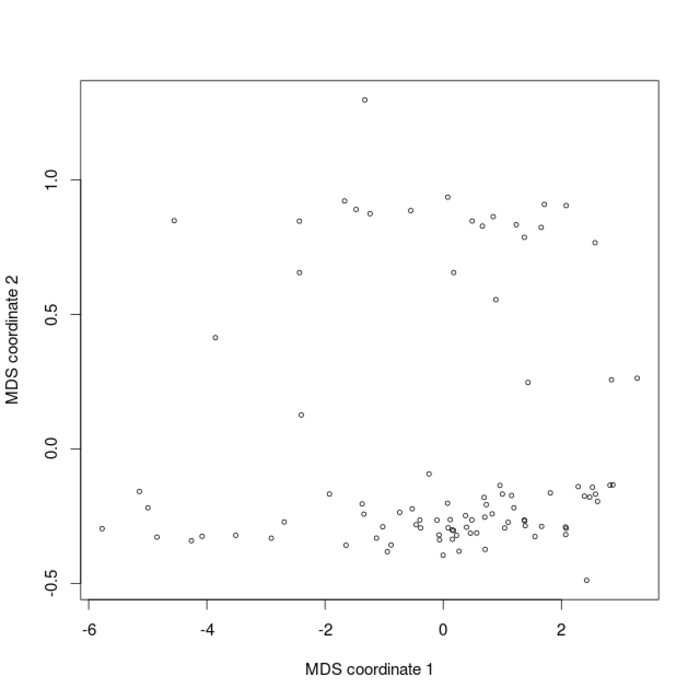


**Supplementary data 3E**. Mapping statistics, Hardy Weinberg likelihood ratio above and minor allele frequency below


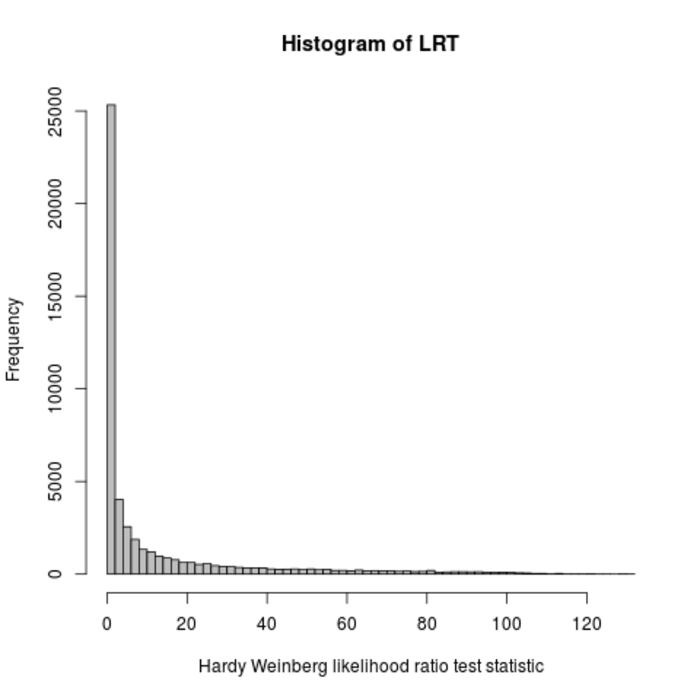


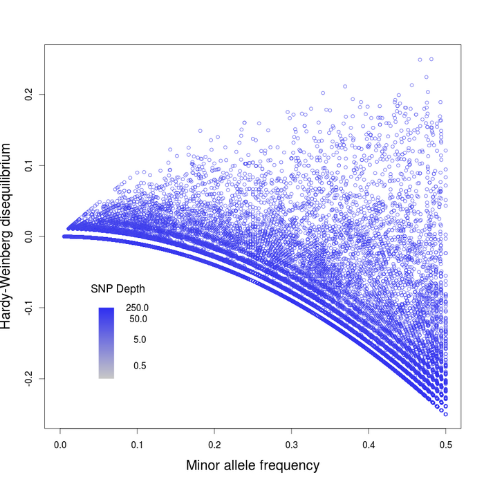


**Supplementary data 3F**. Mapping statistics, NSP depth up to 580 above, self related estimates using G4 below


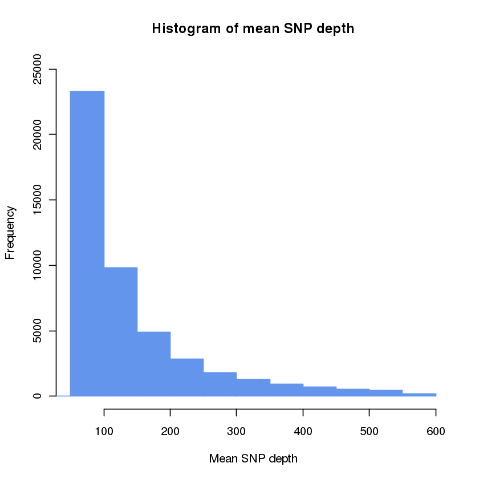


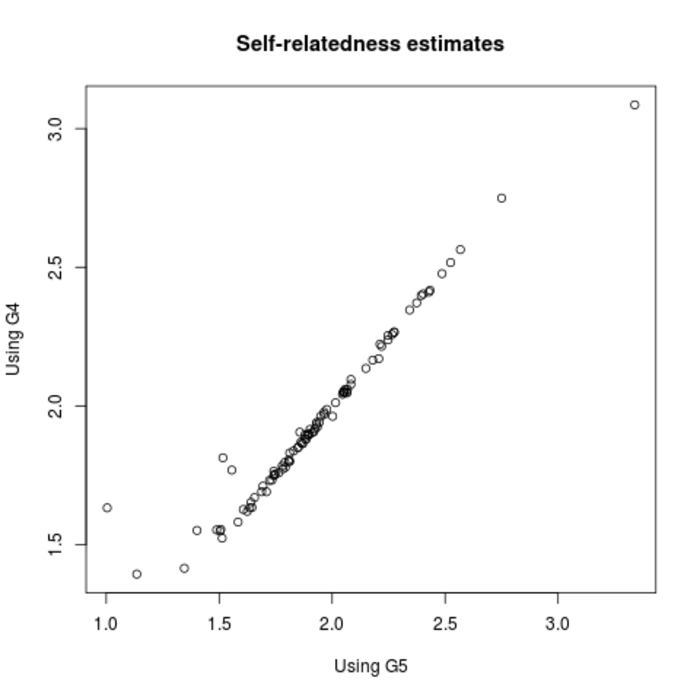


**Supplementary data 3G**. Mapping statistics, allel frequency counts above, minor allele frequency distrubtion below


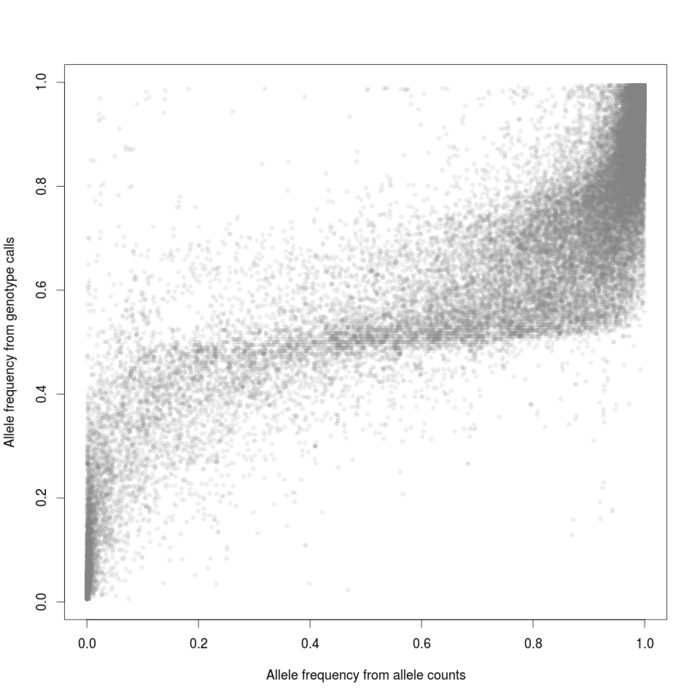


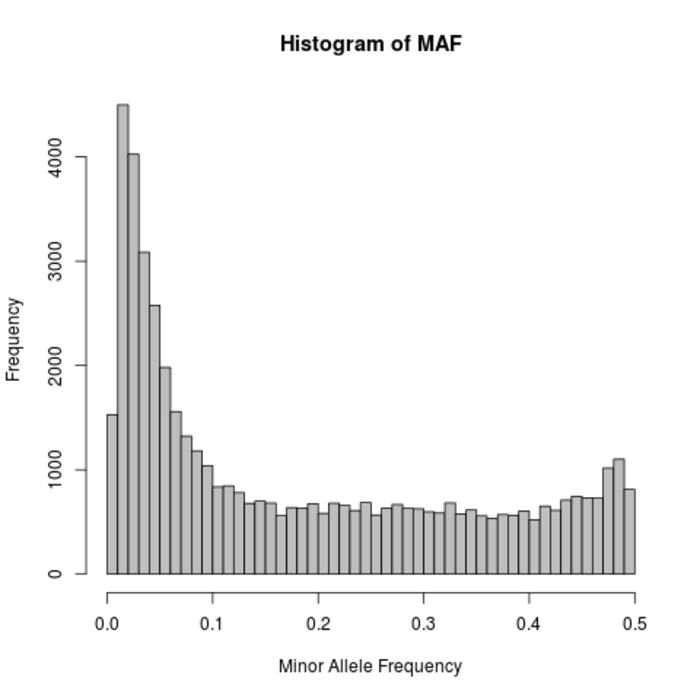


**Supplementary data 3.H**. Mapping statistics, sample depth vs call rate above, SNP Depth below


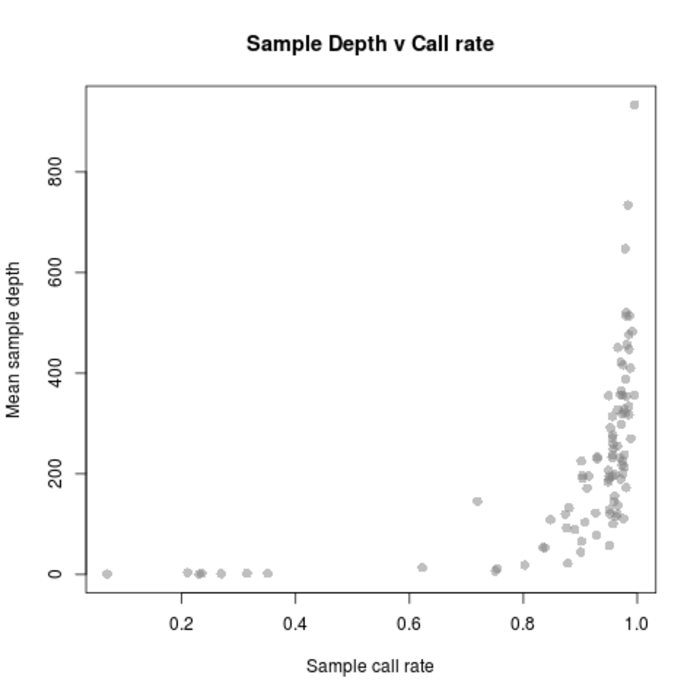


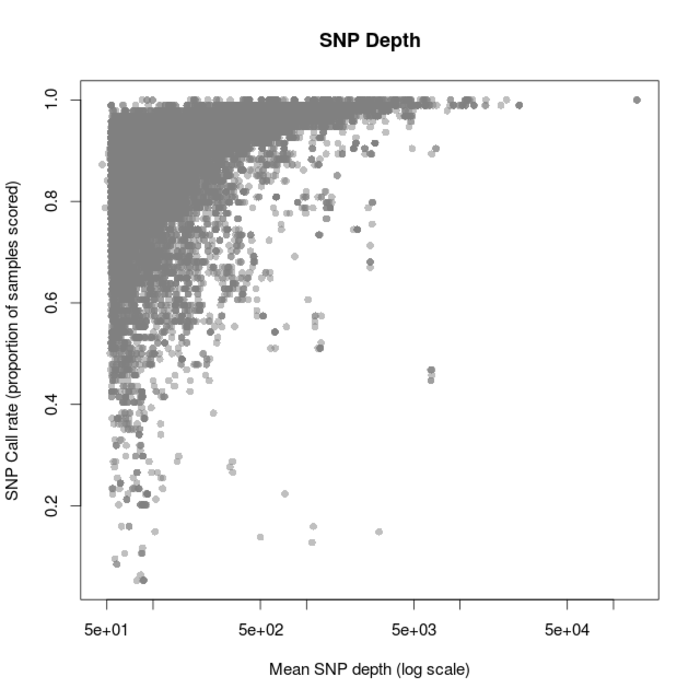


**Supplementary data 4:** A Russet/ Cuticle related mapping results

| **Map** | **Trait** | **Nr** | **Group** | **Position** | **Locus** | **K*** | **Signif.** | **Nr inf.** | **Meanrank-ll** | **Mean-ll** | **Nr-ll** | **Meanrank-lm** | **Mean-lm** | **Nr-lm** |
| --- | --- | --- | --- | --- | --- | --- | --- | --- | --- | --- | --- | --- | --- | --- |
| Mother | Periderm | 1349 | 154 | 1.515 | CHR3_13504746 | 10.262 | **** | 67 | 40.7 | 0.333333 | 21 | 31 | 0.043478 | 46 |
| Mother | Periderm | 1350 | 154 | 3.031 | CHR3_13504758 | 9.379 | **** | 67 | 40.2 | 0.318182 | 22 | 31 | 0.044444 | 45 |
| Mother | Periderm | 1351 | 154 | 3.031 | CHR3_13504785 | 9.379 | **** | 67 | 40.2 | 0.318182 | 22 | 31 | 0.044444 | 45 |
| Mother | Periderm | 992 | 102 | 1.563 | CHR19_12859243 | 8.804 | **** | 65 | 37.8 | 0.285714 | 28 | 29.4 | 0.027027 | 37 |
| Mother | Periderm | 995 | 102 | 4.688 | CHR19_12859116 | 8.804 | **** | 65 | 37.8 | 0.285714 | 28 | 29.4 | 0.027027 | 37 |
| Mother | Periderm | 993 | 102 | 3.125 | CHR19_12859098 | 8.159 | **** | 65 | 37.5 | 0.275862 | 29 | 29.4 | 0.027778 | 36 |
| Mother | Periderm | 994 | 102 | 3.125 | CHR19_12859164 | 8.159 | **** | 65 | 37.5 | 0.275862 | 29 | 29.4 | 0.027778 | 36 |
| Mother | Russet | 998 | 102 | 27.729 | CHR19_12859223 | 19.753 | ******* | 66 | 41.6 | 41.0976 | 41 | 20.2 | 12.6 | 25 |
| Mother | Russet | 996 | 102 | 27.729 | CHR19_12859101 | 19.116 | ******* | 65 | 41 | 41.125 | 40 | 20.2 | 12.6 | 25 |
| Mother | Russet | 997 | 102 | 27.729 | CHR19_12859121 | 19.116 | ******* | 65 | 41 | 41.125 | 40 | 20.2 | 12.6 | 25 |
| Mother | Russet | 993 | 102 | 3.125 | CHR19_12859098 | 8.96 | **** | 65 | 40.7 | 43.2759 | 29 | 26.8 | 19.5833 | 36 |
| Mother | Russet | 994 | 102 | 3.125 | CHR19_12859164 | 8.96 | **** | 65 | 40.7 | 43.2759 | 29 | 26.8 | 19.5833 | 36 |
| Mother | Russet | 992 | 102 | 1.563 | CHR19_12859243 | 8.949 | **** | 65 | 40.9 | 44.1071 | 28 | 27 | 19.5946 | 37 |
| Mother | Russet | 995 | 102 | 4.688 | CHR19_12859116 | 10.153 | **** | 65 | 41.5 | 44.4643 | 28 | 26.6 | 19.3243 | 37 |
| Mother | Russet | 988 | 101 | 61.805 | CHR19_12109055 | 13.445 | ****** | 65 | 26.7 | 21.3095 | 42 | 44.4 | 45.4348 | 23 |
| Mother | Russet | 989 | 101 | 63.367 | CHR19_12109042 | 11.055 | ***** | 65 | 27.5 | 22.907 | 43 | 43.7 | 43.4091 | 22 |
| Mother | Russet | 987 | 101 | 51.6 | CHR19_12109040 | 9.506 | **** | 65 | 27.4 | 23.125 | 40 | 42 | 40.6 | 25 |
| Mother | Russet | 816 | 84 | 40.532 | CHR23_11768369 | 10.876 | ***** | 61 | 38.5 | 40 | 30 | 23.7 | 19.3548 | 31 |
| Mother | Russet | 812 | 84 | 28.013 | CHR19_15672217 | 9.688 | **** | 68 | 41.6 | 38.8571 | 35 | 26.9 | 19.697 | 33 |
| Mother | Russet | 814 | 84 | 38.837 | CHR23_11768507 | 8.593 | **** | 61 | 37.5 | 38.871 | 31 | 24.3 | 19.8333 | 30 |
| Mother | Russet | 815 | 84 | 38.837 | CHR23_11768508 | 8.593 | **** | 61 | 37.5 | 38.871 | 31 | 24.3 | 19.8333 | 30 |
| Mother | Russet | 810 | 84 | 0 | CHR19_14584990 | 8.56 | **** | 66 | 26.1 | 20 | 30 | 39.7 | 37.7778 | 36 |
| Mother | Russet | 817 | 84 | 58.434 | CHR19_15784654 | 8.34 | **** | 64 | 26.9 | 21.6216 | 37 | 40.2 | 39.2593 | 27 |
| Mother | Russet | 491 | 54 | 100.187 | CHR5_6468572 | 8.24 | **** | 66 | 40.6 | 40 | 31 | 27.2 | 21.1429 | 35 |
| Mother | Russet | 492 | 54 | 100.187 | CHR5_6468577 | 8.24 | **** | 66 | 40.6 | 40 | 31 | 27.2 | 21.1429 | 35 |
| Mother | Russet | 493 | 54 | 100.187 | CHR5_6468591 | 8.24 | **** | 66 | 40.6 | 40 | 31 | 27.2 | 21.1429 | 35 |

**Supplementary data 4:** A Russet/ Cuticle related mapping results continued

| **Map** | **Trait** | **Nr** | **Group** | **Position** | **Locus** | **K*** | **Signif.** | **Nr inf.** | **Meanrank-ll** | **Mean-ll** | **Nr-ll** | **Meanrank-lm** | **Mean-lm** | **Nr-lm** |
| --- | --- | --- | --- | --- | --- | --- | --- | --- | --- | --- | --- | --- | --- | --- |
| Mother | Perderm L | 714 | 76 | 18.735 | CHR11_2562857 | 11.757 | ***** | 68 | 26.1 | 5.18182 | 33 | 42.5 | 13.5714 | 35 |
| Mother | Perderm L | 715 | 76 | 18.735 | CHR11_2562874 | 11.757 | ***** | 68 | 26.1 | 5.18182 | 33 | 42.5 | 13.5714 | 35 |
| Mother | Perderm L | 716 | 76 | 21.766 | CHR11_2562887 | 8.123 | **** | 68 | 27.5 | 5.54545 | 33 | 41.1 | 13.2286 | 35 |
| Mother | Perderm L | 384 | 42 | 4.616 | CHR3_19907609 | 8.191 | **** | 66 | 38.4 | 10.9302 | 43 | 24.3 | 6.82609 | 23 |
| Mother | Perderm L | 385 | 42 | 4.616 | CHR3_19907622 | 8.191 | **** | 66 | 38.4 | 10.9302 | 43 | 24.3 | 6.82609 | 23 |
| Mother | Perderm L | 386 | 42 | 4.616 | CHR3_19907648 | 8.191 | **** | 66 | 38.4 | 10.9302 | 43 | 24.3 | 6.82609 | 23 |
| Mother | Perderm L | 387 | 42 | 4.616 | CHR3_19907792 | 8.191 | **** | 66 | 38.4 | 10.9302 | 43 | 24.3 | 6.82609 | 23 |
| Mother | Perderm L | 383 | 42 | 3.077 | CHR3_19907864 | 11.04 | ***** | 66 | 39 | 11.4773 | 44 | 22.4 | 5.54545 | 22 |
| Mother | Perderm L | 382 | 42 | 1.539 | CHR3_19907869 | 10.022 | **** | 66 | 39 | 11.5581 | 43 | 23.3 | 5.65217 | 23 |
| Mother | Perderm L | 134 | 16 | 0 | CHR9_13494968 | 9.12 | **** | 68 | 43.7 | 13.9231 | 26 | 28.8 | 6.80952 | 42 |
| Father | periderm | 306 | 39 | 0 | CHR15_10394929 | 9.993 | **** | 61 | 35.4 | 0.275862 | 29 | 27 | 0 | 32 |
| Father | periderm | 595 | 85 | 13.772 | CHR15_13136758 | 8.535 | **** | 64 | 37.1 | 0.285714 | 28 | 28.9 | 0.027778 | 36 |
| Father | periderm | 596 | 85 | 13.772 | CHR15_13136787 | 8.535 | **** | 64 | 37.1 | 0.285714 | 28 | 28.9 | 0.027778 | 36 |
| Father | periderm | 597 | 85 | 13.772 | CHR15_13136869 | 8.535 | **** | 64 | 37.1 | 0.285714 | 28 | 28.9 | 0.027778 | 36 |
| Father | periderm | 625 | 1 | 17.108 | CHR24_12647436 | 9.974 | **** | 65 | 37.4 | 0.272727 | 33 | 28.5 | 0 | 32 |
| Father | Perderm L | 625 | 1 | 17.108 | CHR24_12647436 | 10.884 | ***** | 65 | 40.6 | 13.5758 | 33 | 25.2 | 5.3125 | 32 |
| Father | Perderm L | 624 | 1 | 0 | CHR24_13092976 | 9.073 | **** | 66 | 42.6 | 12.32 | 25 | 28 | 6.82927 | 41 |
| Father | Perderm L | 594 | 85 | 0 | CHR15_14020022 | 9.51 | **** | 51 | 32.8 | 13.4583 | 24 | 20 | 6.51852 | 27 |
| Father | Semirusset | 463 | 61 | 9.143 | CHR18_4801983 | 11.744 | ***** | 69 | 46.4 | 0.736842 | 19 | 30.7 | 0.28 | 50 |
| Father | Semirusset | 627 | 1 | 41.82 | CHR24_9523224 | 11.279 | ***** | 62 | 26 | 0.194444 | 36 | 39.1 | 0.615385 | 26 |
| Father | Semirusset | 453 | 60 | 0 | CHR8_4050322 | 10.103 | **** | 62 | 36.7 | 0.555556 | 36 | 24.3 | 0.153846 | 26 |
| Father | Semirusset | 560 | 78 | 23.147 | CHR4_1195790 | 9.085 | **** | 66 | 38.2 | 0.536585 | 41 | 25.8 | 0.16 | 25 |
| Father | Semirusset | 561 | 78 | 23.147 | CHR4_1195803 | 9.085 | **** | 66 | 38.2 | 0.536585 | 41 | 25.8 | 0.16 | 25 |
| Father | Russet | 609 | 88 | 0 | CHR21_16714503 | 9.648 | **** | 64 | 41.7 | 39.5833 | 24 | 27 | 20.375 | 40 |
|  |  |  |  |  |  |  |  |  |  |  |  |  |  |  |

**Supplementary data 4:** A Russet/ Cuticle related mapping results continued

| **Map** | **Trait** | **Nr** | **Group** | **Position** | **Locus** | **K*** | **Signif.** | **Nr inf.** | **Meanrank-ll** | **Mean-ll** | **Nr-ll** | **Meanrank-lm** | **Mean-lm** | **Nr-lm** |
| --- | --- | --- | --- | --- | --- | --- | --- | --- | --- | --- | --- | --- | --- | --- |
| Mother | Cuticle T | 714 | 76 | 18.735 | CHR11_2562857 | 11.323 | ***** | 68 | 42.2 | 1.24242 | 33 | 27.3 | 0.757143 | 35 |
| Mother | Cuticle T | 715 | 76 | 18.735 | CHR11_2562874 | 11.323 | ***** | 68 | 42.2 | 1.24242 | 33 | 27.3 | 0.757143 | 35 |
| Mother | Cuticle T | 716 | 76 | 21.766 | CHR11_2562887 | 9.317 | **** | 68 | 41.5 | 1.21212 | 33 | 27.9 | 0.785714 | 35 |
| Mother | Cuticle T | 717 | 76 | 42.462 | CHR11_2831689 | 9.383 | **** | 63 | 37.2 | 1.15385 | 39 | 23.6 | 0.6875 | 24 |
| Mother | Cuticle T | 721 | 76 | 48.791 | CHR11_2831875 | 8.051 | **** | 64 | 37.3 | 1.1625 | 40 | 24.5 | 0.708333 | 24 |
| Mother | Cuticle T | 1137 | 120 | 12.301 | CHR25_6351221 | 9.134 | **** | 62 | 42.8 | 1.4 | 15 | 27.9 | 0.840426 | 47 |
| Mother | Cuticle T | 1143 | 120 | 14.128 | CHR25_6351234 | 9.499 | **** | 63 | 44.3 | 1.42857 | 14 | 28.5 | 0.826531 | 49 |
| Mother | Cuticle T | 1144 | 120 | 14.128 | CHR25_6351264 | 9.499 | **** | 63 | 44.3 | 1.42857 | 14 | 28.5 | 0.826531 | 49 |
| Mother | Cuticle T | 1138 | 120 | 14.128 | CHR25_6351327 | 9.499 | **** | 63 | 44.3 | 1.42857 | 14 | 28.5 | 0.826531 | 49 |
| Mother | Cuticle T | 1139 | 120 | 14.128 | CHR25_6351330 | 9.499 | **** | 63 | 44.3 | 1.42857 | 14 | 28.5 | 0.826531 | 49 |
| Mother | Cuticle T | 1140 | 120 | 14.128 | CHR25_6351333 | 9.499 | **** | 63 | 44.3 | 1.42857 | 14 | 28.5 | 0.826531 | 49 |
| Mother | Cuticle T | 1141 | 120 | 14.128 | CHR25_6351338 | 9.499 | **** | 63 | 44.3 | 1.42857 | 14 | 28.5 | 0.826531 | 49 |
| Mother | Cuticle T | 1145 | 120 | 14.128 | CHR25_6351353 | 9.499 | **** | 63 | 44.3 | 1.42857 | 14 | 28.5 | 0.826531 | 49 |
| Mother | Cuticle T | 1142 | 120 | 14.128 | CHR25_6351361 | 9.499 | **** | 63 | 44.3 | 1.42857 | 14 | 28.5 | 0.826531 | 49 |
| Mother | Cuticle T | 387 | 42 | 4.616 | CHR3_19907792 | 8.513 | **** | 66 | 28.5 | 56.1628 | 43 | 42.8 | 74.4783 | 23 |
| Mother | Cuticle T | 747 | 79 | 0 | CHR25_15529996 | 8.178 | **** | 66 | 39.6 | 71.1944 | 36 | 26.2 | 52.6667 | 30 |
| Father | Cuticle T | 625 | 1 | 17.108 | CHR24_12647436 | 11.529 | ***** | 65 | 25.2 | 47.2727 | 33 | 41 | 77.9062 | 32 |
| Father | Cuticle T | 624 | 1 | 0 | CHR24_13092976 | 8.59 | **** | 66 | 24.7 | 48.8 | 25 | 38.9 | 73.4878 | 41 |
| Father | Cuticle T | 276 | 35 | 0 | CHR26_2817564 | 8.008 | **** | 66 | 38.2 | 1.17857 | 42 | 25.3 | 0.729167 | 24 |
| Father | Cuticle T | 574 | 81 | 0 | CHR26_9237551 | 8.217 | **** | 66 | 36.6 | 1.05263 | 38 | 29.3 | 0.821429 | 28 |
| Father | Cuticle C | 40 | 8 | 0 | CHR20_13600424 | 7.92 | **** | 66 | 27.5 | 0.764706 | 17 | 35.6 | 1.02041 | 49 |
|  |  |  |  |  |  |  |  |  |  |  |  |  |  |  |

**Supplementary data 5.** Mapping results for additional phenotypes

| **Map** | **Trait** | **Nr** | **Group** | **Position** | **Locus** | **K*** | **Signif.** | **Nr inf.** | **Meanrank-ll** | **Mean-ll** | **Nr-ll** | **Meanrank-lm** | **Mean-lm** | **Nr-lm** |
| --- | --- | --- | --- | --- | --- | --- | --- | --- | --- | --- | --- | --- | --- | --- |
| Mother | ligtri | 252 | 29 | 70.137 | CHR2_7177352 | 8.287 | **** | 63 | 27.6 | 0.809524 | 21 | 34.2 | 1.02381 | 42 |
| Mother | ligtri | 253 | 29 | 70.137 | CHR2_7177196 | 7.988 | **** | 64 | 28.3 | 0.818182 | 22 | 34.7 | 1.02381 | 42 |
| Mother | subtri | 159 | 19 | 0 | CHR23_13822945 | 9.854 | **** | 63 | 27 | 0.666667 | 30 | 36.5 | 0.969697 | 33 |
| Mother | subtri | 741 | 78 | 32.81 | CHR25_17557032 | 8.998 | **** | 65 | 28.5 | 0.676471 | 34 | 38 | 0.967742 | 31 |
| Mother | subtri | 1120 | 117 | 46.581 | CHR1_16905719 | 8.023 | **** | 64 | 37.4 | 0.965517 | 29 | 28.4 | 0.685714 | 35 |
| Mother | hairy | 505 | 55 | 73.513 | CHR5_16634767 | 10.689 | **** | 69 | 28.5 | 1.22857 | 35 | 41.7 | 1.67647 | 34 |
| Mother | hairy | 506 | 55 | 73.513 | CHR5_16634800 | 10.689 | **** | 69 | 28.5 | 1.22857 | 35 | 41.7 | 1.67647 | 34 |
| Mother | hairy | 507 | 55 | 73.513 | CHR5_16634807 | 10.689 | **** | 69 | 28.5 | 1.22857 | 35 | 41.7 | 1.67647 | 34 |
| Mother | hairy | 508 | 55 | 73.513 | CHR5_16634687 | 10.173 | **** | 68 | 28 | 1.23529 | 34 | 41 | 1.67647 | 34 |
| Mother | hairy | 509 | 55 | 73.513 | CHR5_16634707 | 10.173 | **** | 68 | 28 | 1.23529 | 34 | 41 | 1.67647 | 34 |
| Mother | hairy | 1189 | 127 | 21.147 | CHR9_10766594 | 7.924 | **** | 64 | 39.5 | 1.76 | 25 | 28 | 1.30769 | 39 |
| Father | subtri | 284 | 36 | 24.961 | CHR6_2342966 | 9.1 | **** | 66 | 37.7 | 0.945946 | 37 | 28.1 | 0.655172 | 29 |
| Father | subtri | 494 | 66 | 54.822 | CHR10_9862892 | 8.425 | **** | 63 | 35.6 | 0.923077 | 39 | 26.2 | 0.625 | 24 |
| Father | subtri | 289 | 36 | 35.735 | CHR6_1576777 | 8.359 | **** | 66 | 37.7 | 0.944444 | 36 | 28.5 | 0.666667 | 30 |
| Father | subtri | 290 | 36 | 38.83 | CHR6_1576775 | 8.359 | **** | 66 | 37.7 | 0.944444 | 36 | 28.5 | 0.666667 | 30 |
| Father | subtri | 531 | 73 | 22.661 | CHR5_12939220 | 8.359 | **** | 66 | 28.5 | 0.666667 | 30 | 37.7 | 0.944444 | 36 |
| Father | subtri | 286 | 36 | 32.623 | CHR6_1576949 | 7.957 | **** | 67 | 38.1 | 0.944444 | 36 | 29.2 | 0.677419 | 31 |
| Father | subtri | 287 | 36 | 32.623 | CHR6_1576999 | 7.957 | **** | 67 | 38.1 | 0.944444 | 36 | 29.2 | 0.677419 | 31 |
| Father | subtri | 288 | 36 | 32.623 | CHR6_1577006 | 7.957 | **** | 67 | 38.1 | 0.944444 | 36 | 29.2 | 0.677419 | 31 |
| Father | hairy | 516 | 70 | 107.85 | CHR29_3032033 | 12.883 | ****** | 65 | 29.4 | 1.34615 | 52 | 47.4 | 2 | 13 |
| Father | hairy | 83 | 14 | 37.454 | CHR9_10513333 | 11.193 | ***** | 65 | 27.7 | 1.275 | 40 | 41.5 | 1.8 | 25 |

**Supplementary data 5.** Mapping results for additional phenotypes continued

| **Map** | **Trait** | **Nr** | **Group** | **Position** | **Locus** | **K*** | **Signif.** | **Nr inf.** | **Meanrank-ll** | **Mean-ll** | **Nr-ll** | **Meanrank-lm** | **Mean-lm** | **Nr-lm** |
| --- | --- | --- | --- | --- | --- | --- | --- | --- | --- | --- | --- | --- | --- | --- |
| Father | hairy | 82 | 14 | 21.447 | CHR9_9640990 | 9.004 | **** | 69 | 29.2 | 1.25 | 36 | 41.4 | 1.66667 | 33 |
| Father | hairy | 362 | 47 | 0 | CHR28_10395294 | 8.538 | **** | 68 | 28.4 | 1.21212 | 33 | 40.2 | 1.68571 | 35 |
| Mother | area | 1063 | 110 | 0 | CHR7_18832417 | 9.096 | **** | 65 | 37.9 | 86068.8 | 44 | 22.8 | 58424.9 | 21 |
| Father | area | 193 | 25 | 54.631 | CHR19_9554828 | 8.338 | **** | 66 | 39.5 | 87415.8 | 37 | 25.8 | 64023.4 | 29 |
| Father | area | 194 | 25 | 54.631 | CHR19_9554844 | 8.338 | **** | 66 | 39.5 | 87415.8 | 37 | 25.8 | 64023.4 | 29 |
| Father | length | 514 | 70 | 74.24 | CHR29_3724853 | 10.025 | **** | 65 | 39.5 | 302.714 | 37 | 24.5 | 223.81 | 28 |
| Father | length | 603 | 86 | 80.742 | CHR11_14564250 | 8.371 | **** | 59 | 35.7 | 293.207 | 33 | 22.7 | 216.55 | 26 |
| Mother | hairy | 507 | 55 | 73.513 | CHR5_16634807 | 10.689 | **** | 69 | 28.5 | 1.22857 | 35 | 41.7 | 1.67647 | 34 |

**Supplementary data 6.** Linkage maps (Paternal)

**
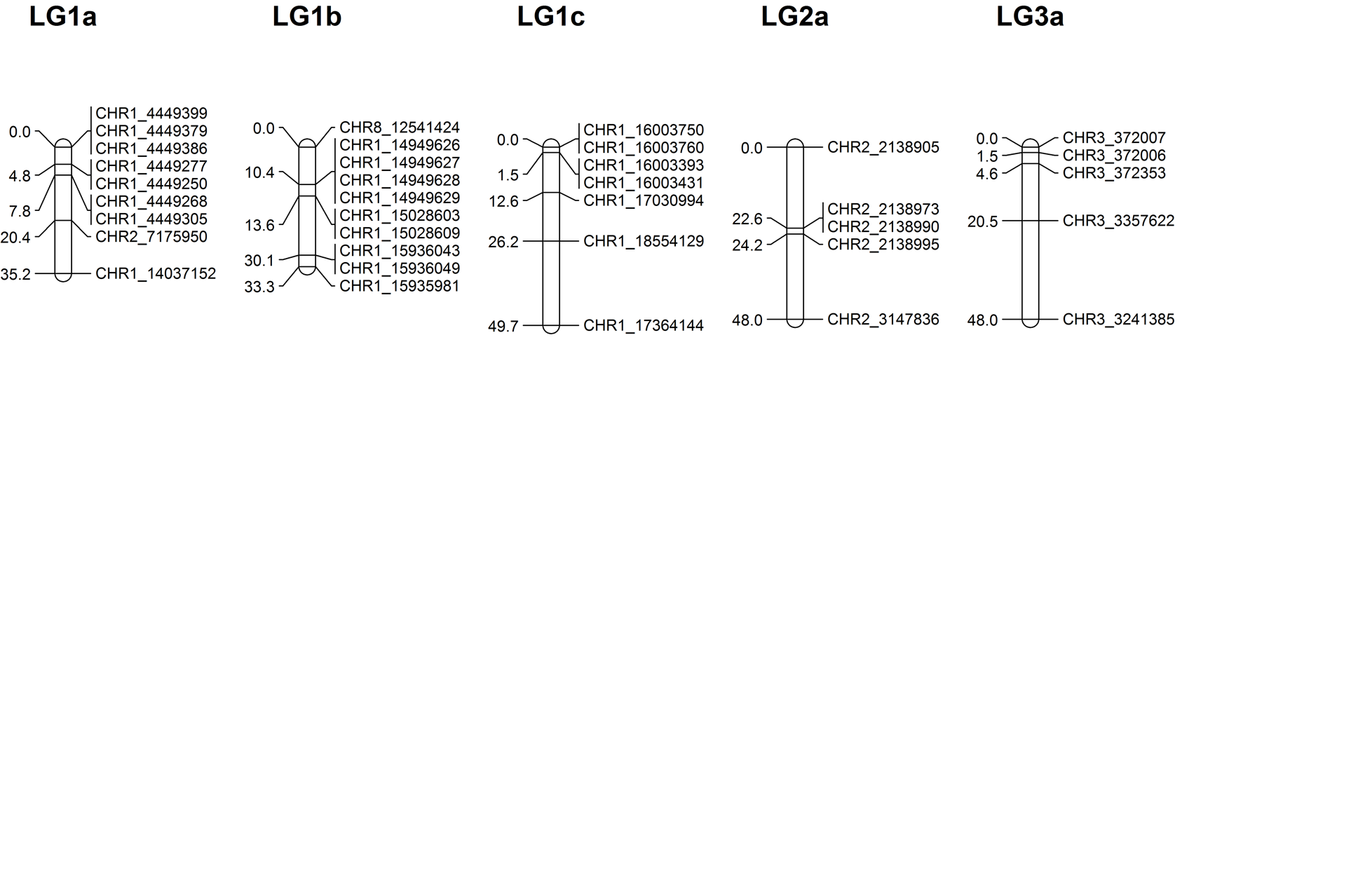
**

**
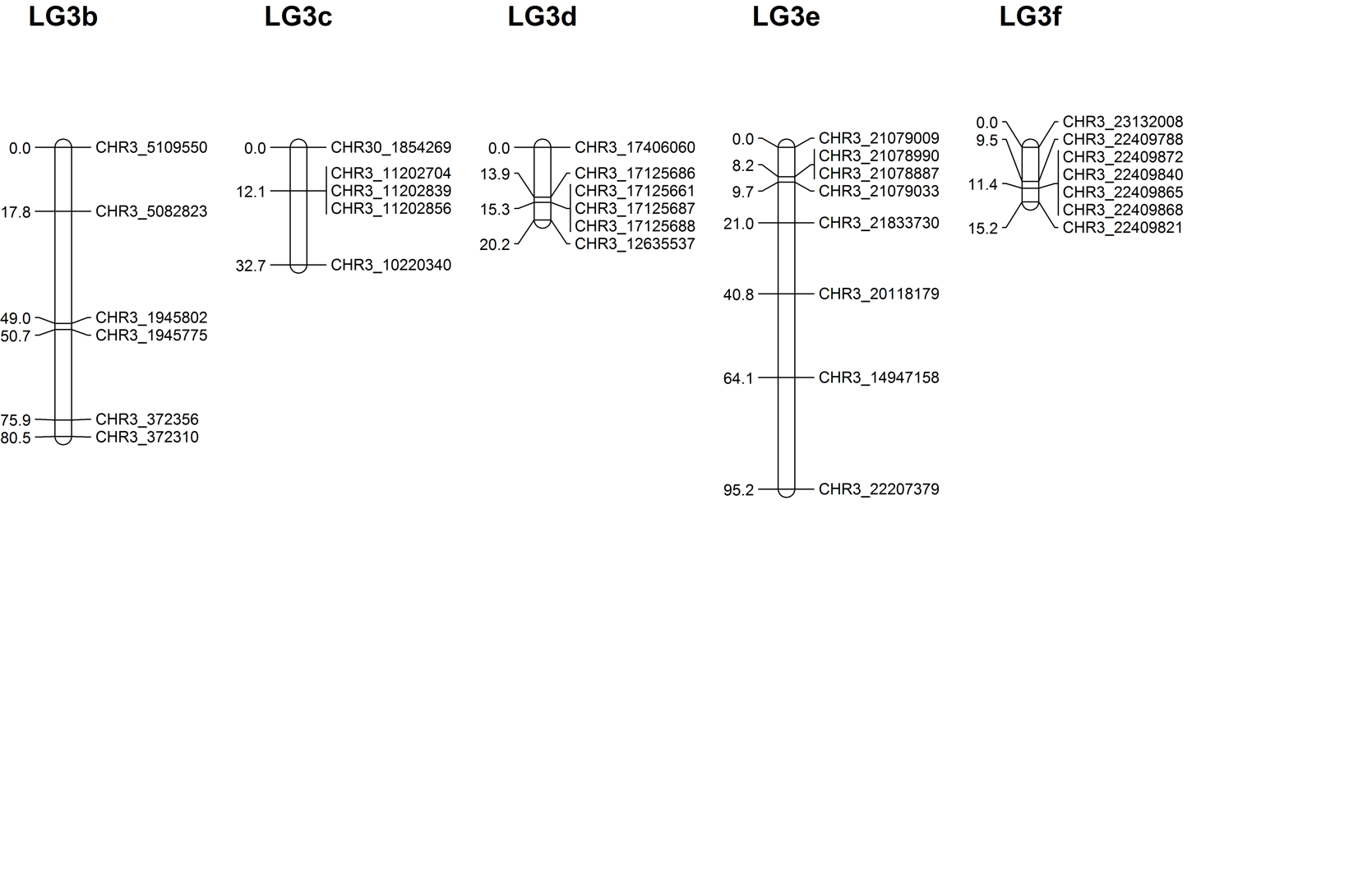
**

**Supplementary data 6.** Linkage maps (Paternal) continued

**
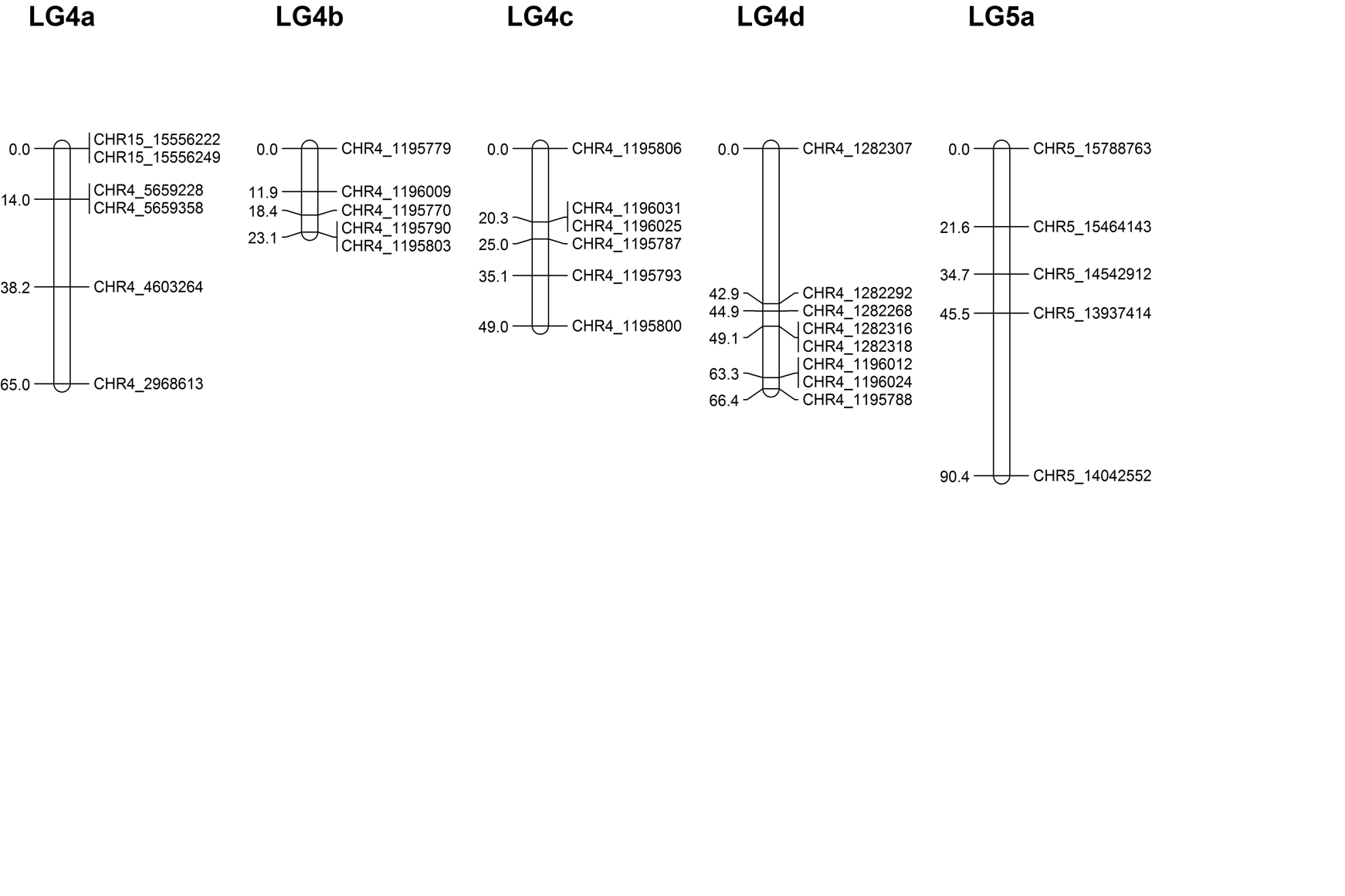

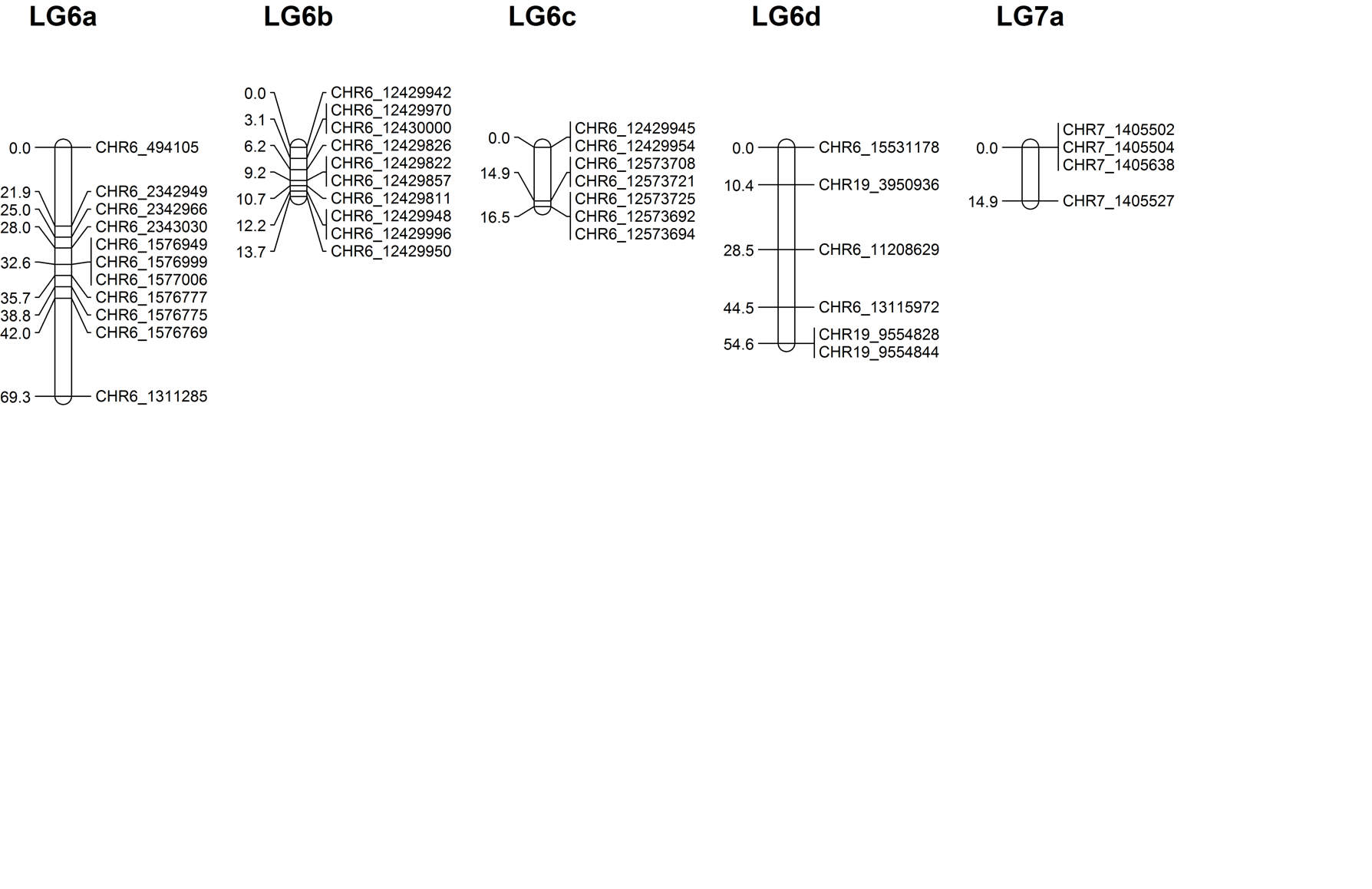
**

**Supplementary data 6.** Linkage maps (Paternal) continued

**
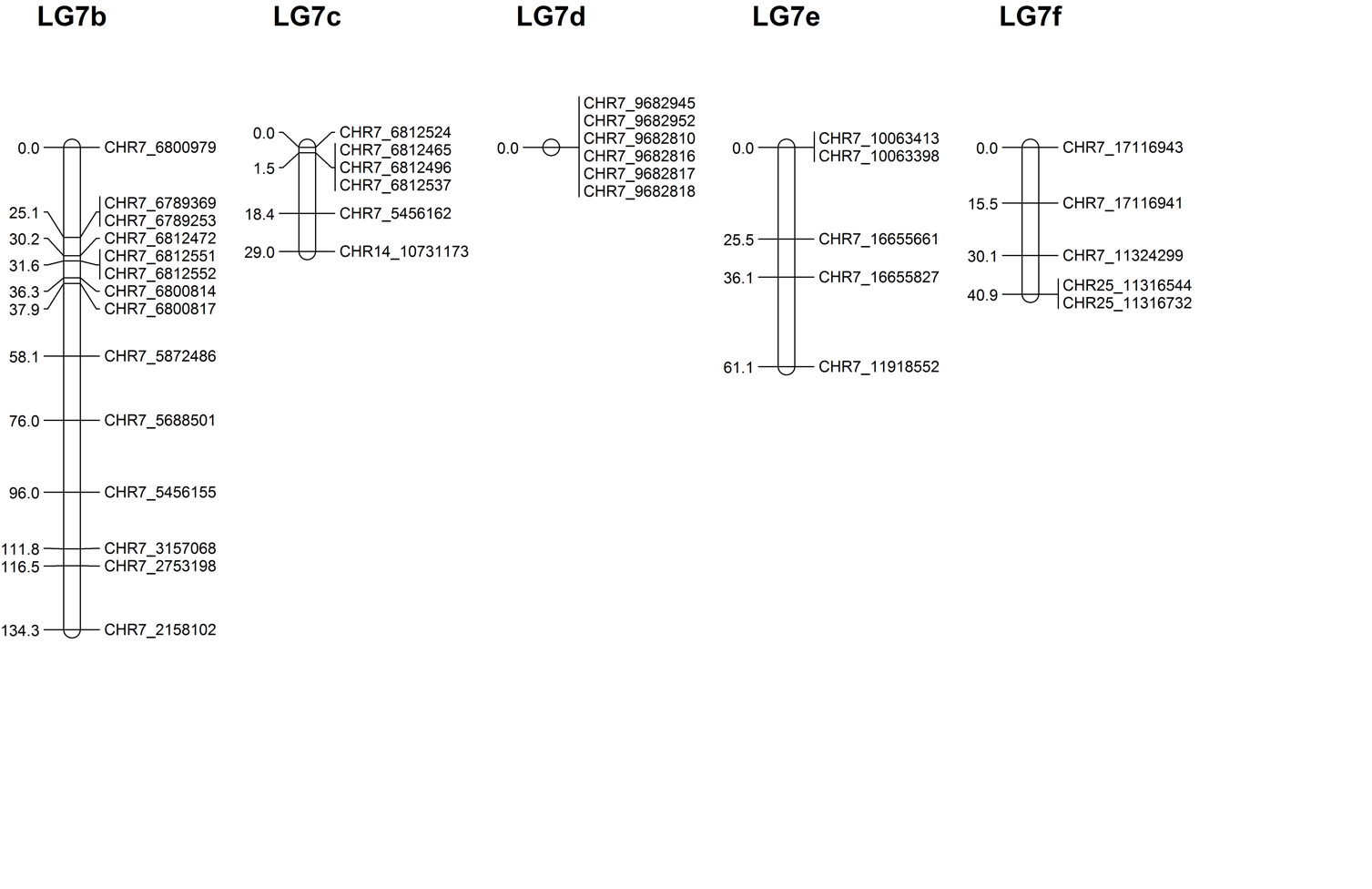
**

**Supplementary data 6.** Linkage maps (Paternal) continued

**
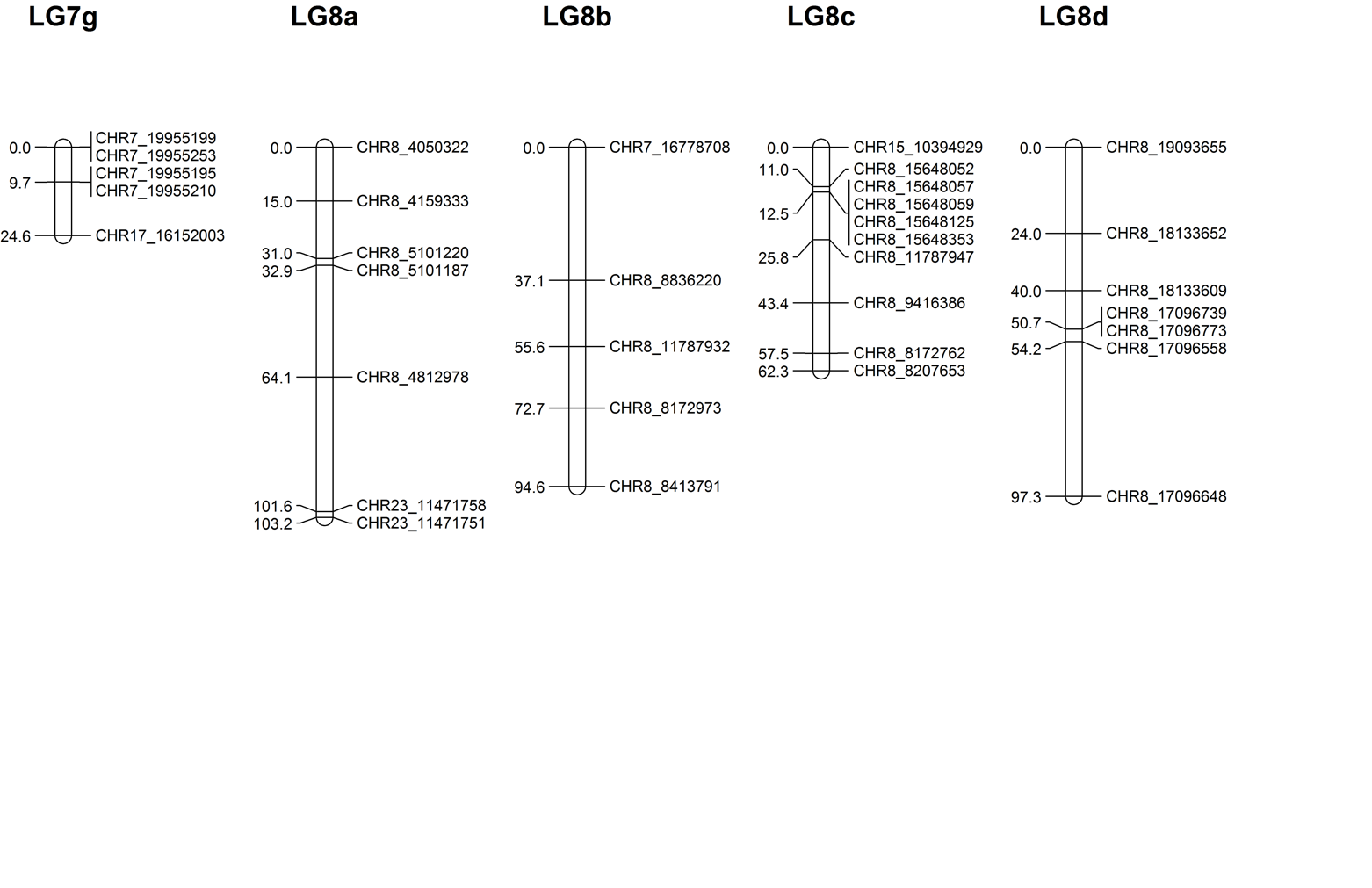
**

**
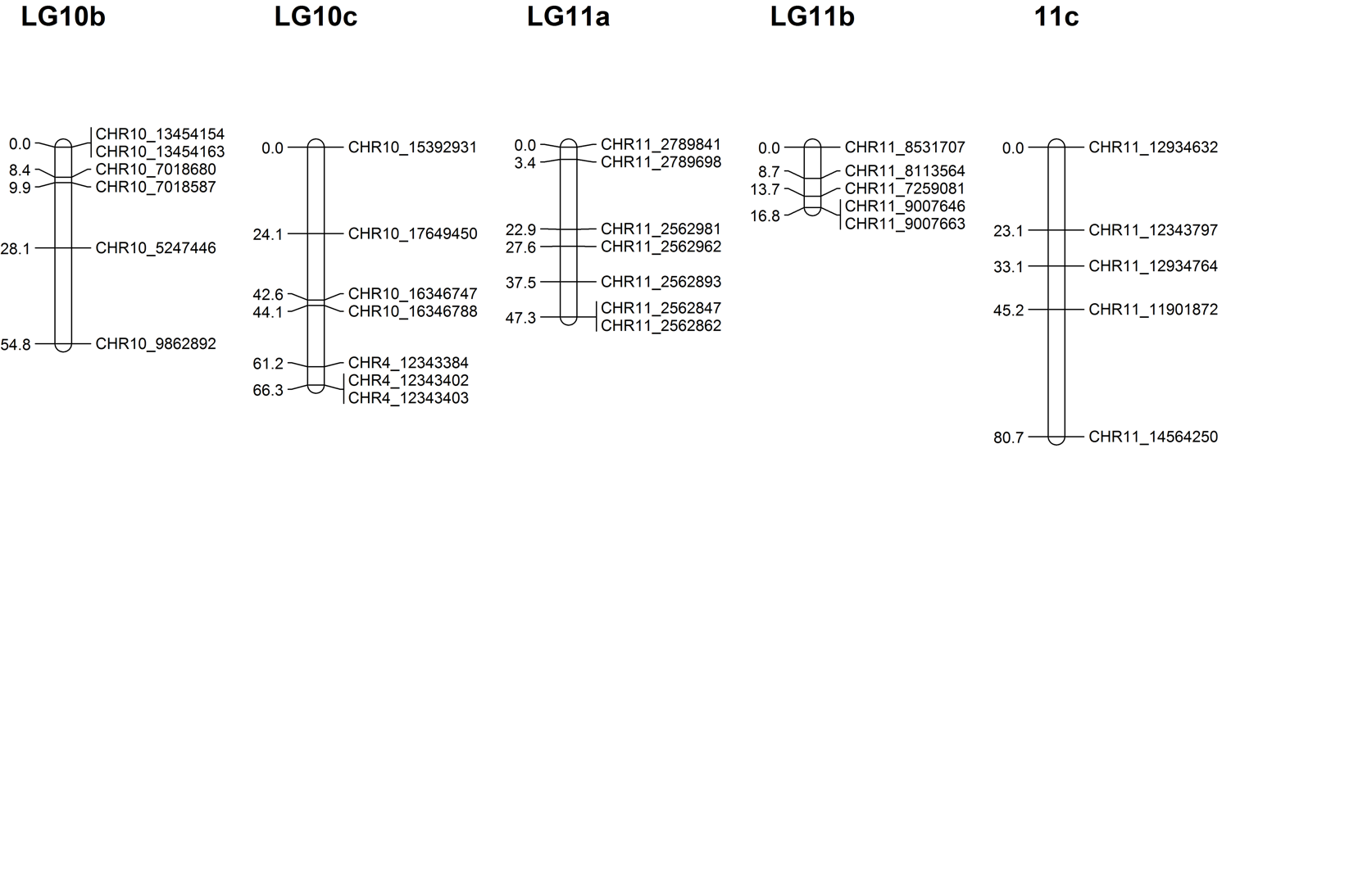
**

**Supplementary data 6.** Linkage maps (Paternal) continued

**
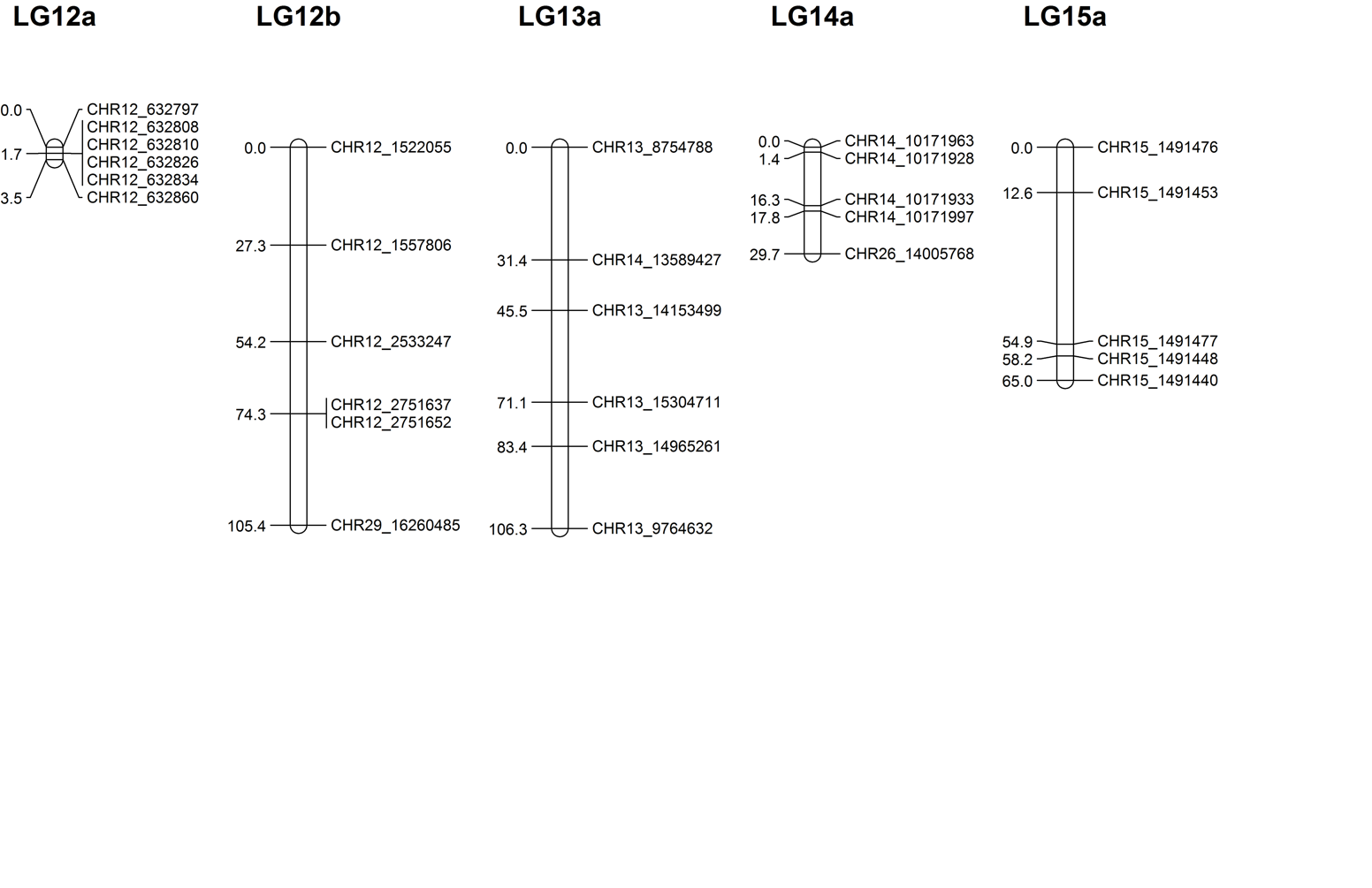

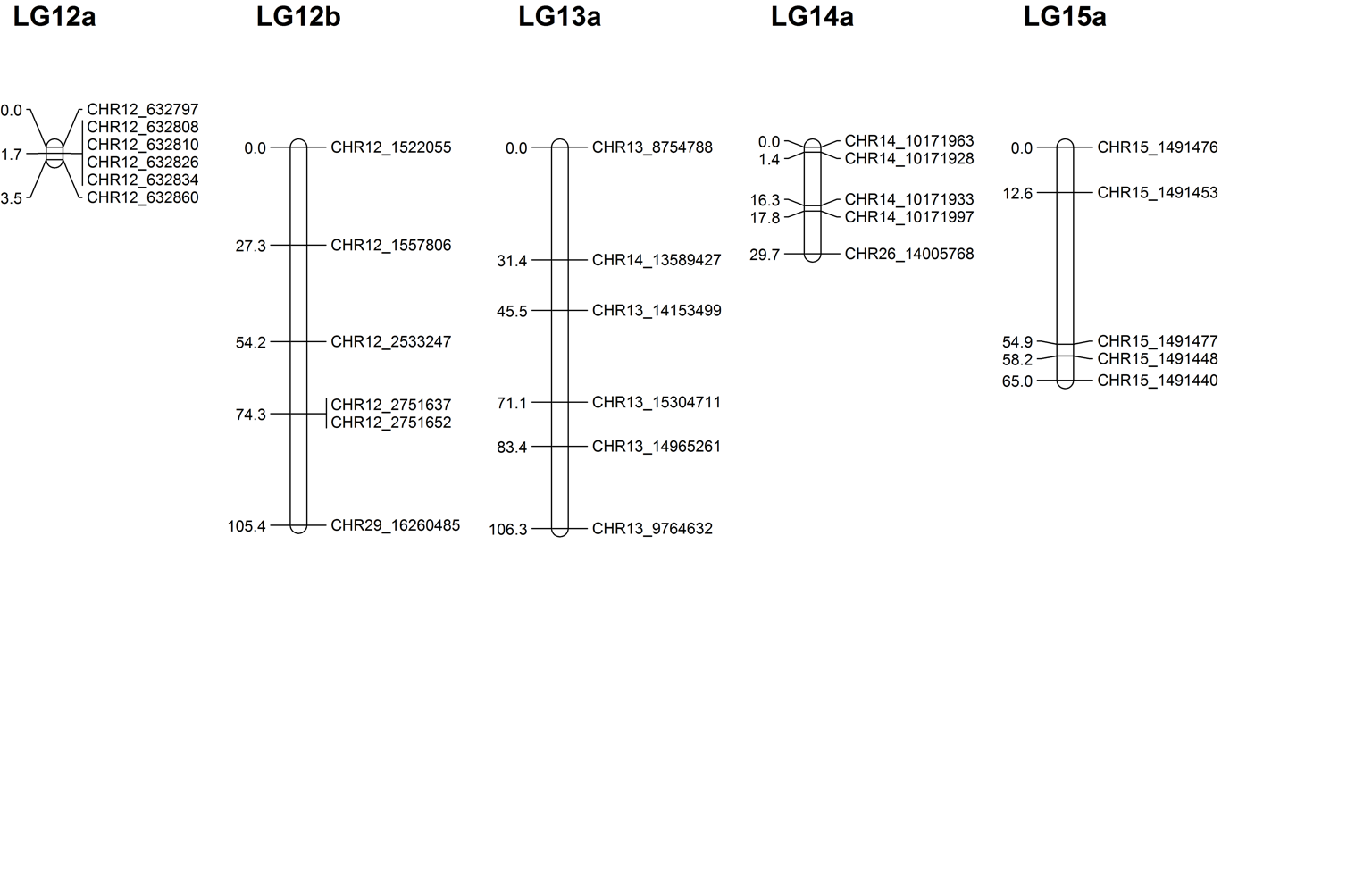
**

**Supplementary data 6.** Linkage maps (Paternal) continued

**
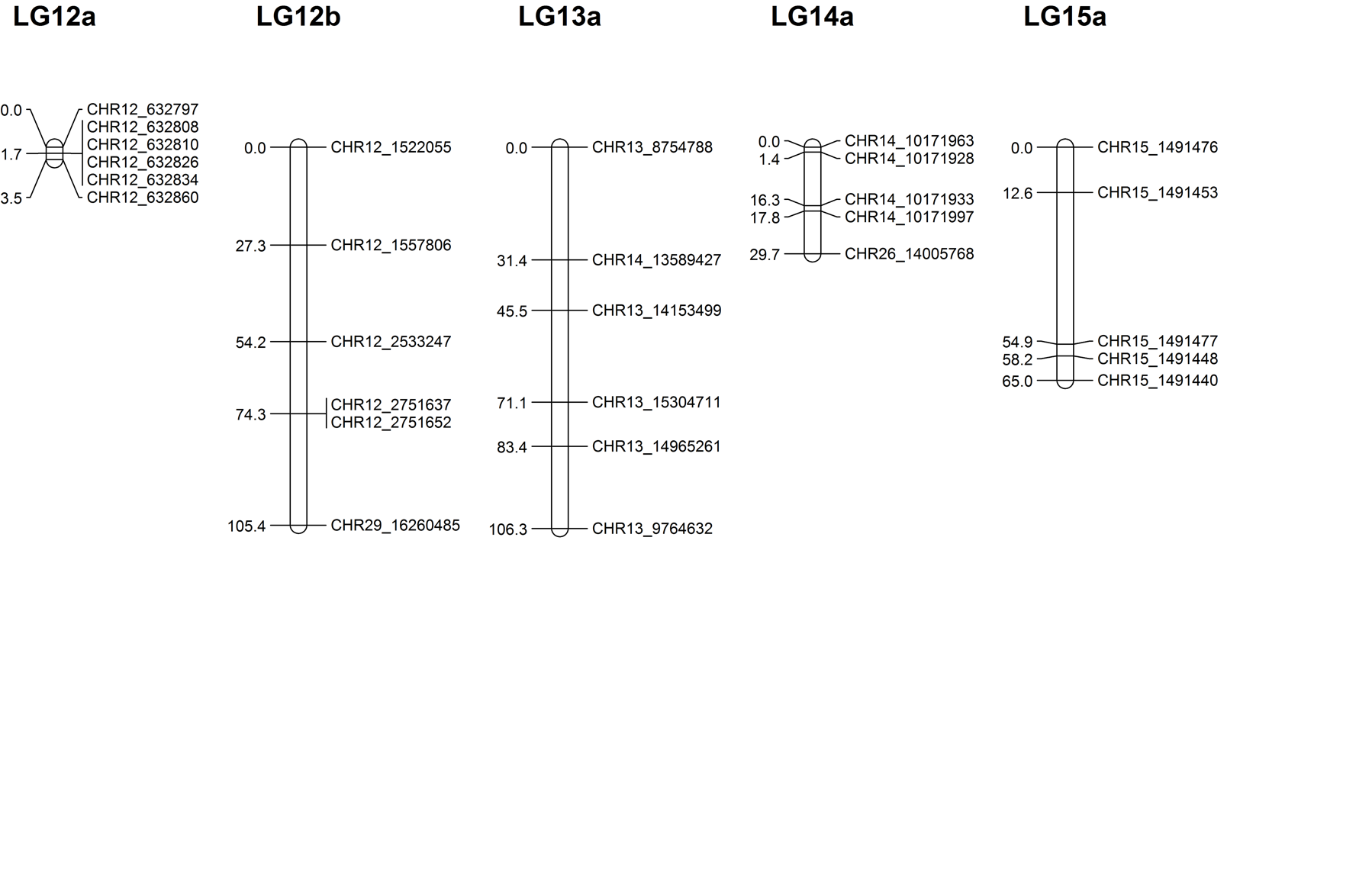
**

**Supplementary data 6.** Linkage maps (Paternal) continued

**
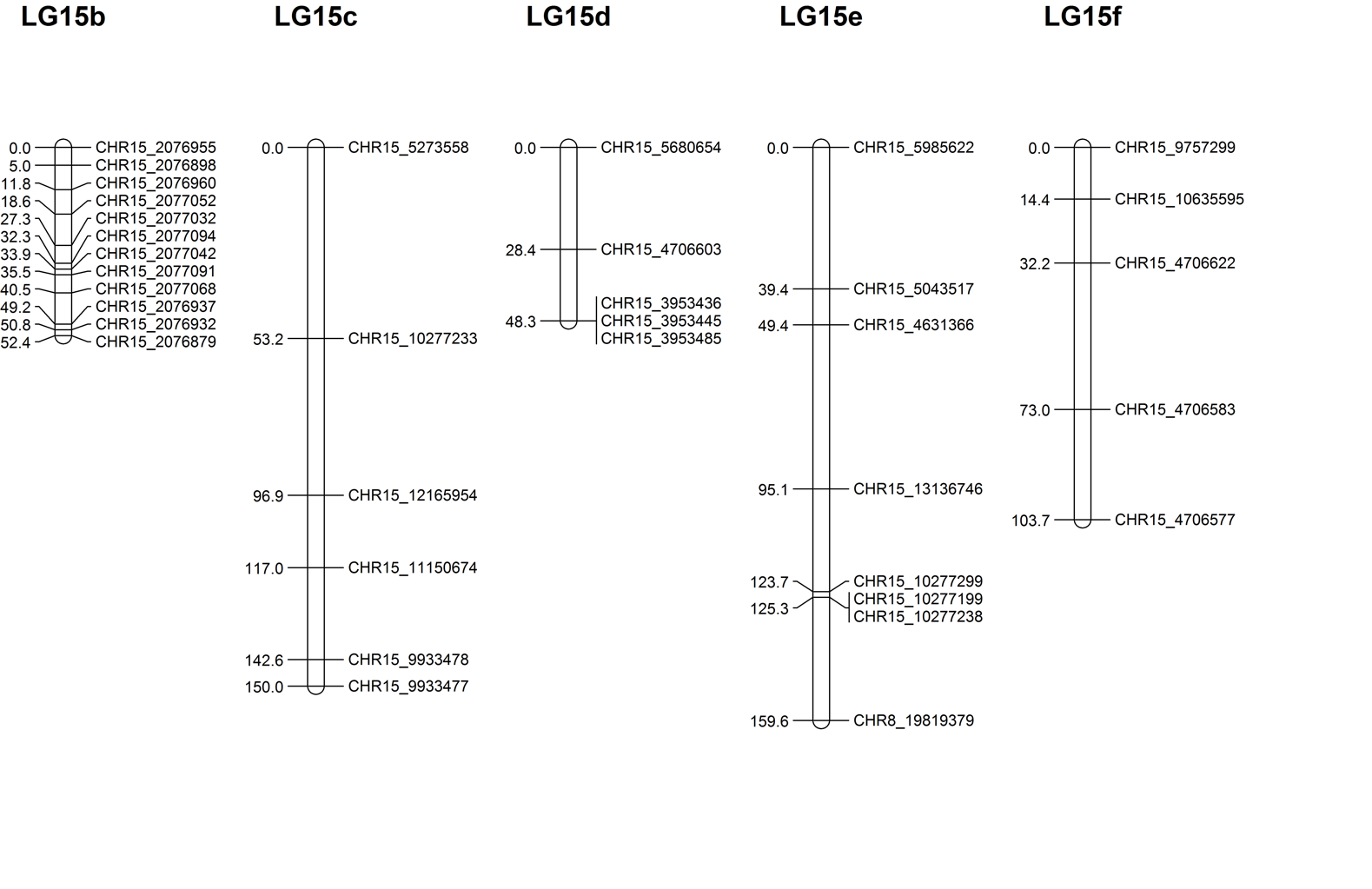
**

**Supplementary data 6.** Linkage maps (Paternal) continued

**
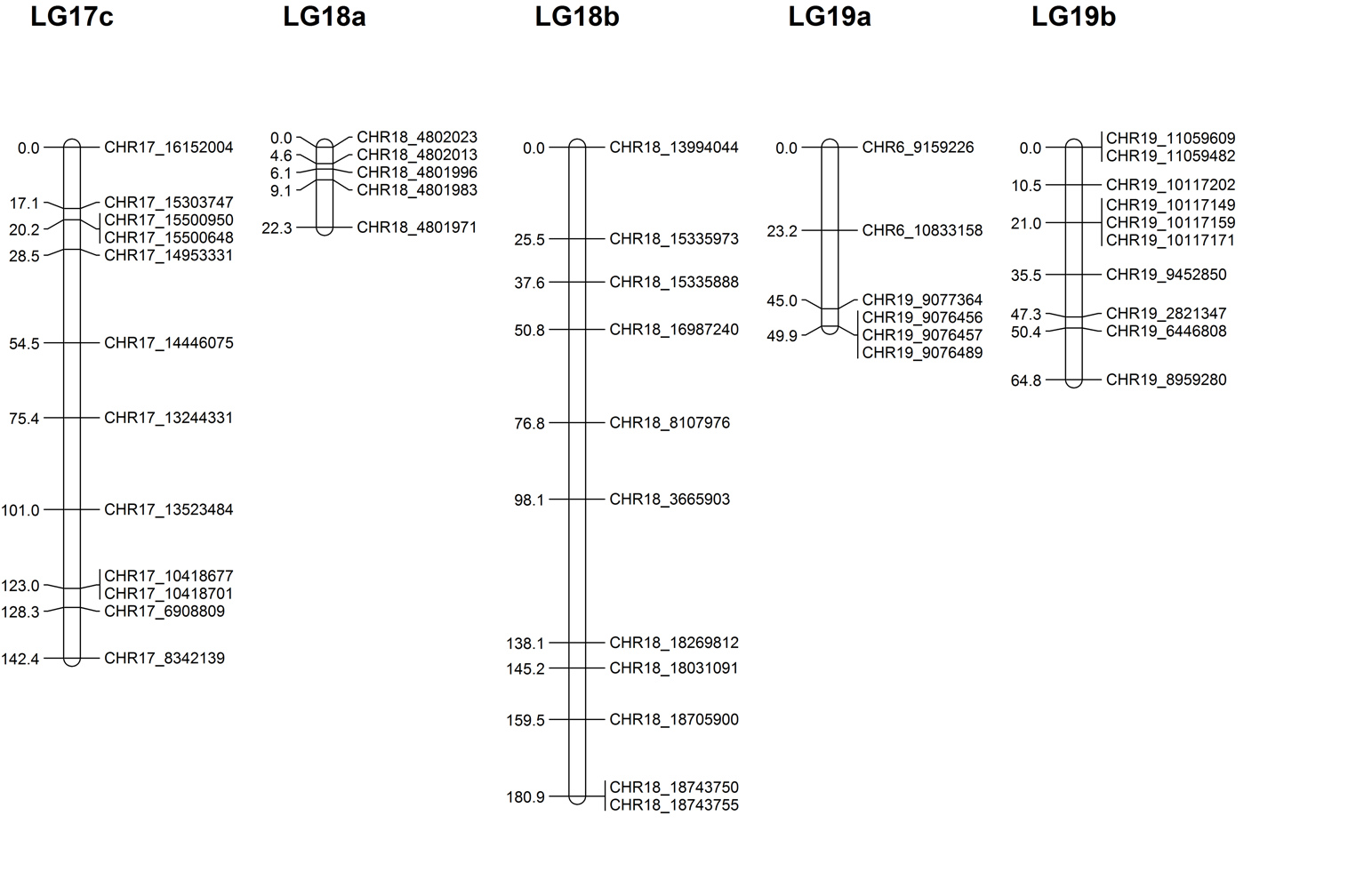
**

**Supplementary data 6.** Linkage maps (Paternal) continued

**
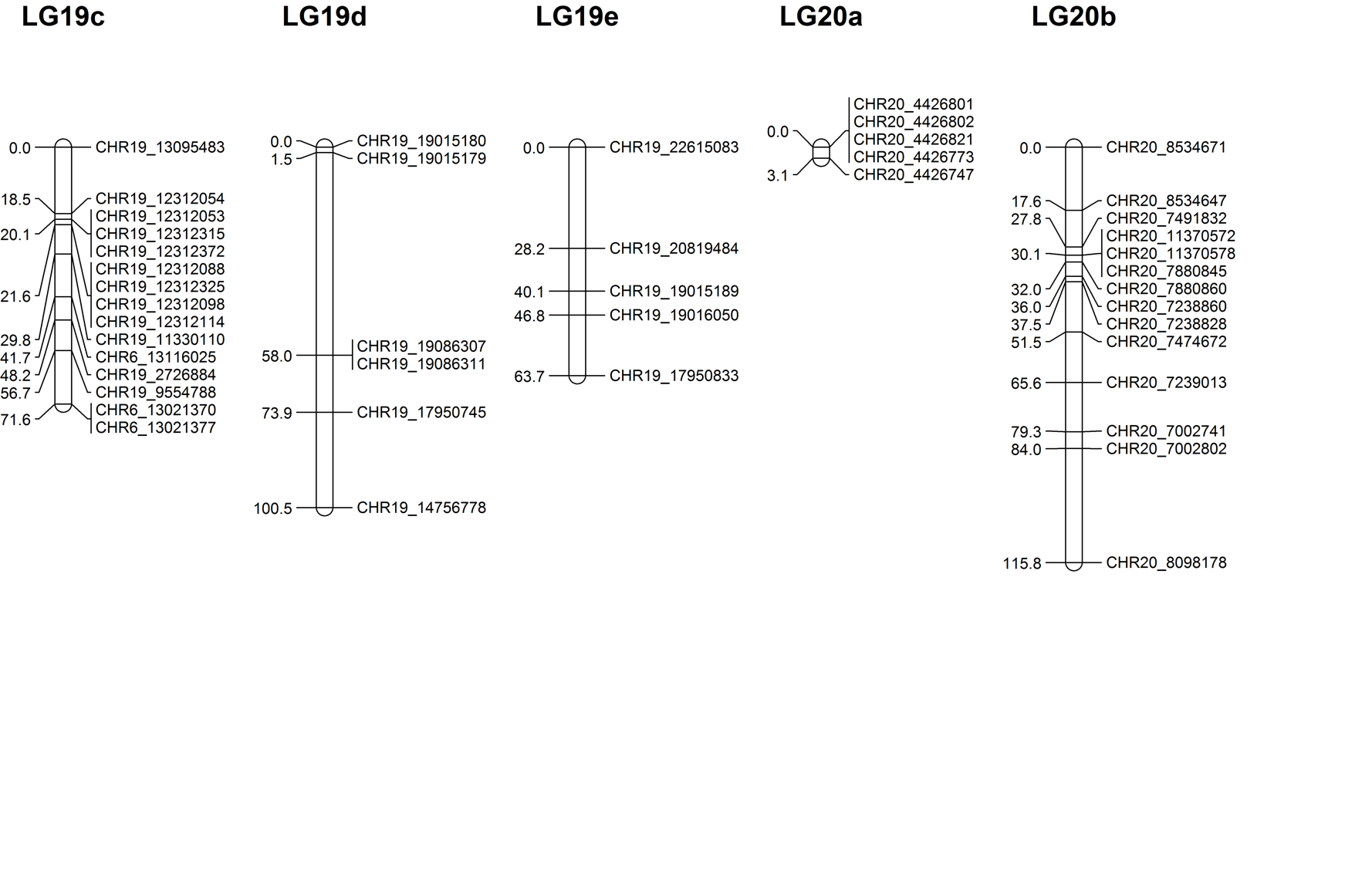
**

**Supplementary data 6.** Linkage maps (Paternal) continued

**
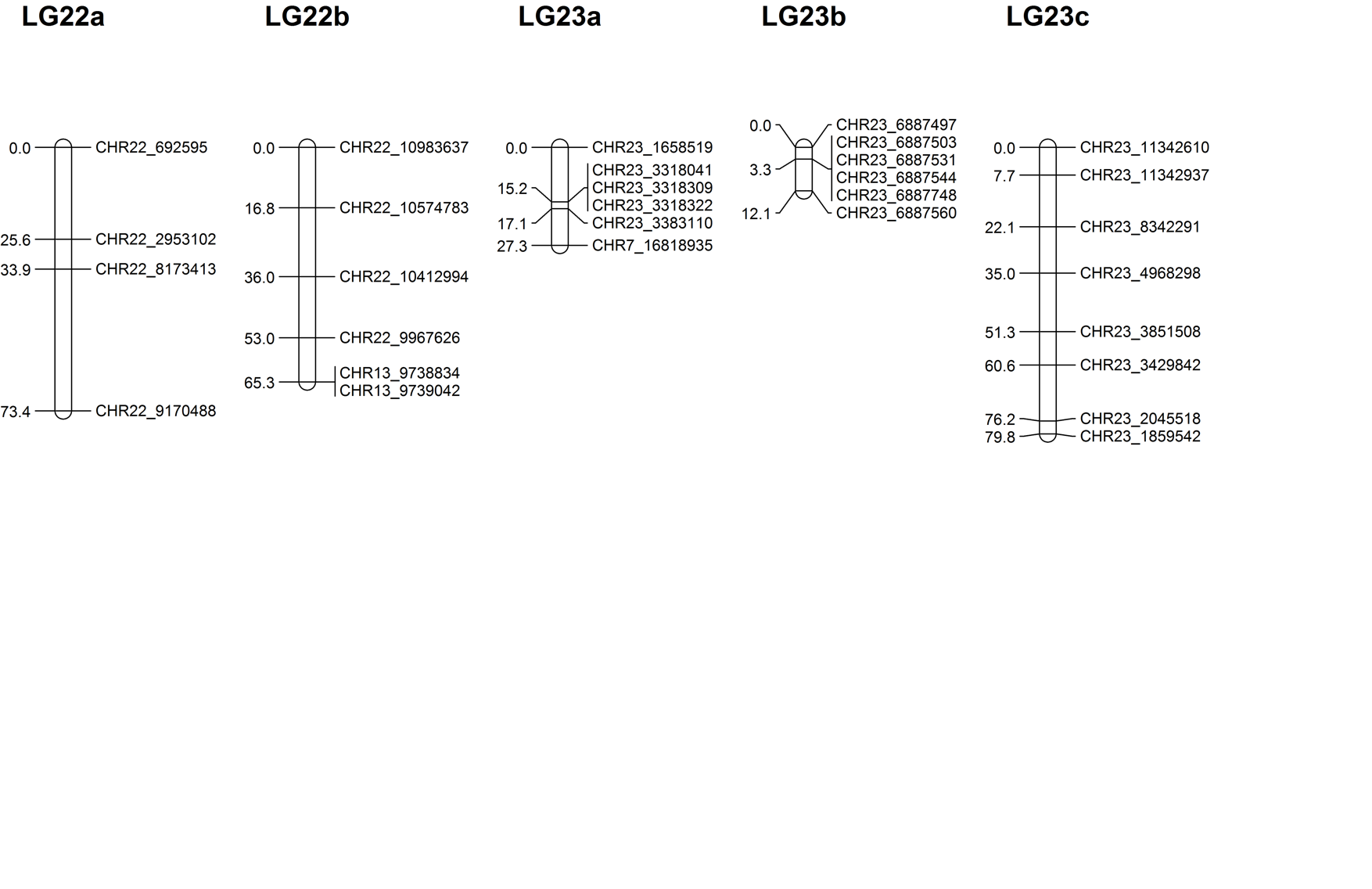

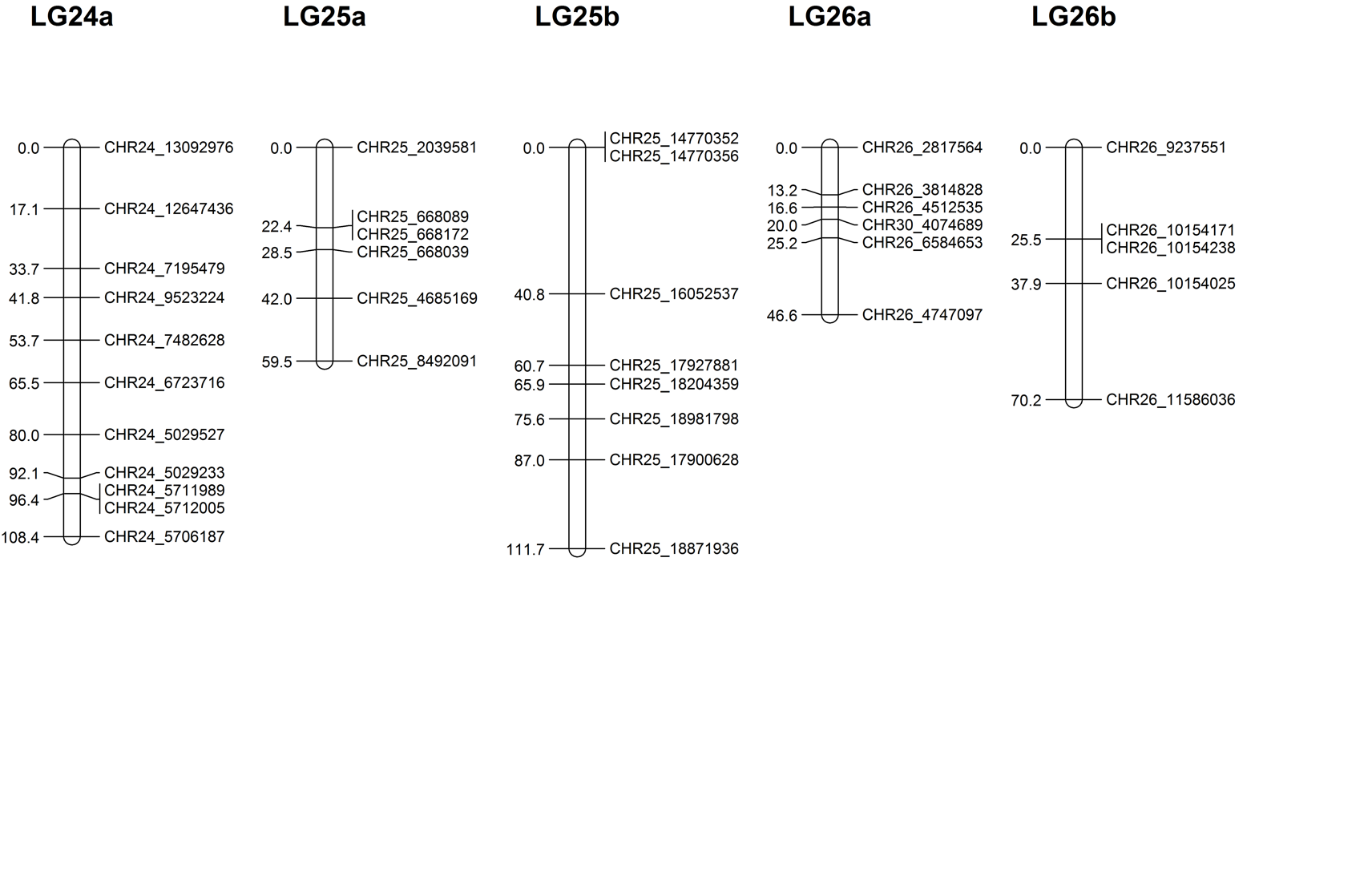
**

**Supplementary data 6.** Linkage maps (Paternal) continued**
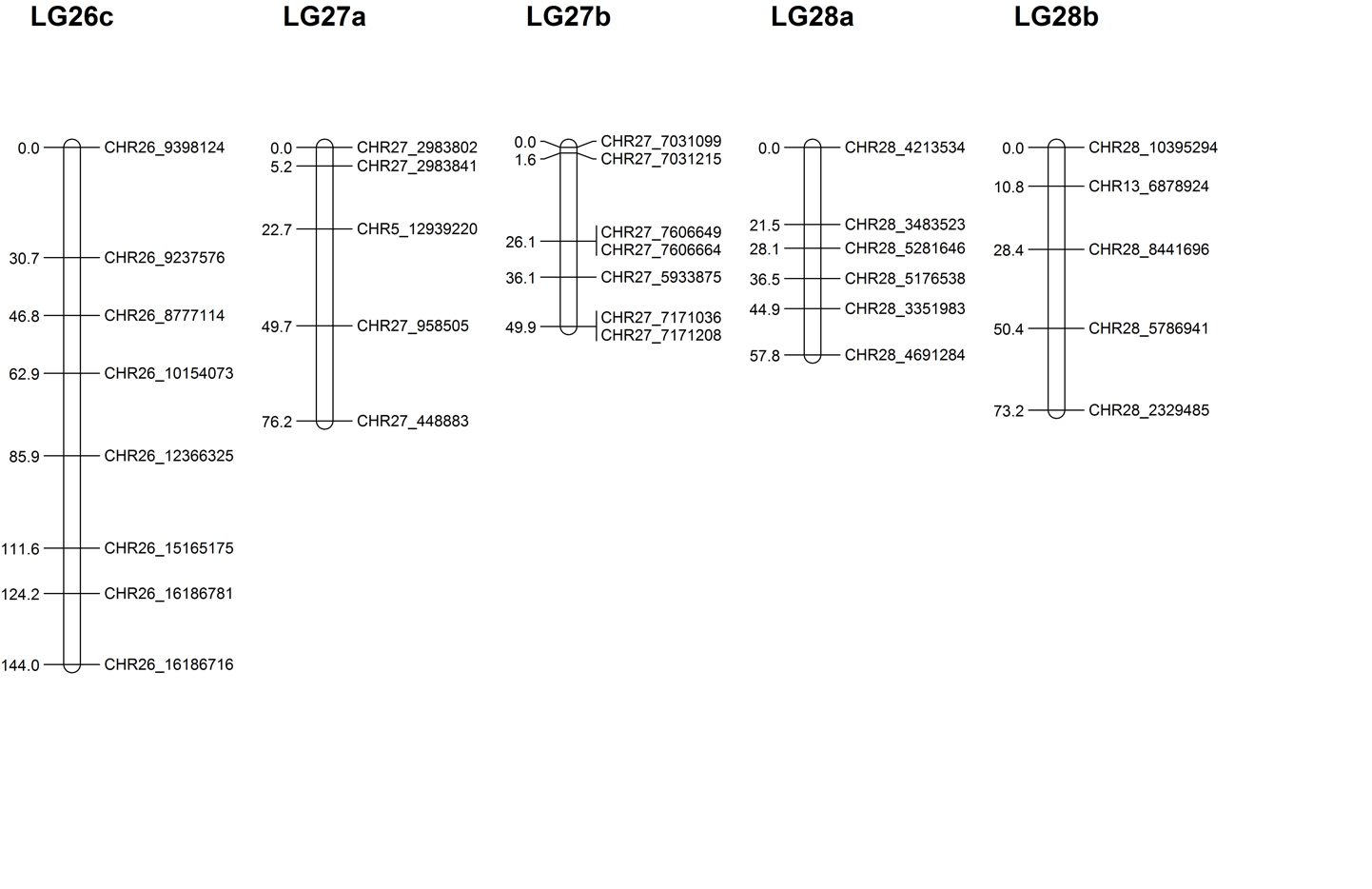

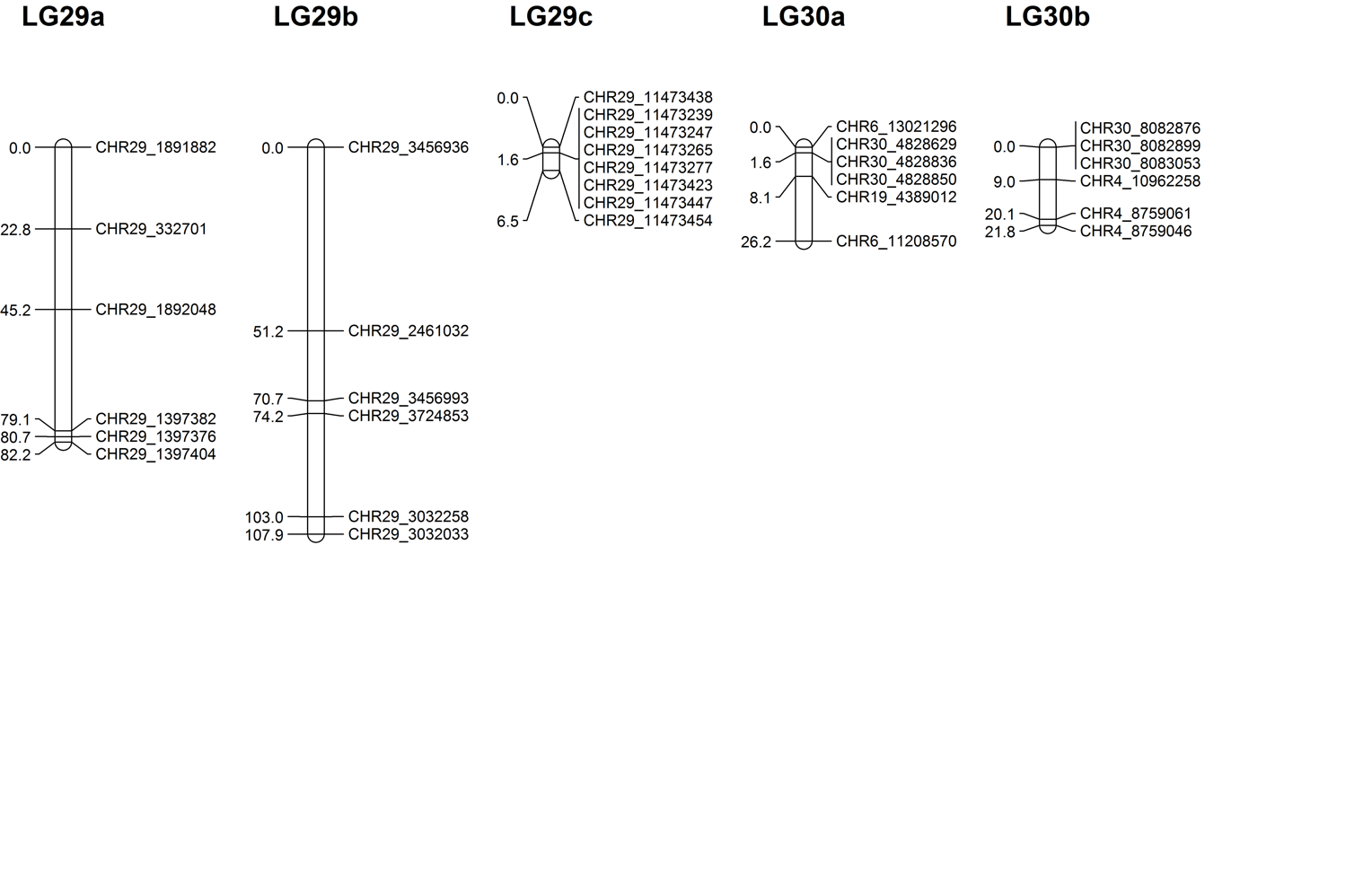
**

**Supplementary data 7.** Linkage maps (Maternal)

**
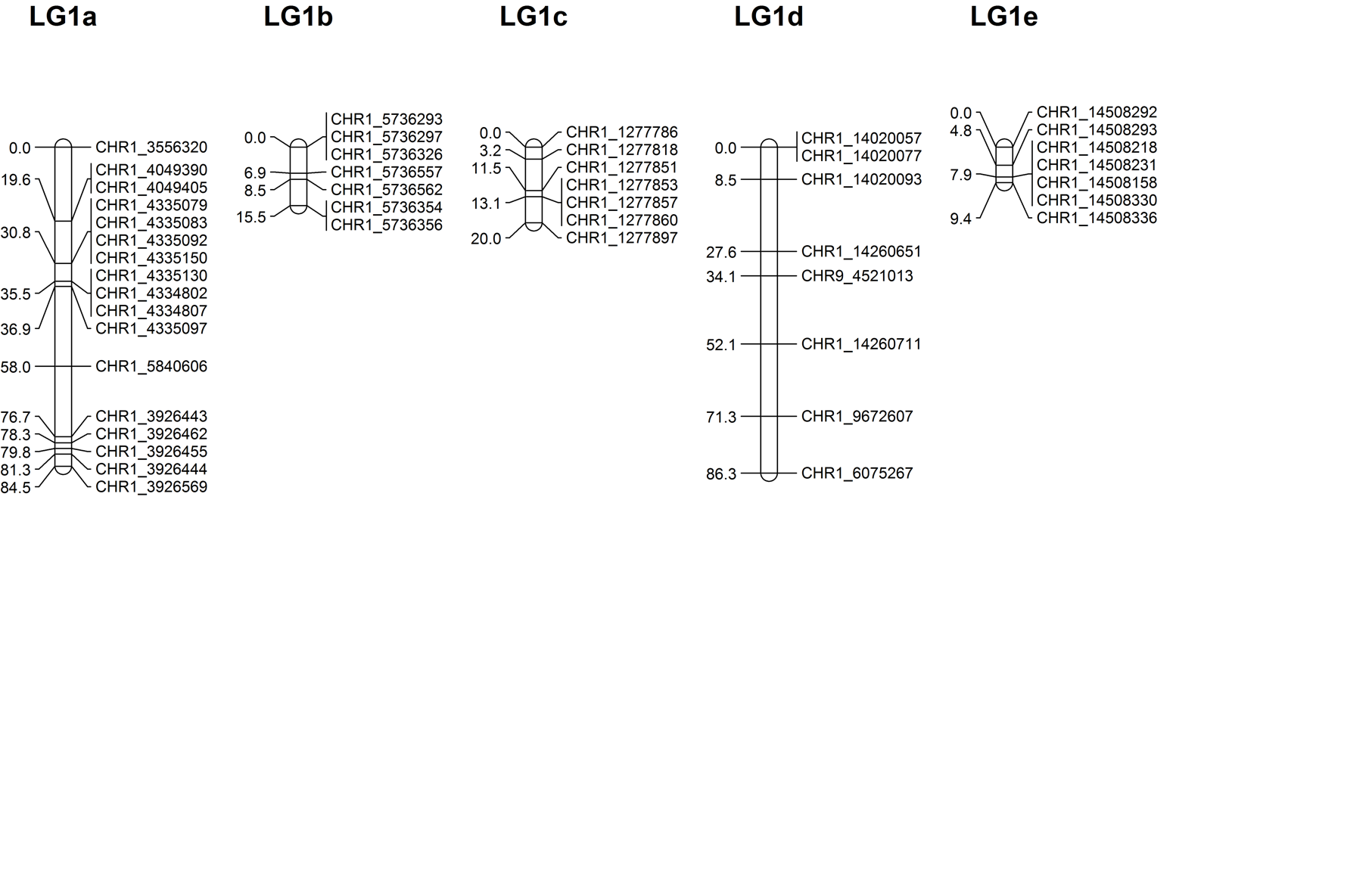
**

**
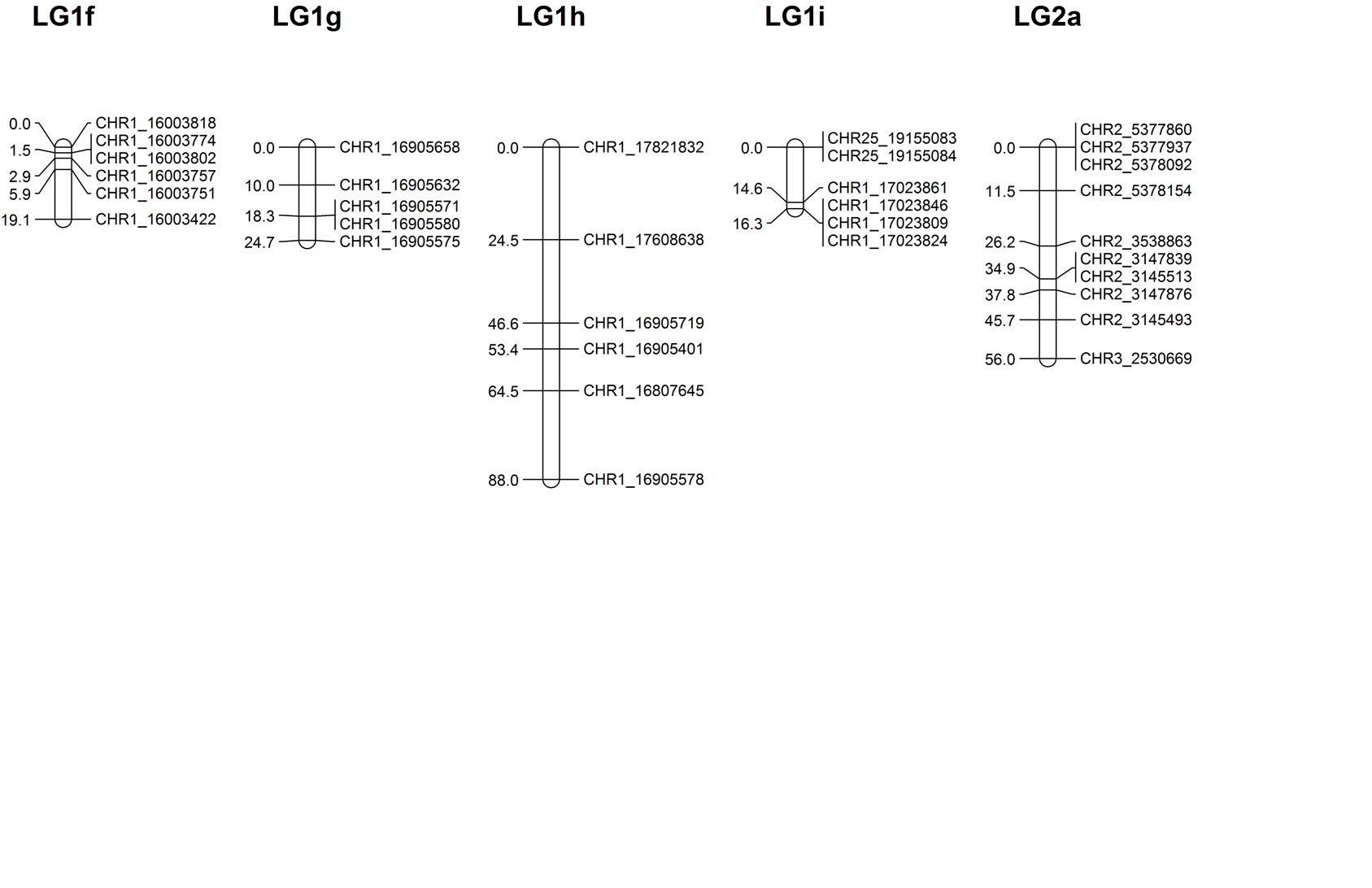
**

**Supplementary data 7.** Linkage maps (Maternal) continued

**
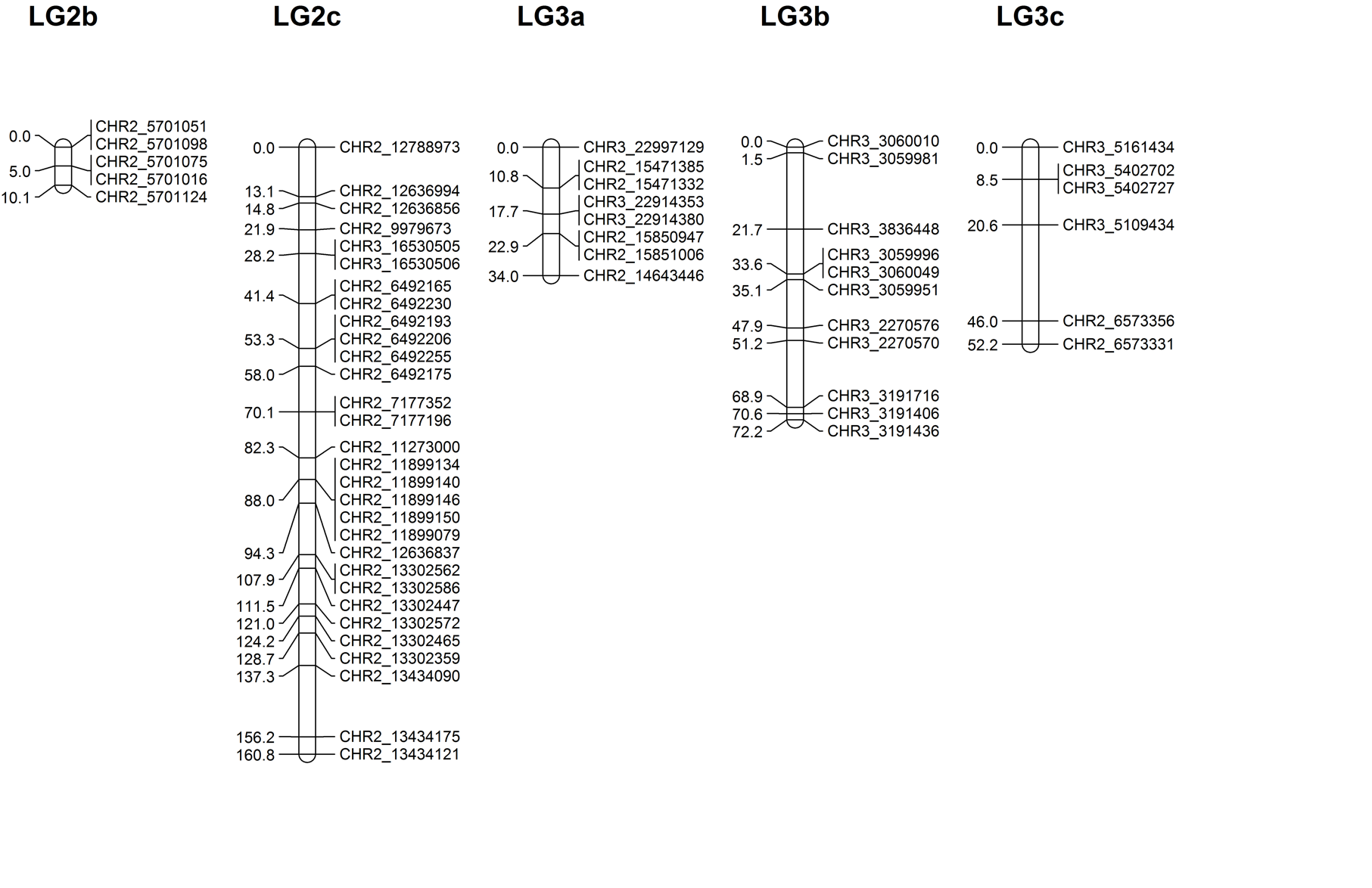
**

**Supplementary data 7.** Linkage maps (Maternal) continued**
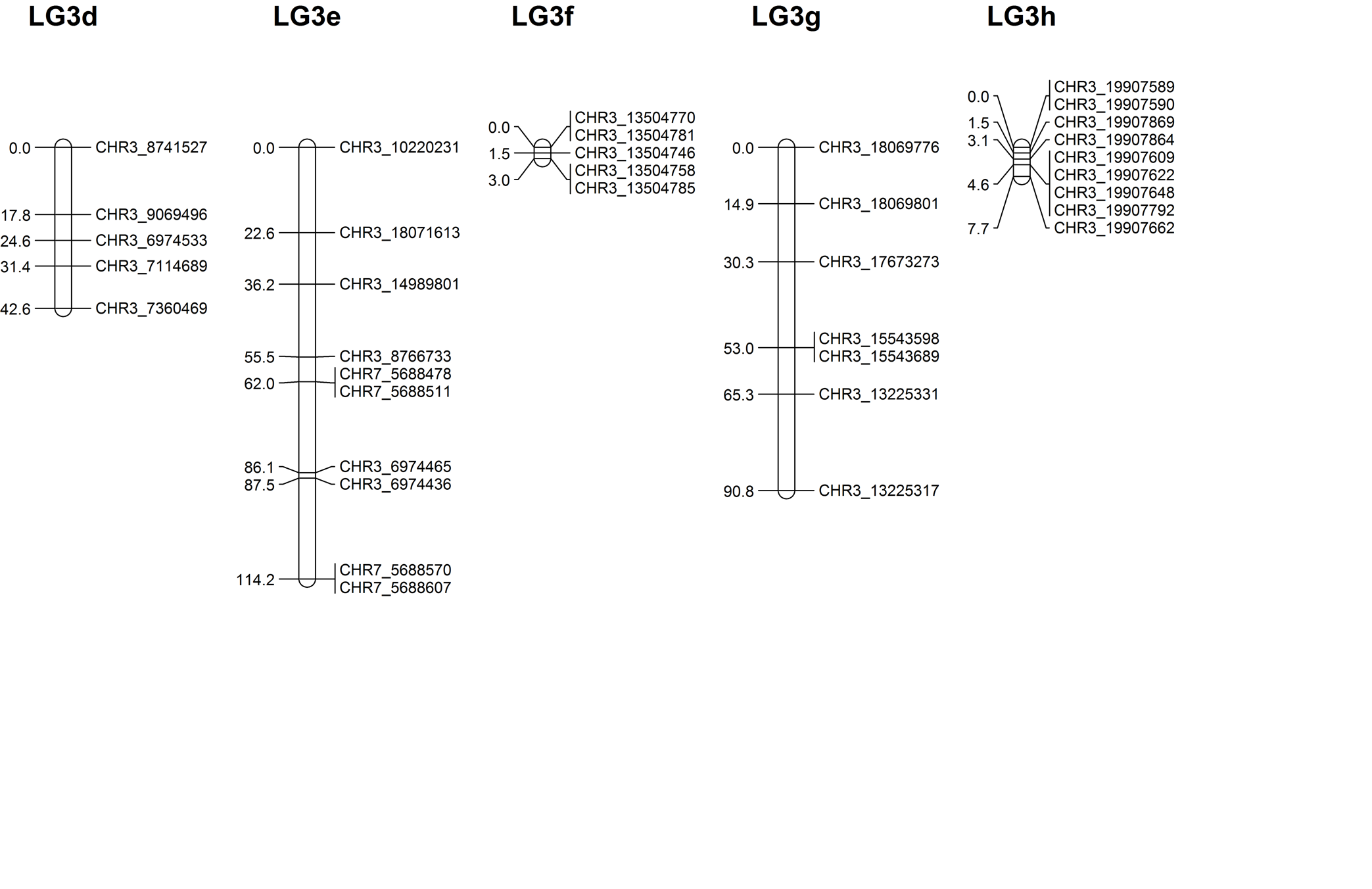
**

**Supplementary data 7.** Linkage maps (Maternal) continued

**
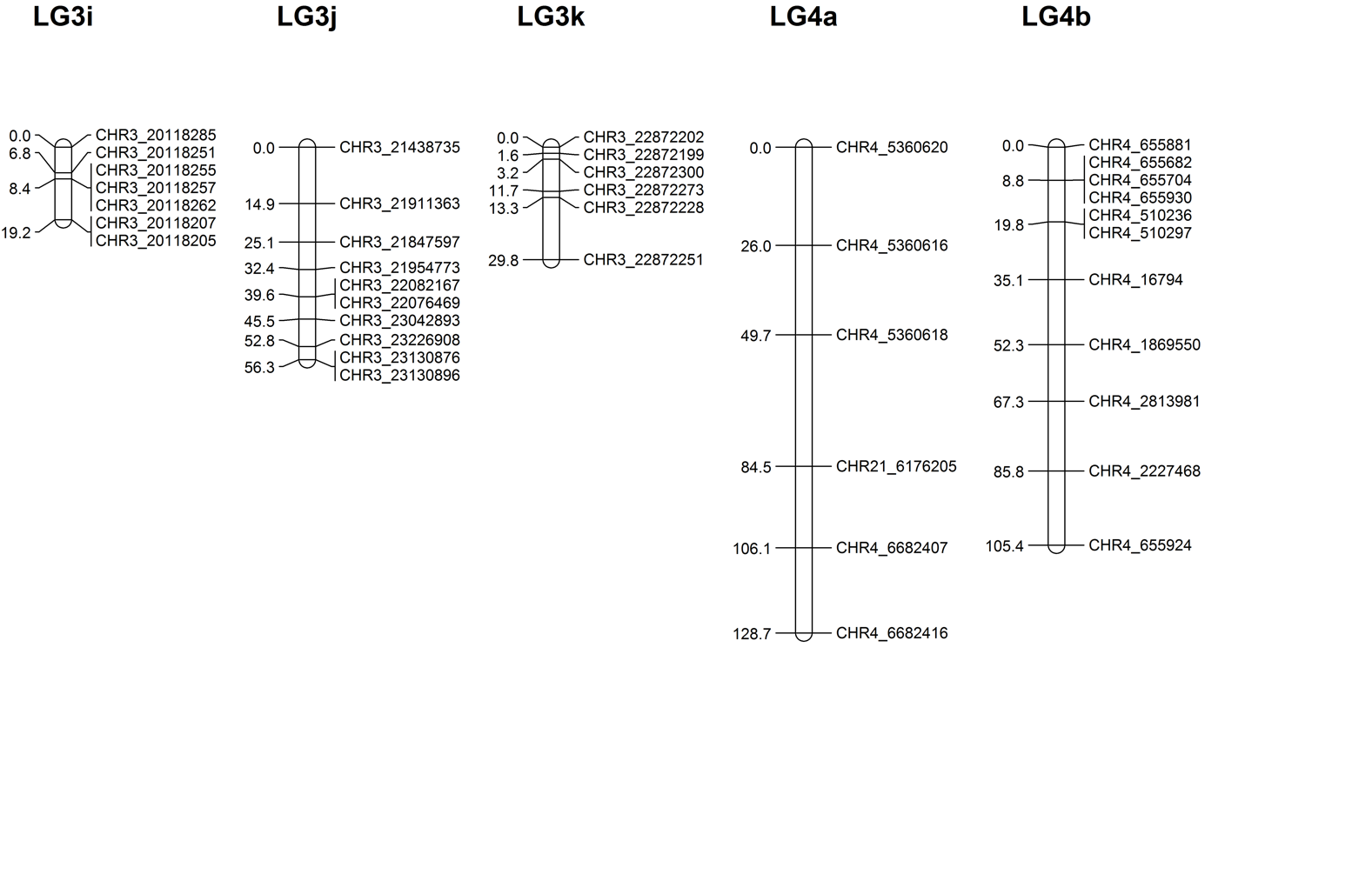
**

**Supplementary data 7.** Linkage maps (Maternal) continued

**
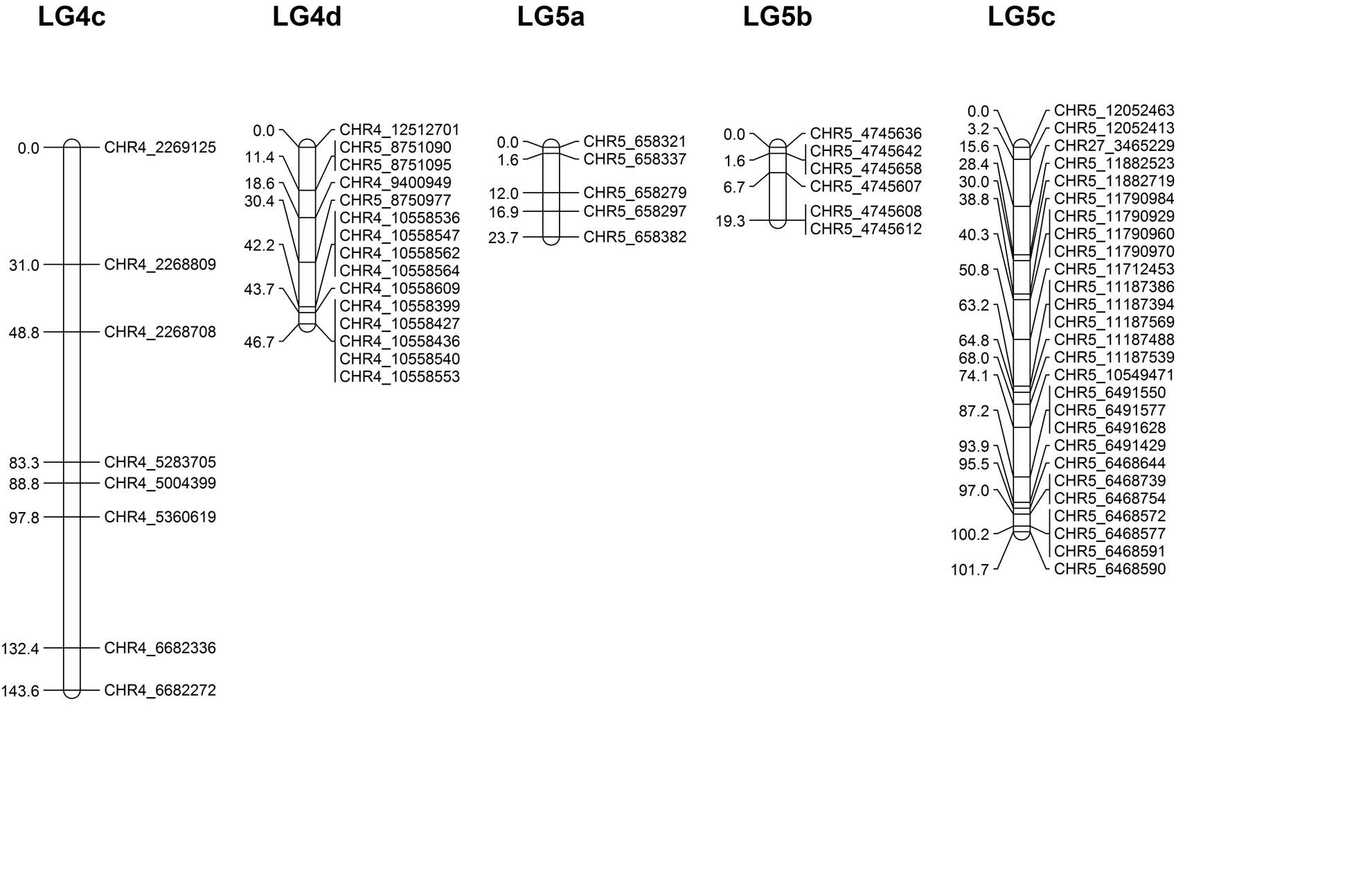
**

**Supplementary data 7.** Linkage maps (Maternal) continued

**
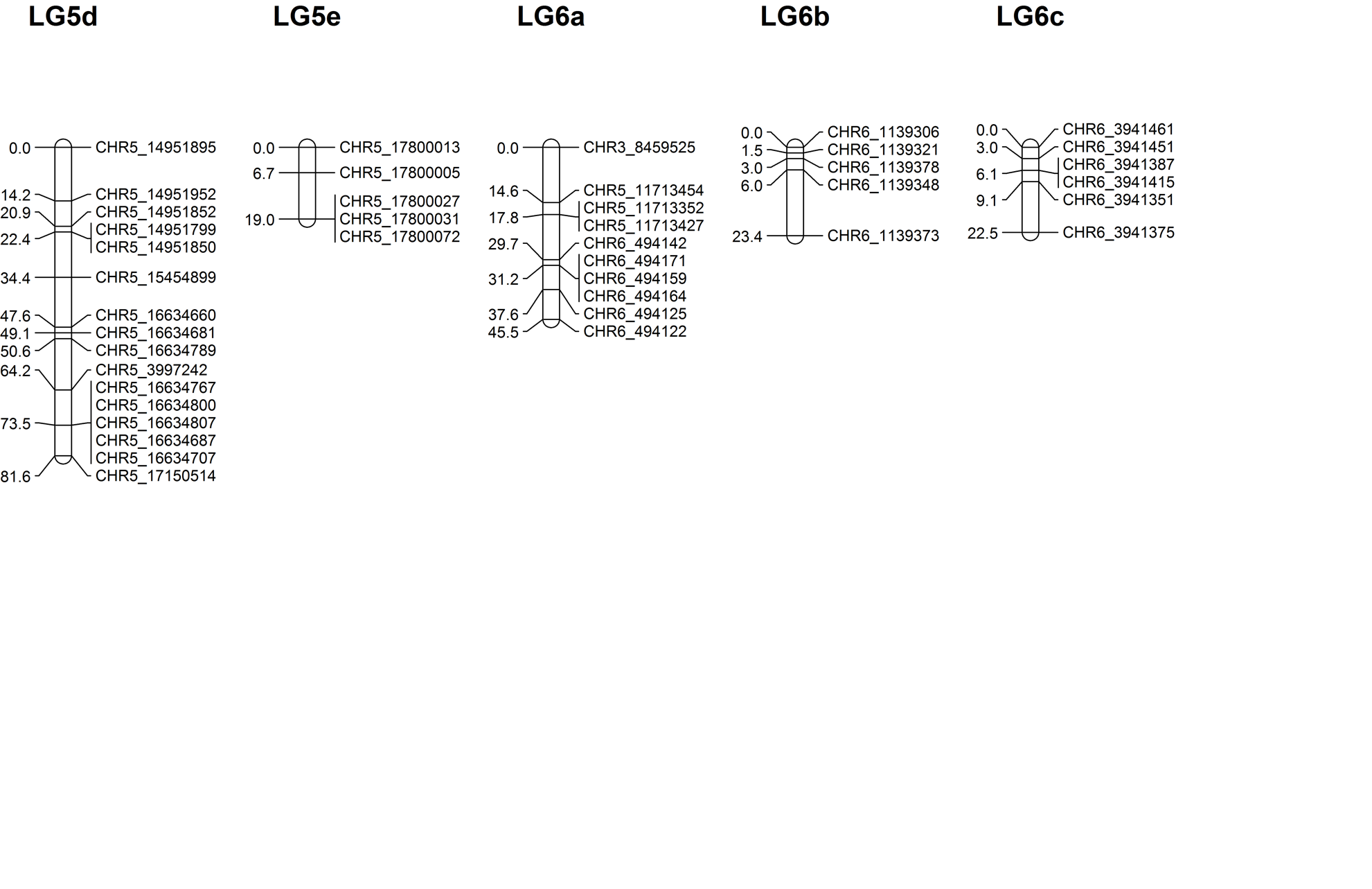
**

**Supplementary data 7.** Linkage maps (Maternal) continued**
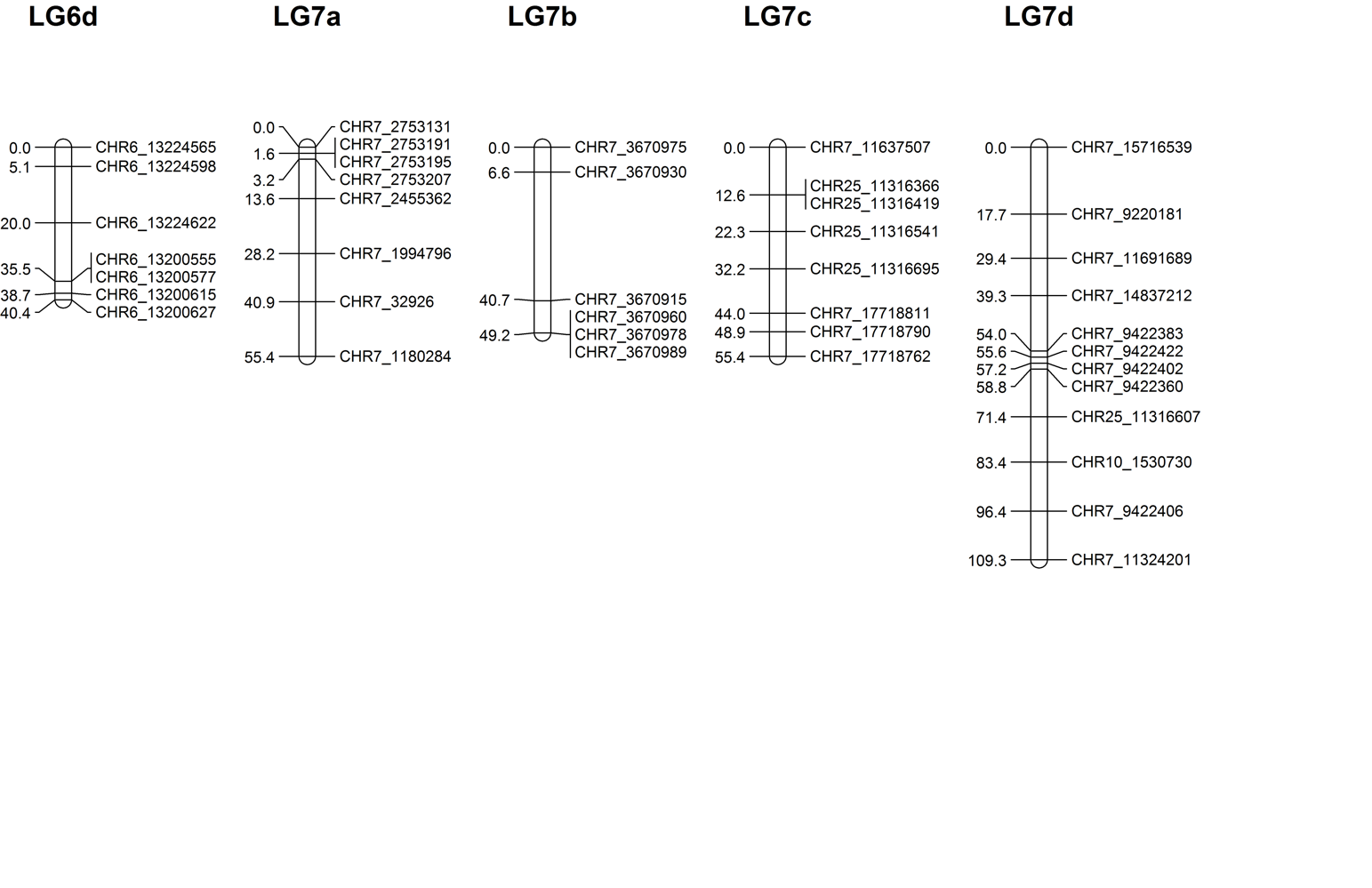
**

**
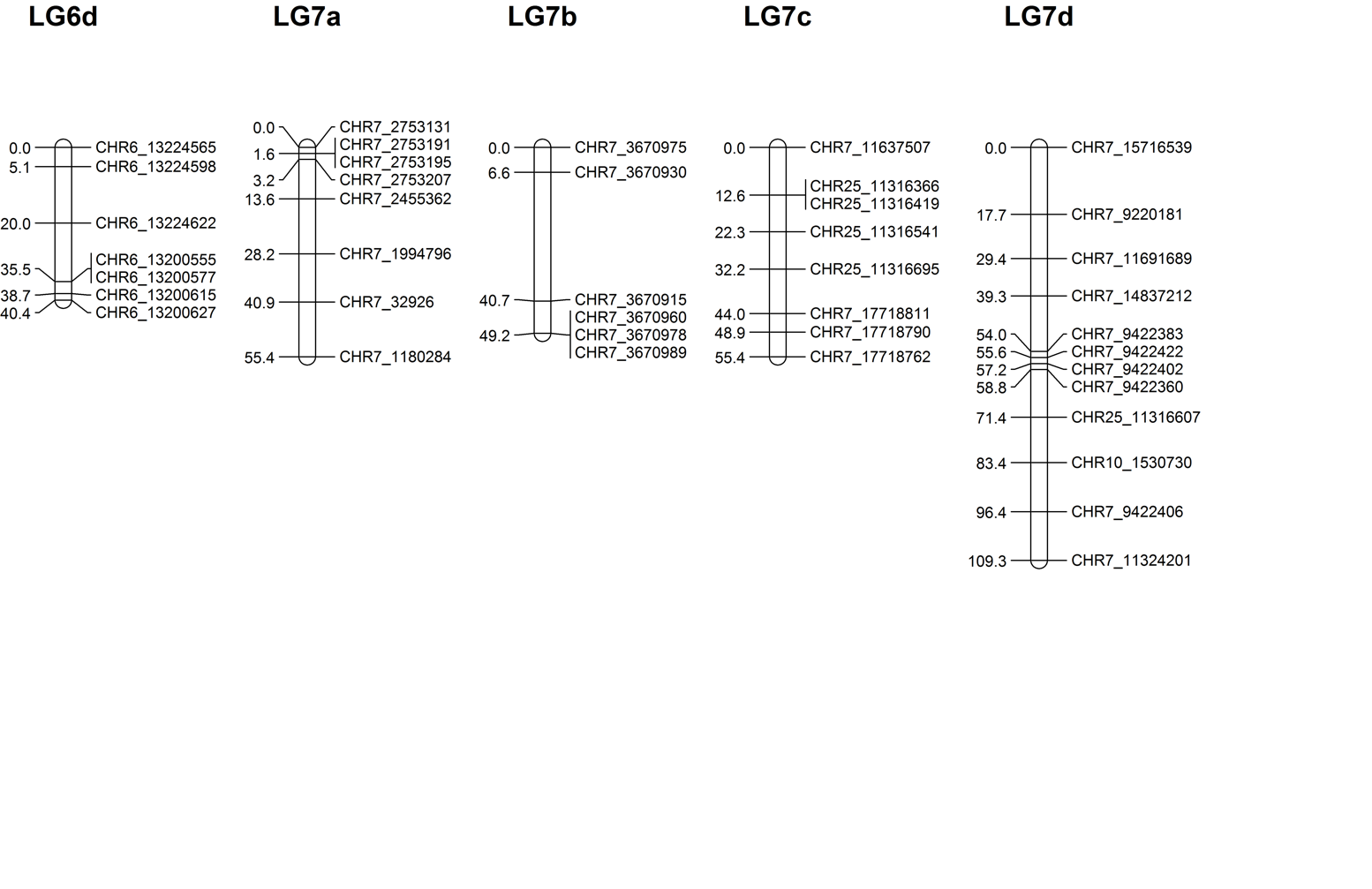
**

**Supplementary data 7.** Linkage maps (Maternal) continued**
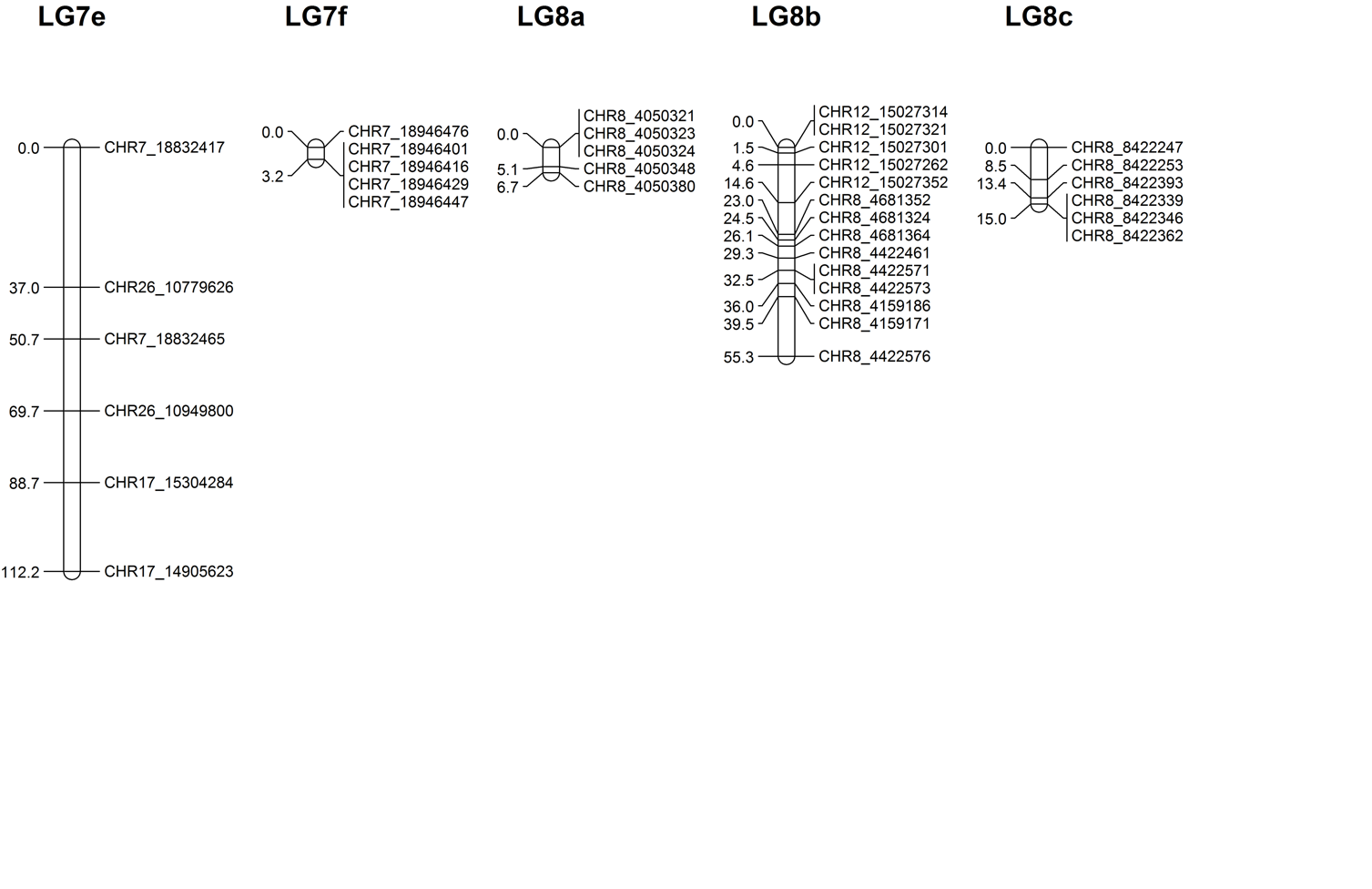

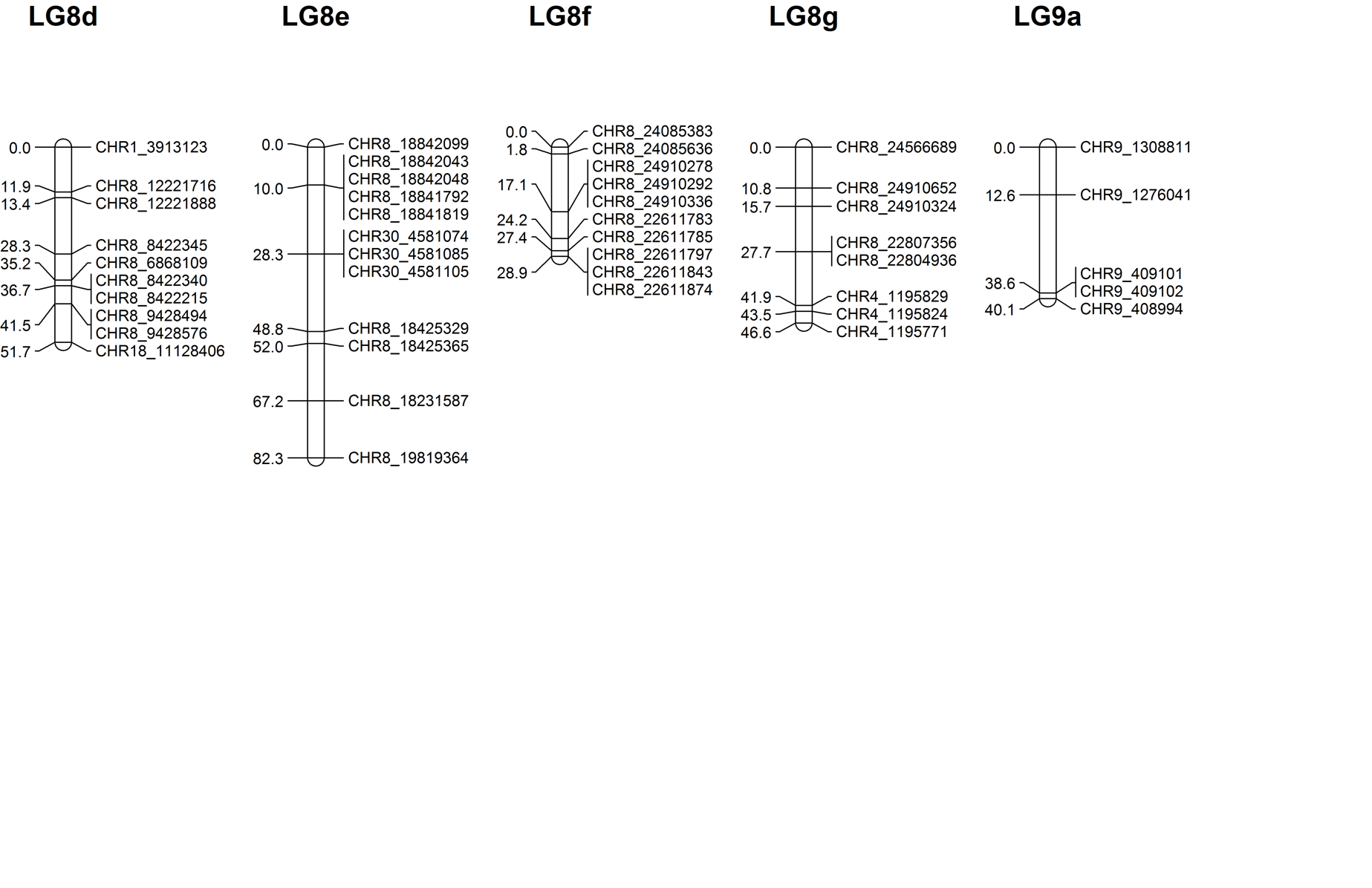
**

**Supplementary data 7.** Linkage maps (Maternal) continued

**
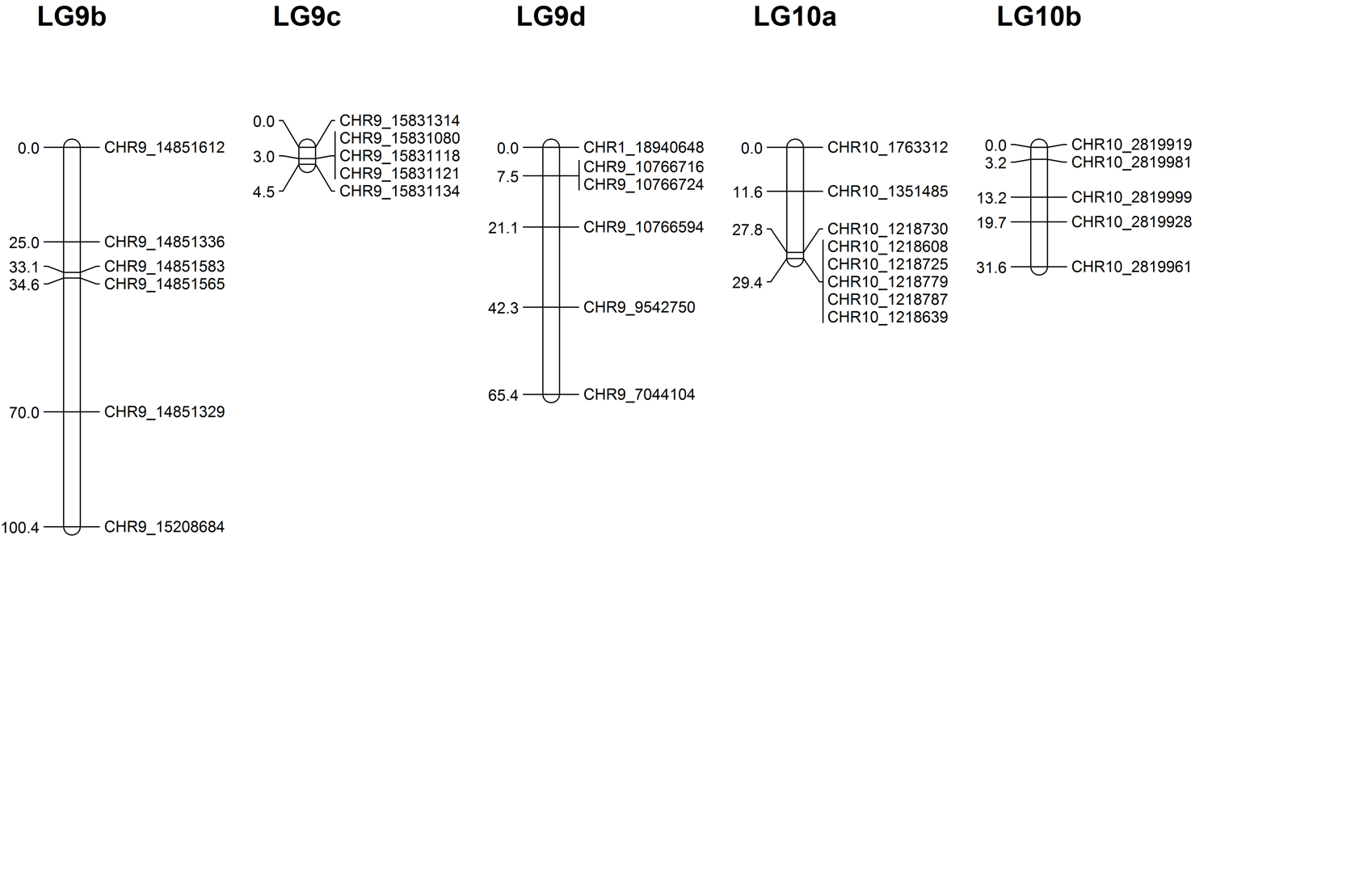
**

**
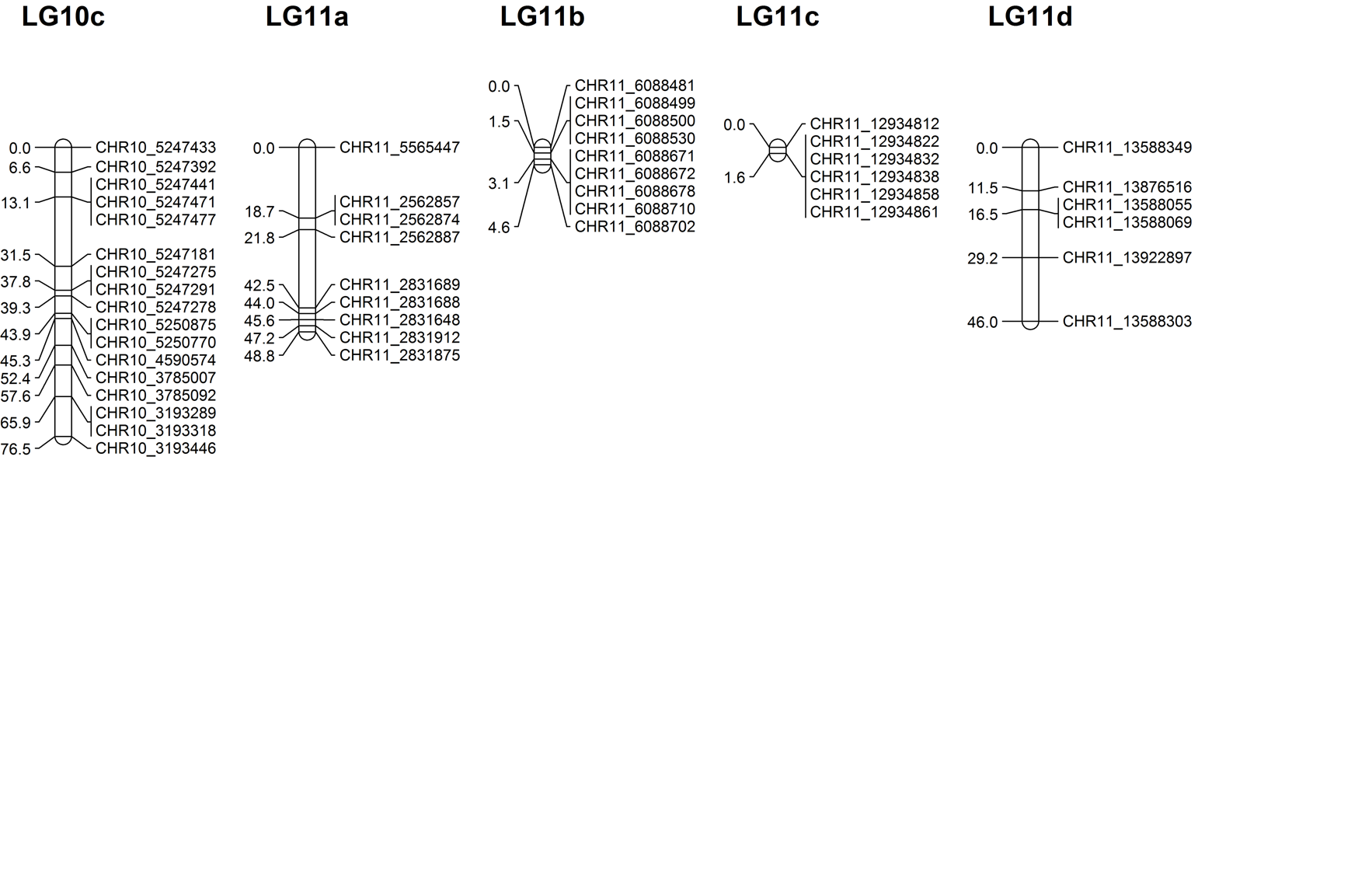
**

**Supplementary data 7.** Linkage maps (Maternal) continued**
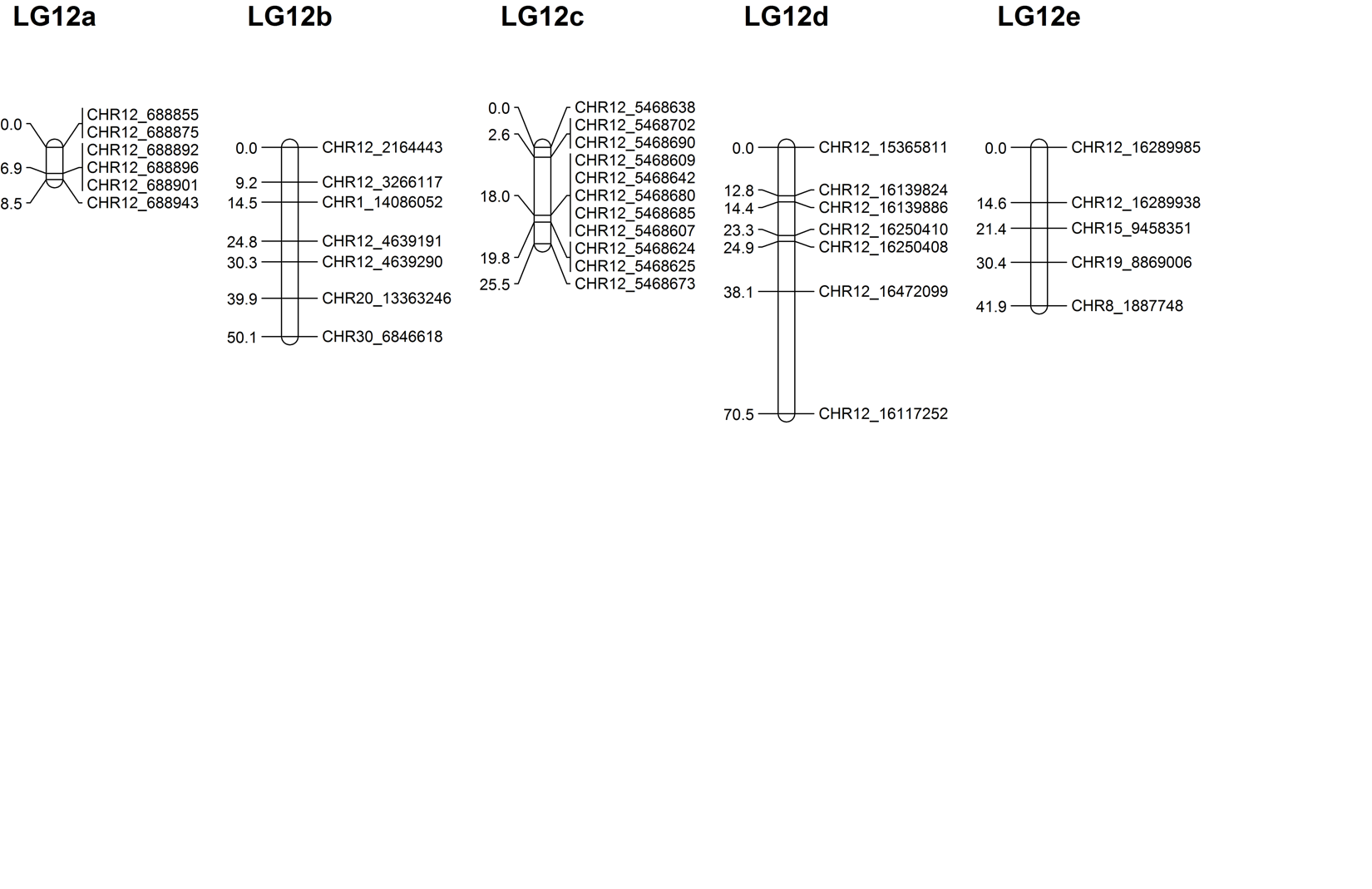

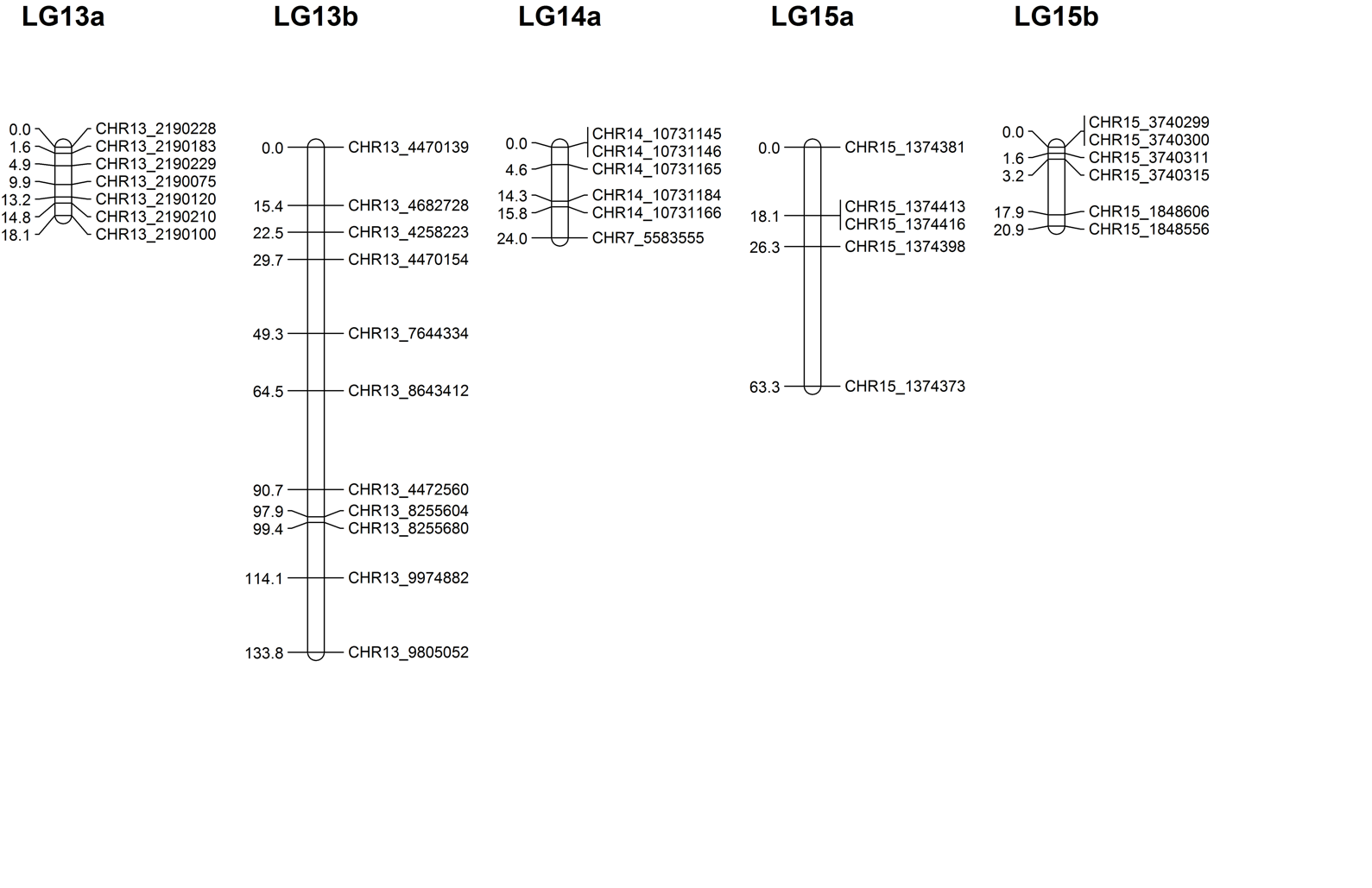
**

**Supplementary data 7.** Linkage maps (Maternal) continued

**
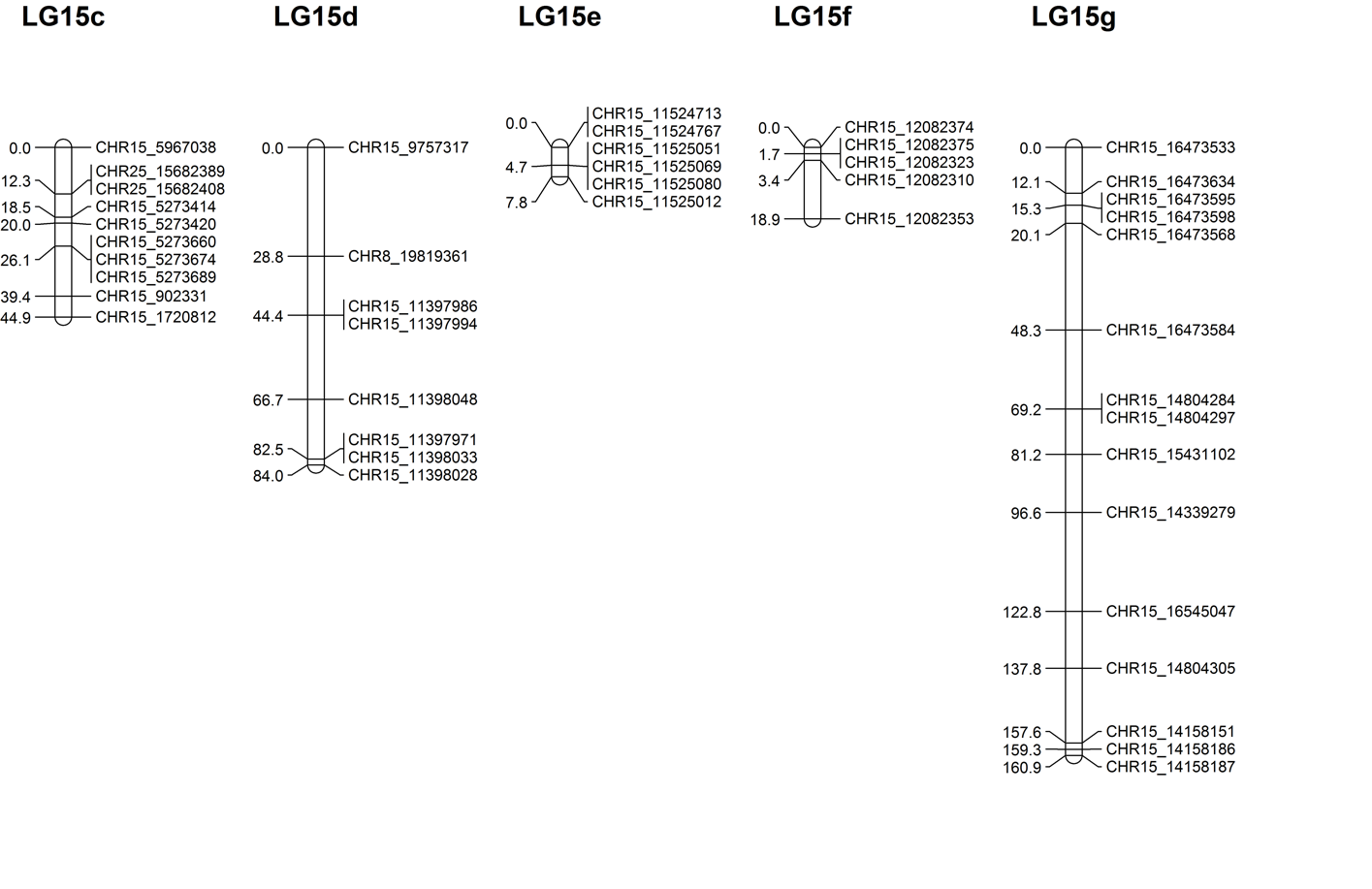
**

**Supplementary data 7.** Linkage maps (Maternal) continued

**
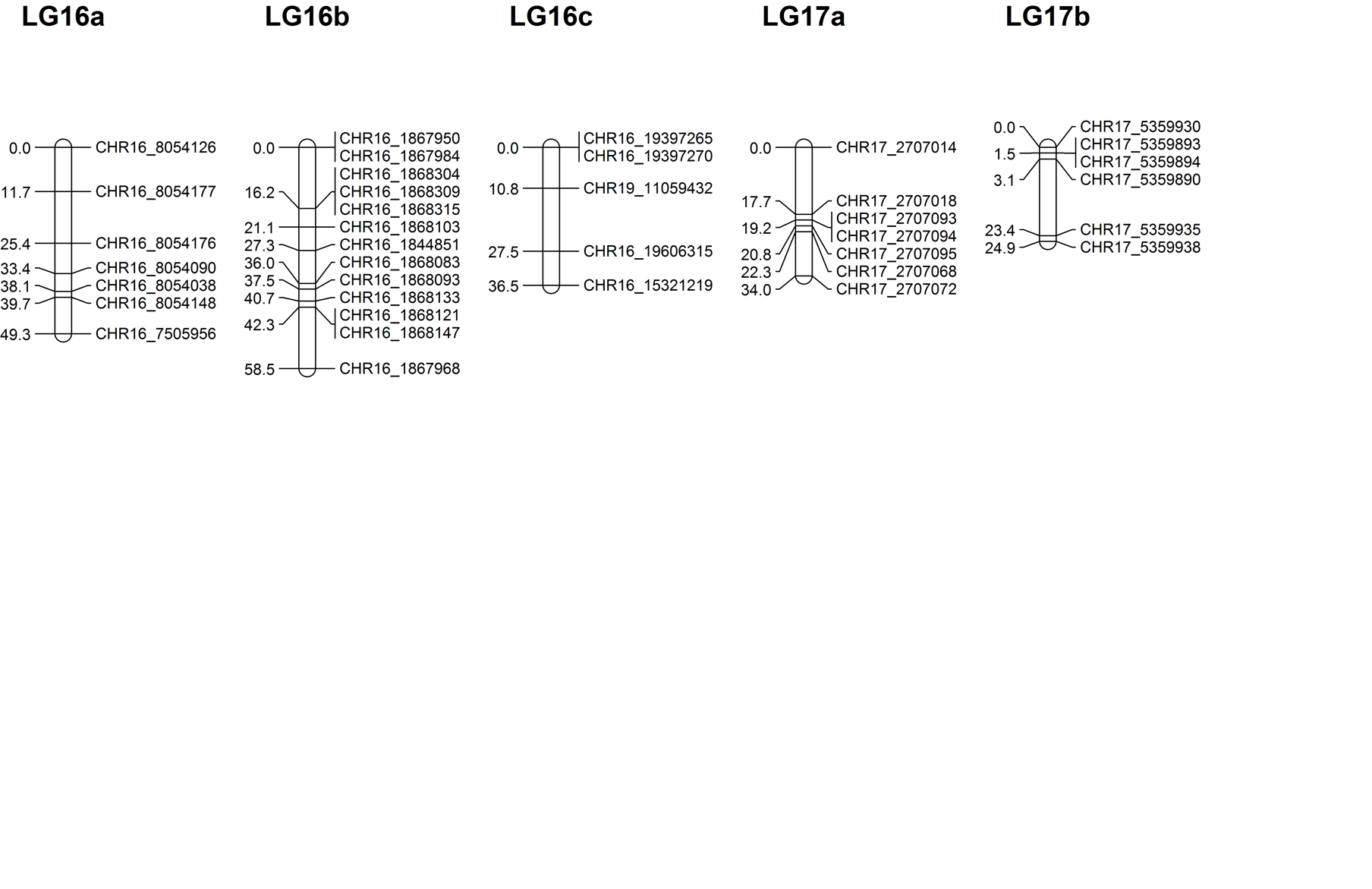
**

**
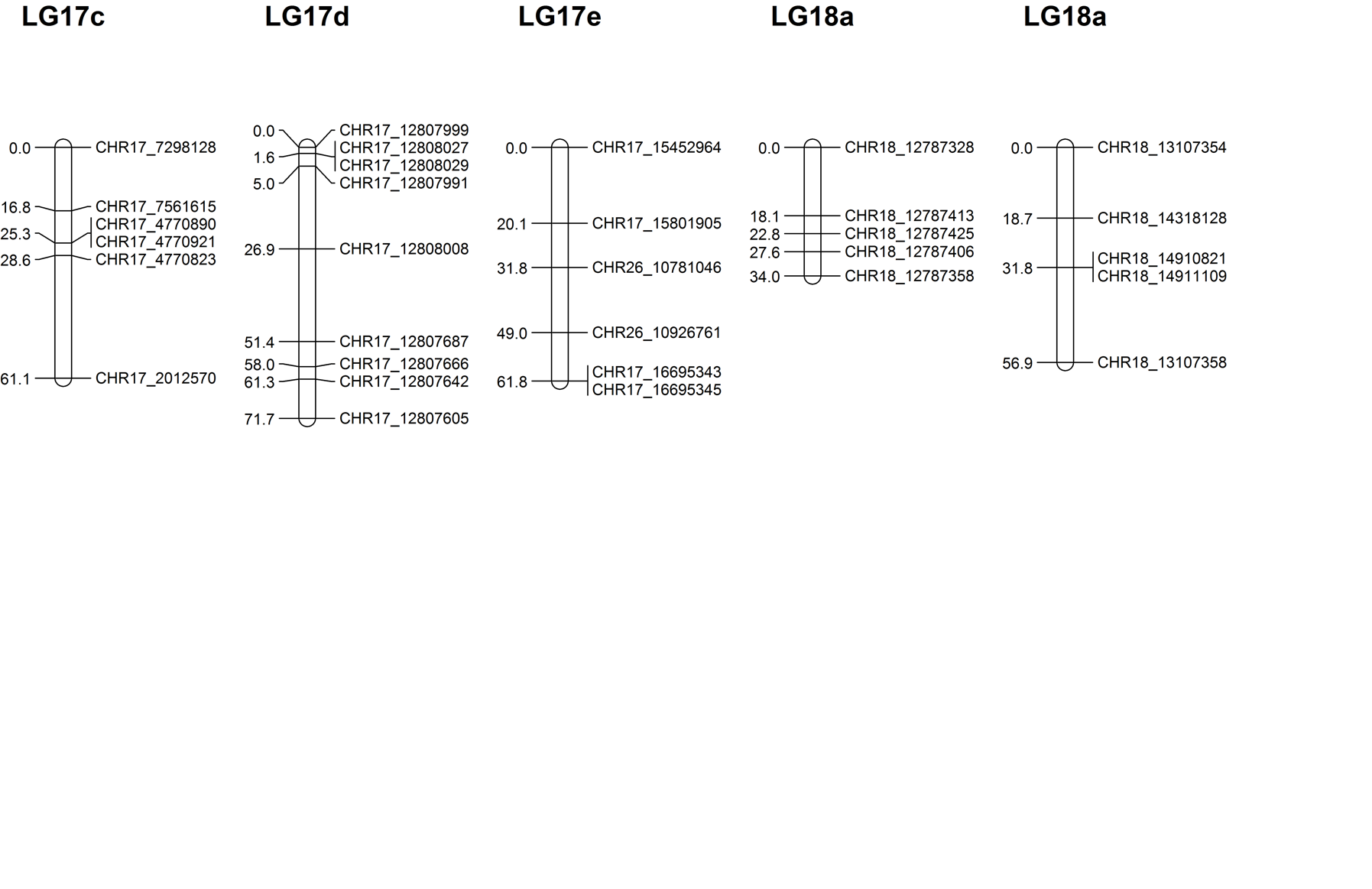
**

**Supplementary data 7.** Linkage maps (Maternal) continued**
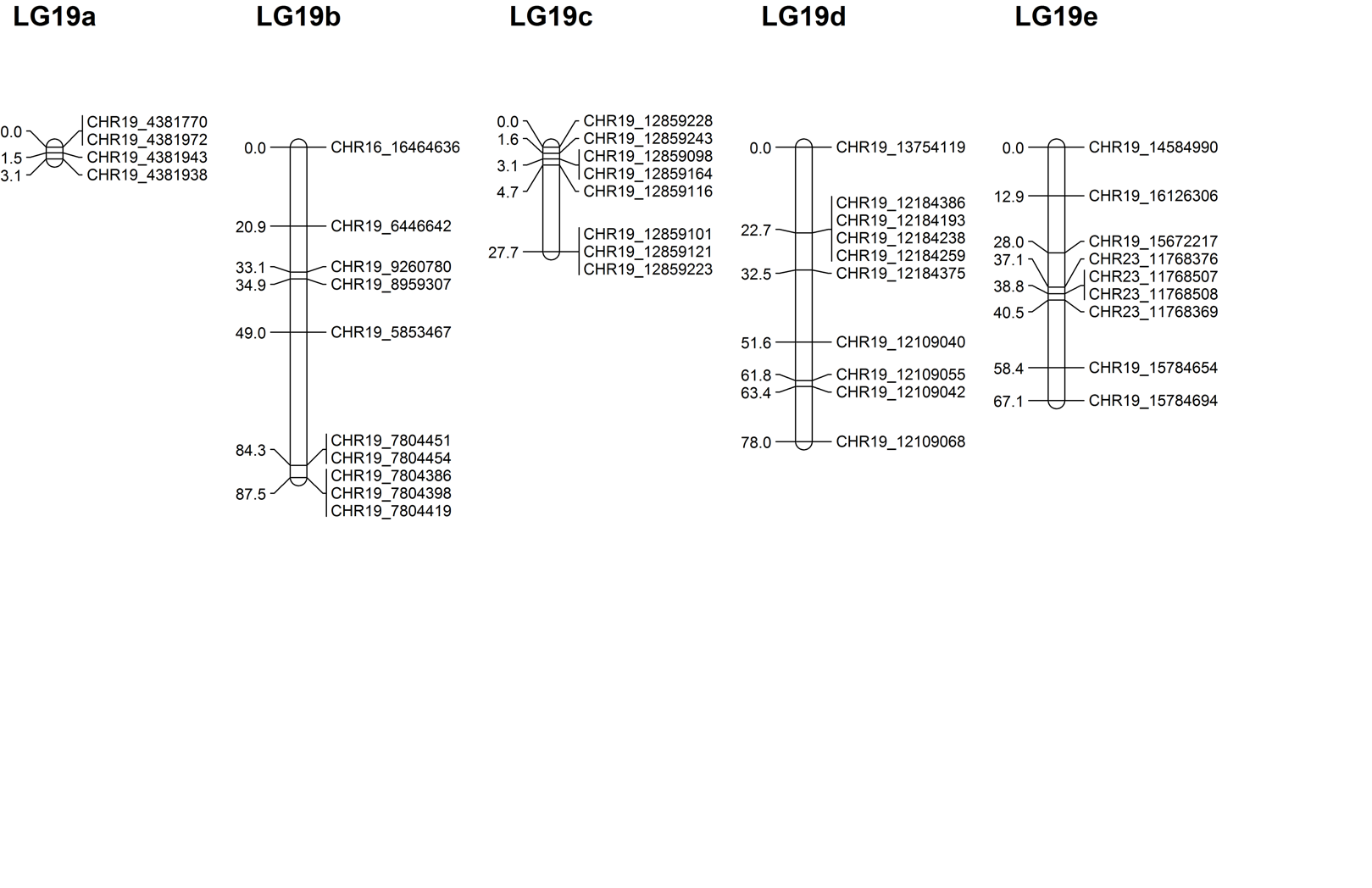

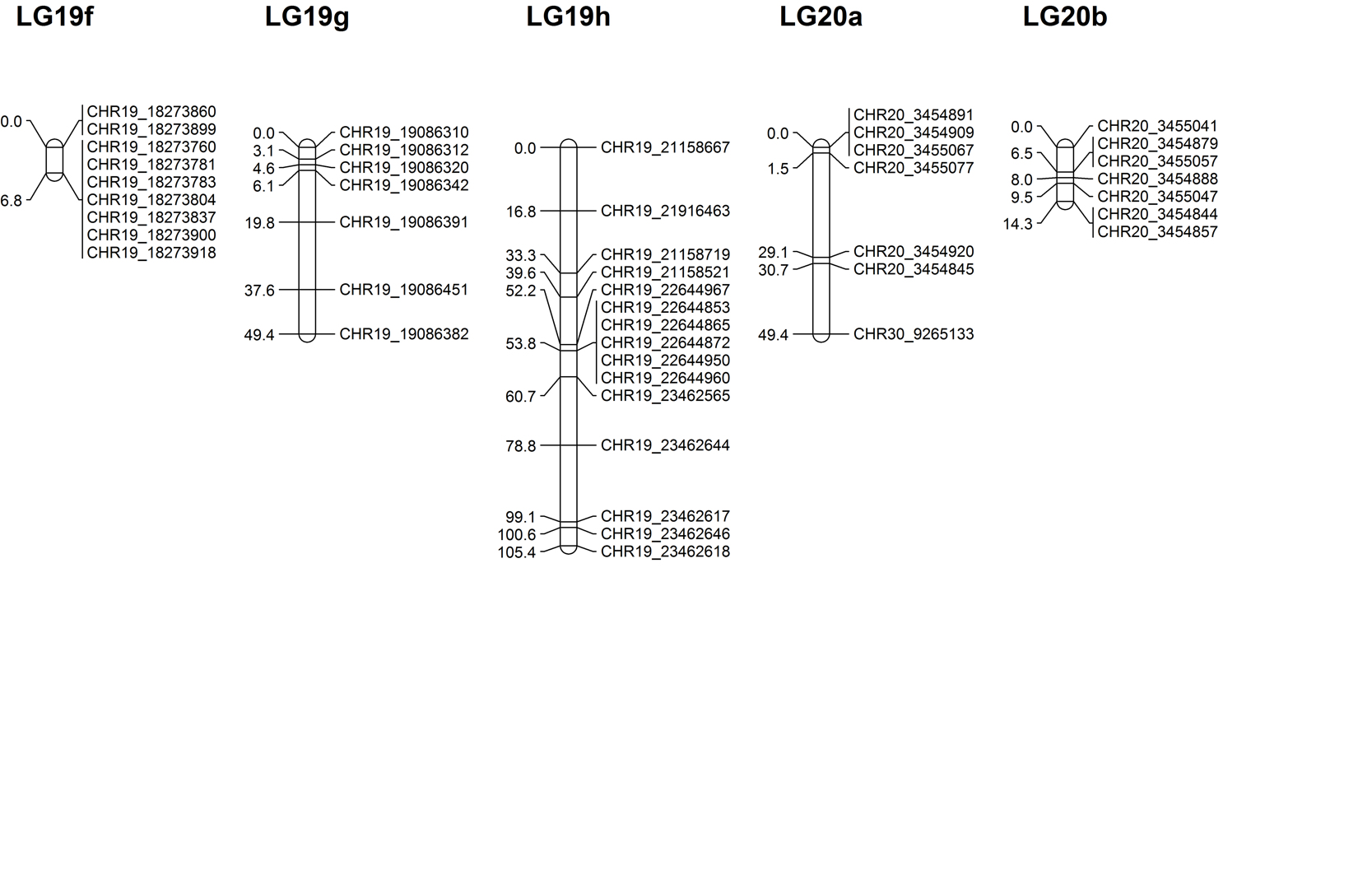
**

**Supplementary data 7.** Linkage maps (Maternal) continued

**
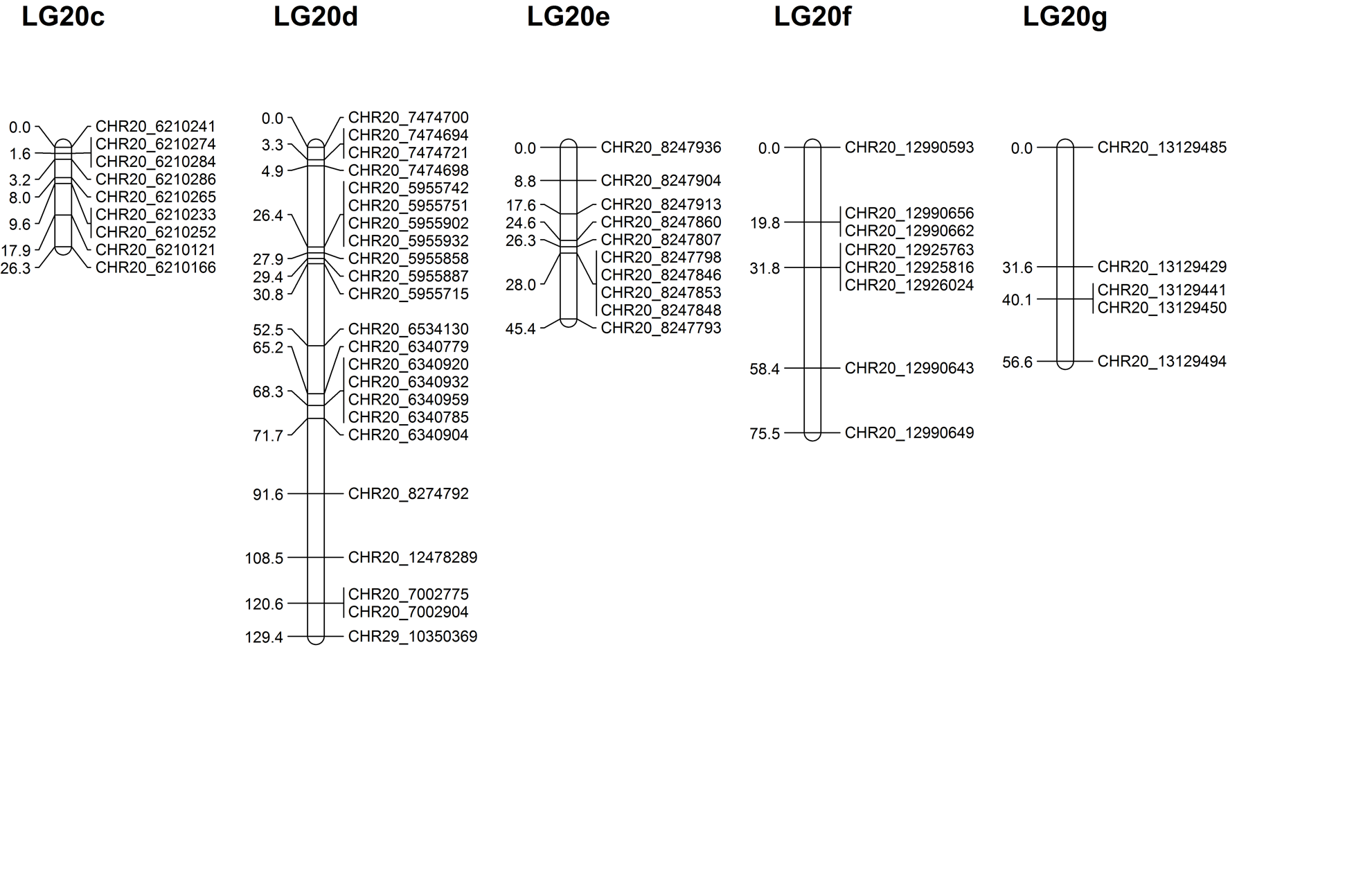
**

**Supplementary data 7.** Linkage maps (Maternal) continued**
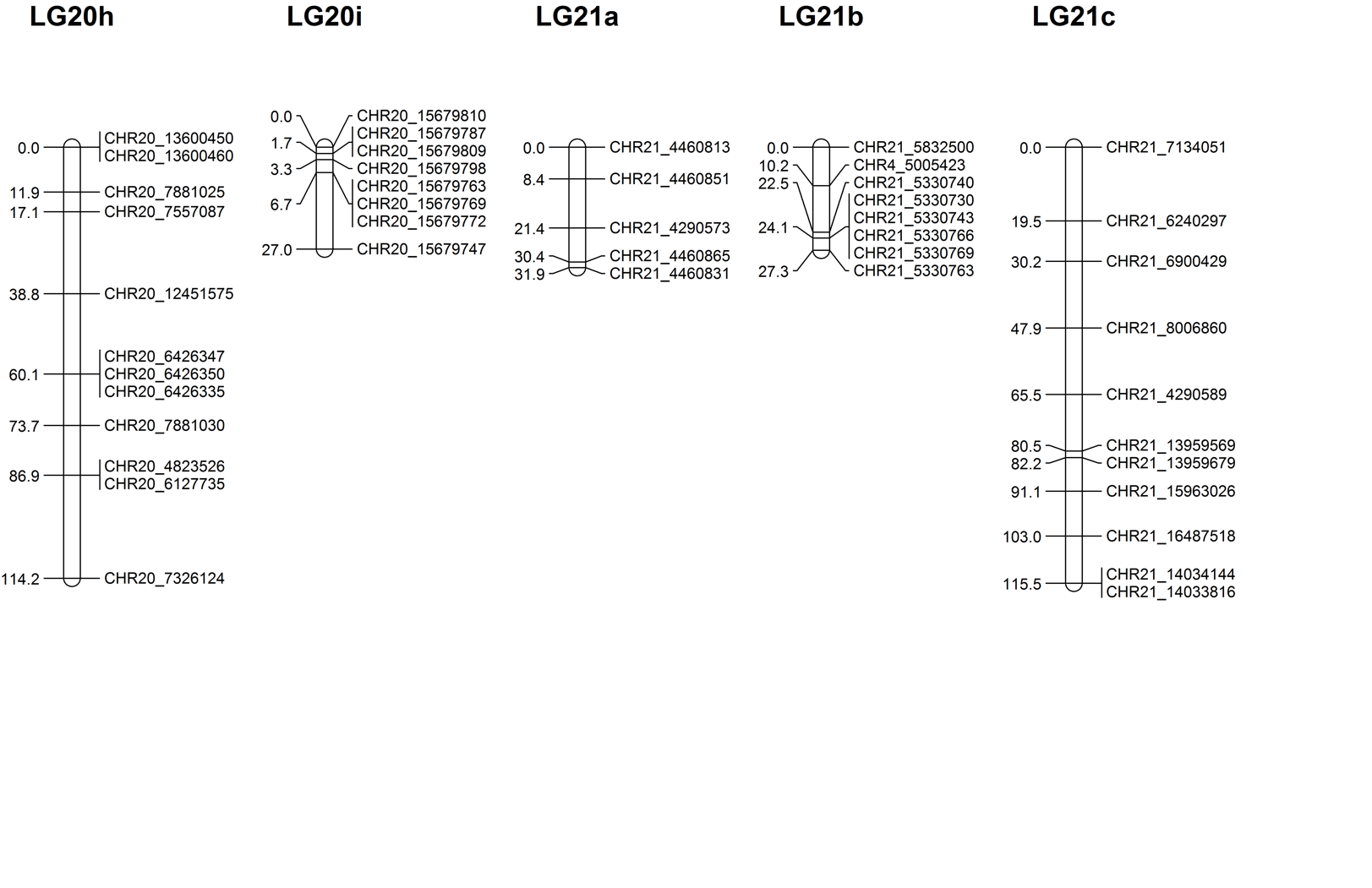
**

**Supplementary data 7.** Linkage maps (Maternal) continued

**
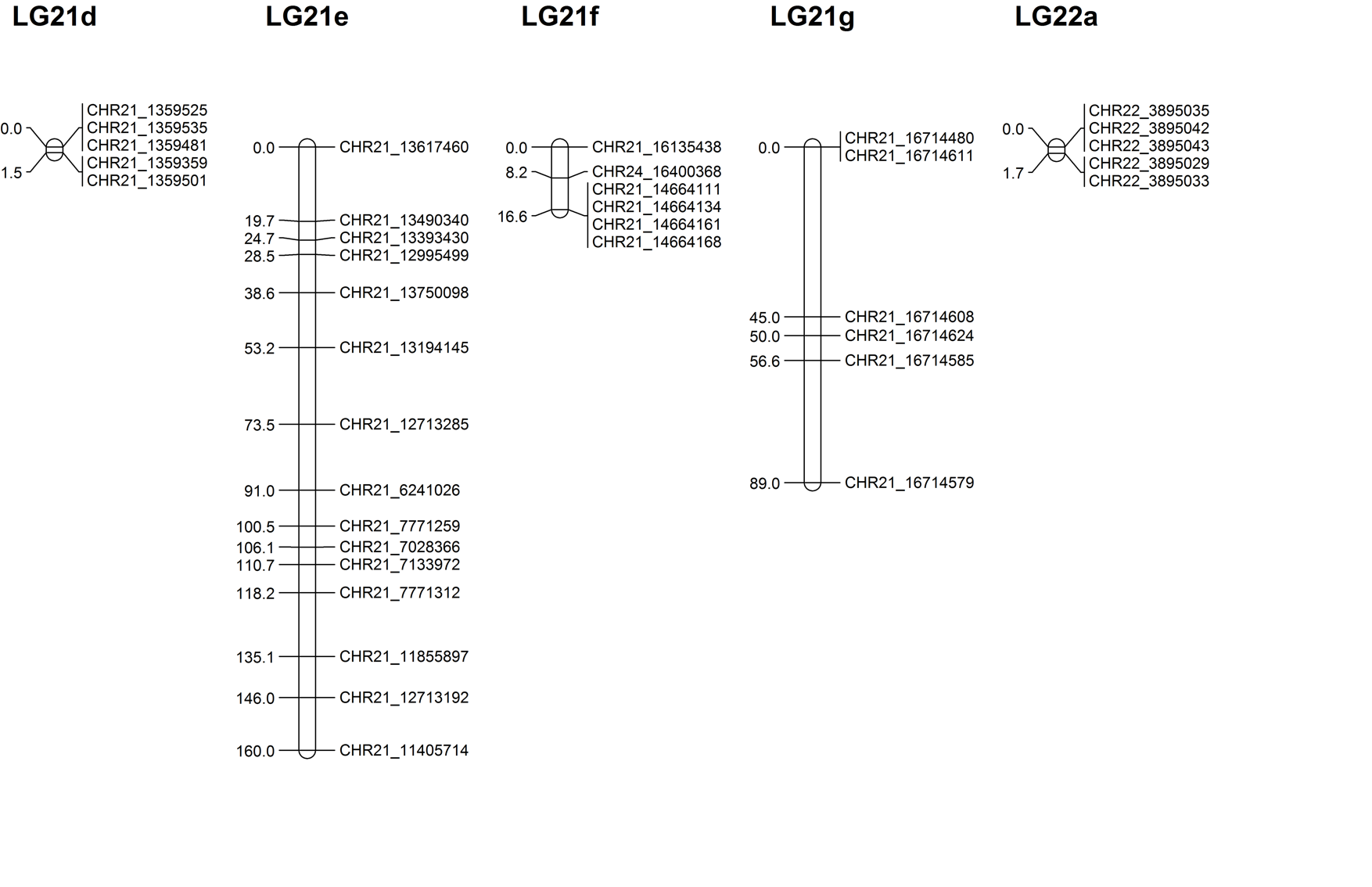
**

**Supplementary data 7.** Linkage maps (Maternal) continued

**
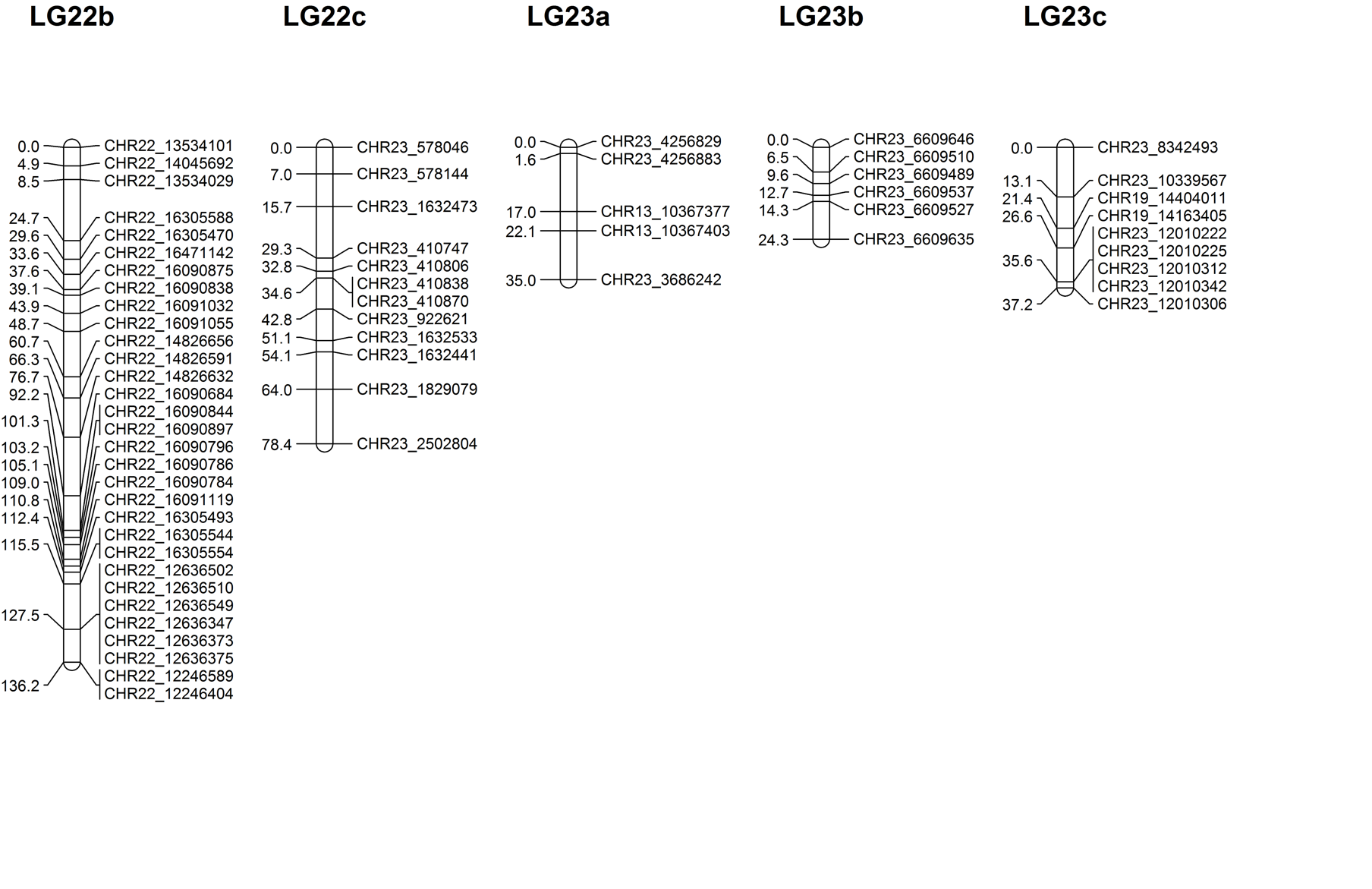
**

**Supplementary data 7.** Linkage maps (Maternal) continued

**
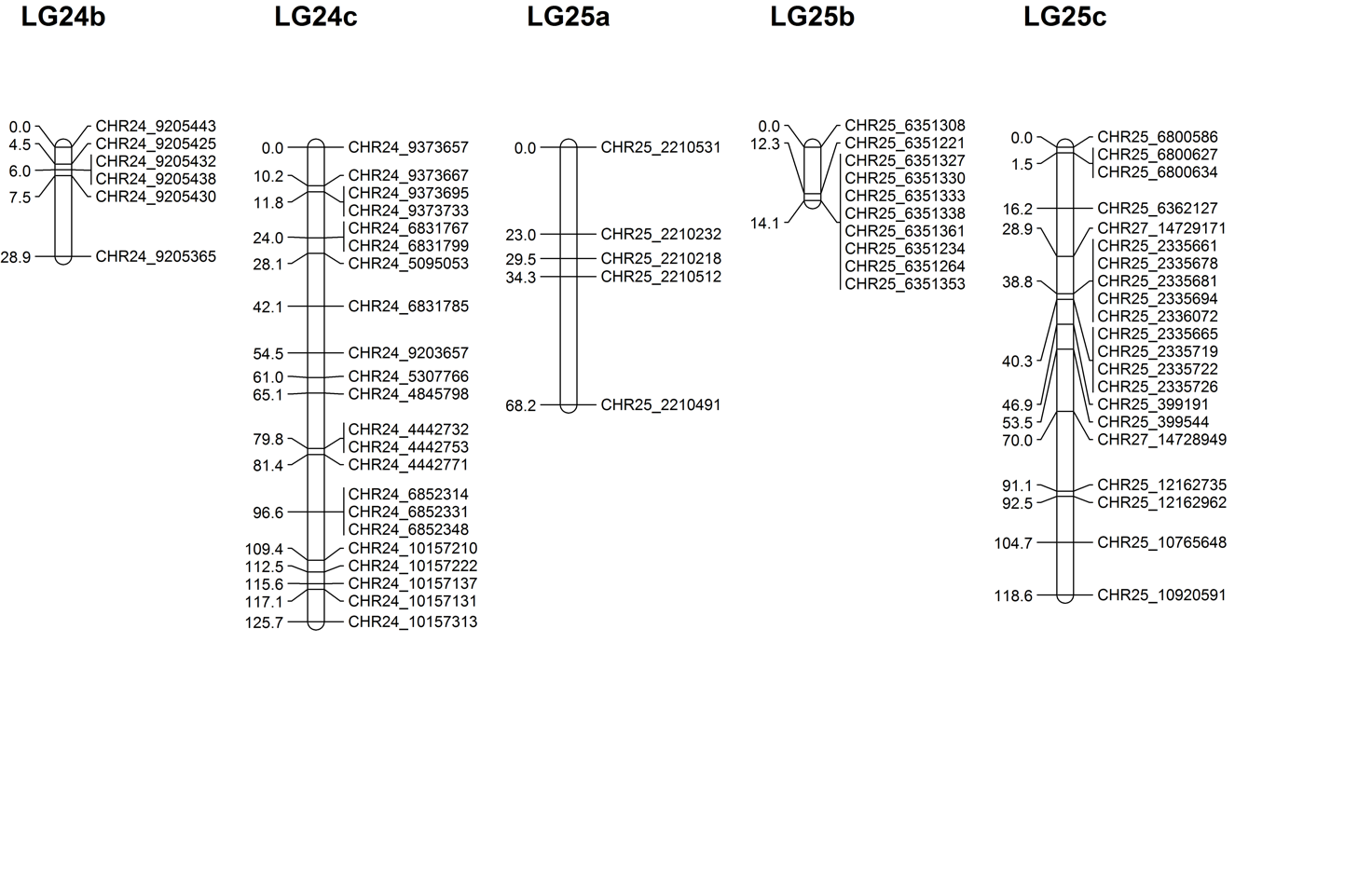
**

**
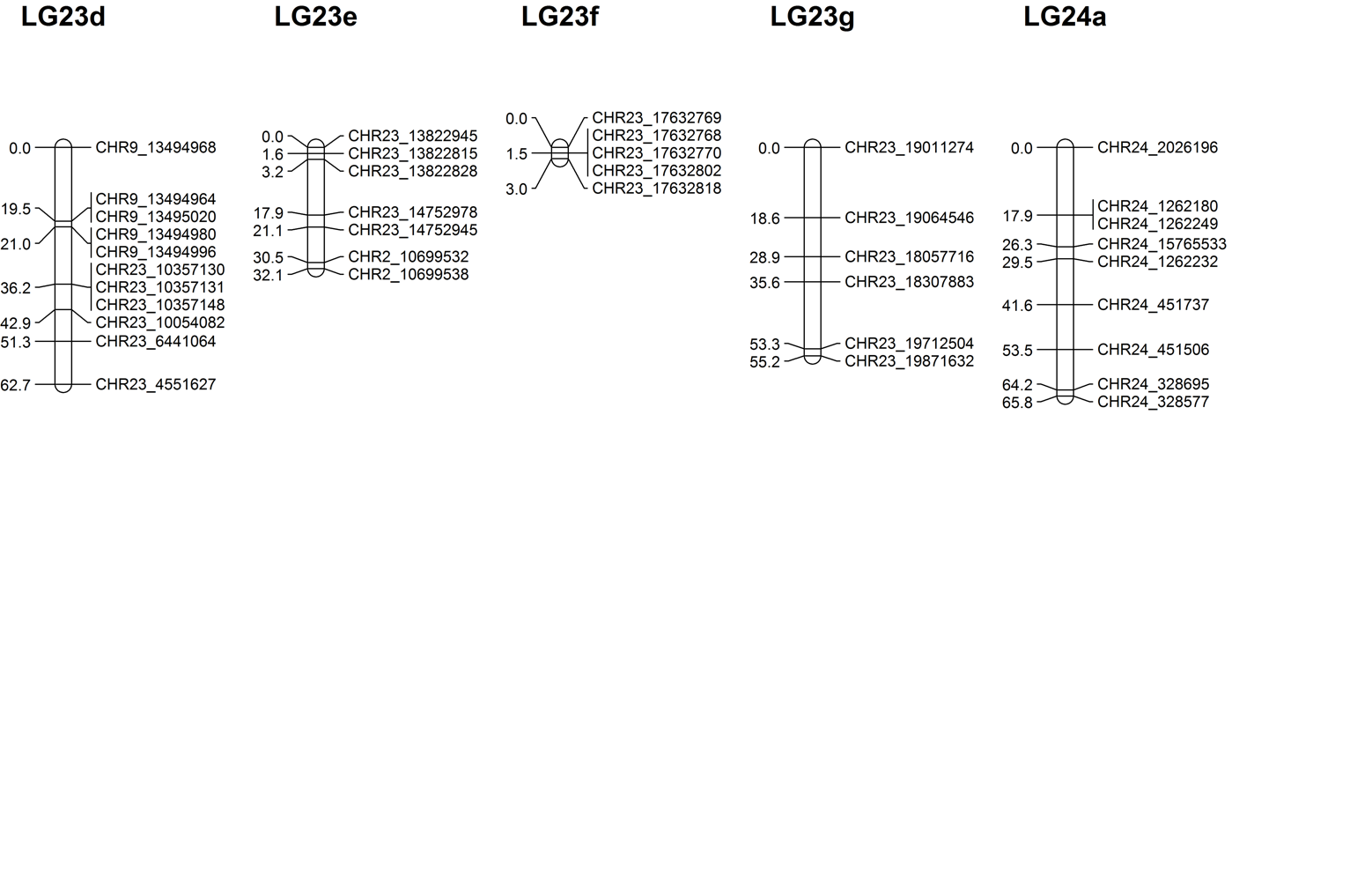
**

**Supplementary data 7.** Linkage maps (Maternal) continued**
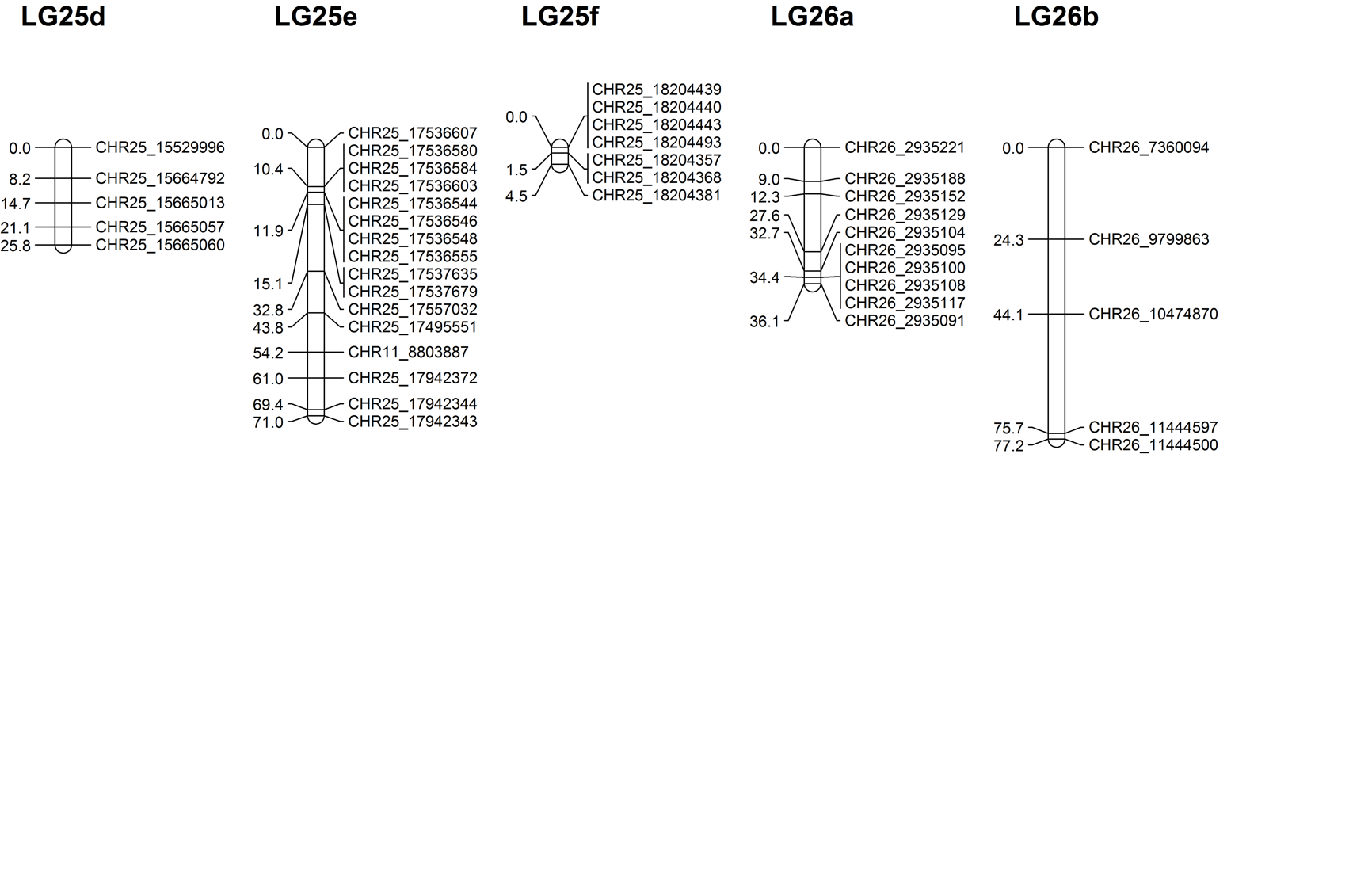

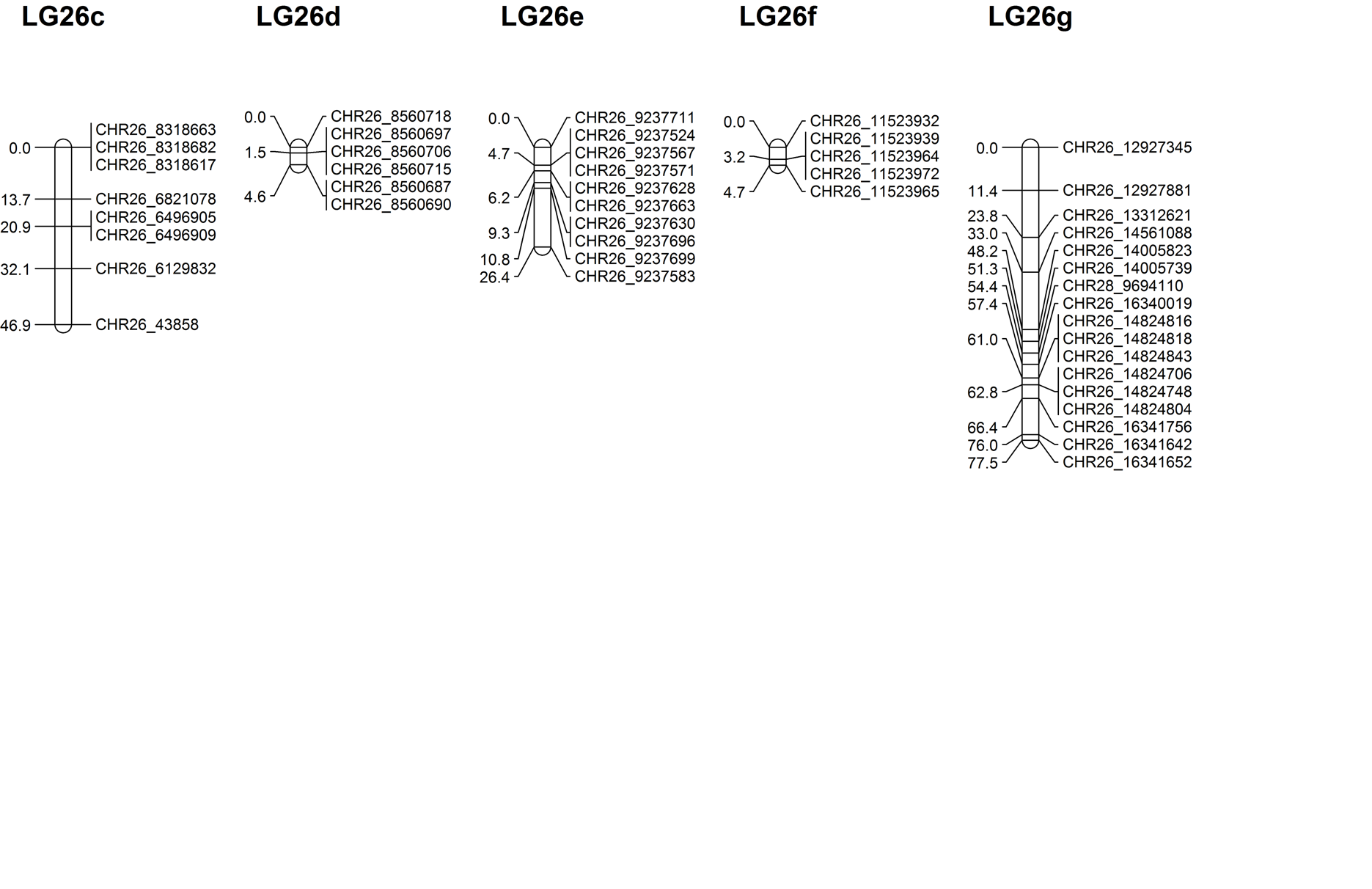
**

**Supplementary data 7.** Linkage maps (Maternal) continued**
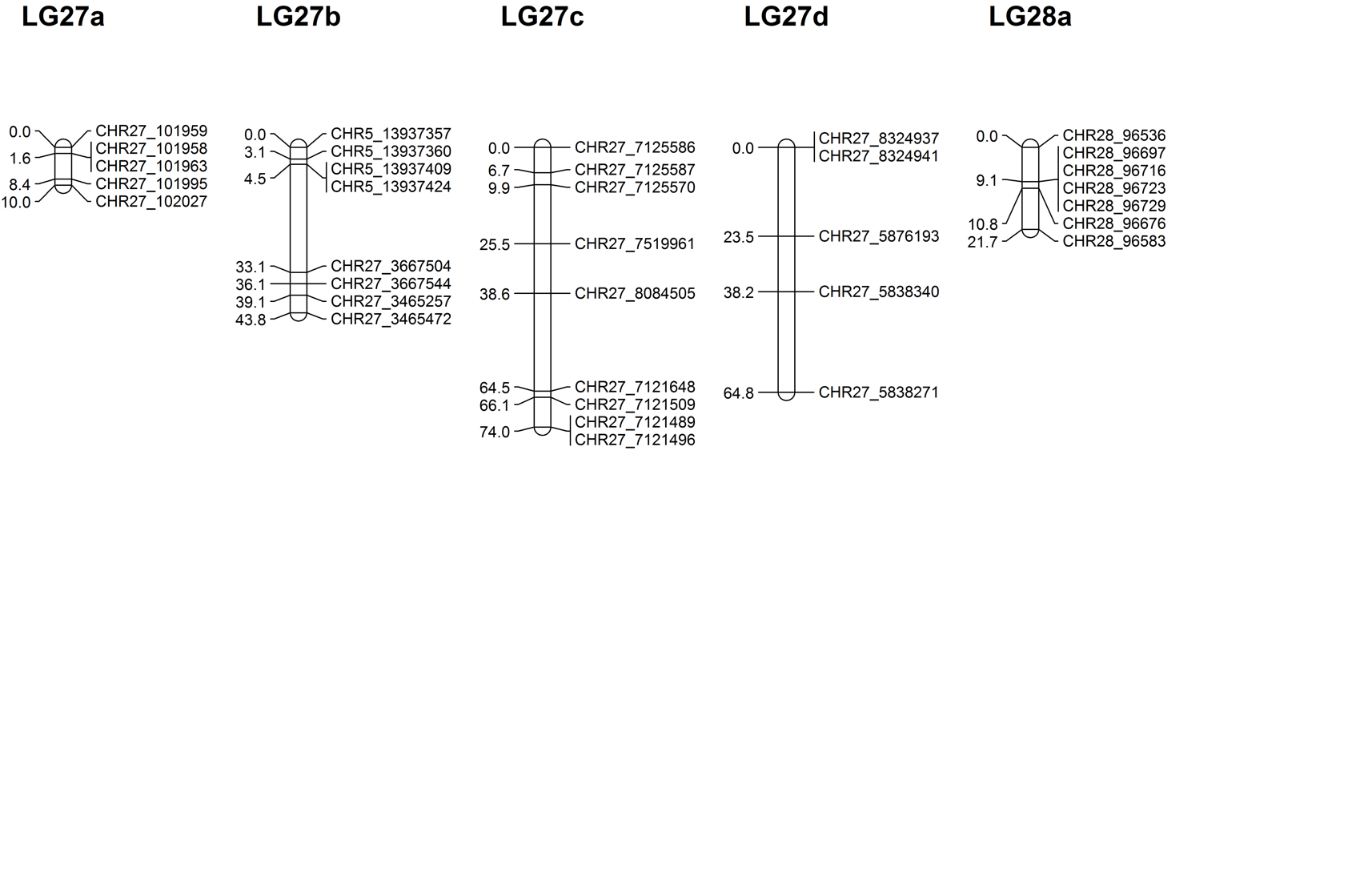
**

**Supplementary data 7.** Linkage maps (Maternal) continued

**
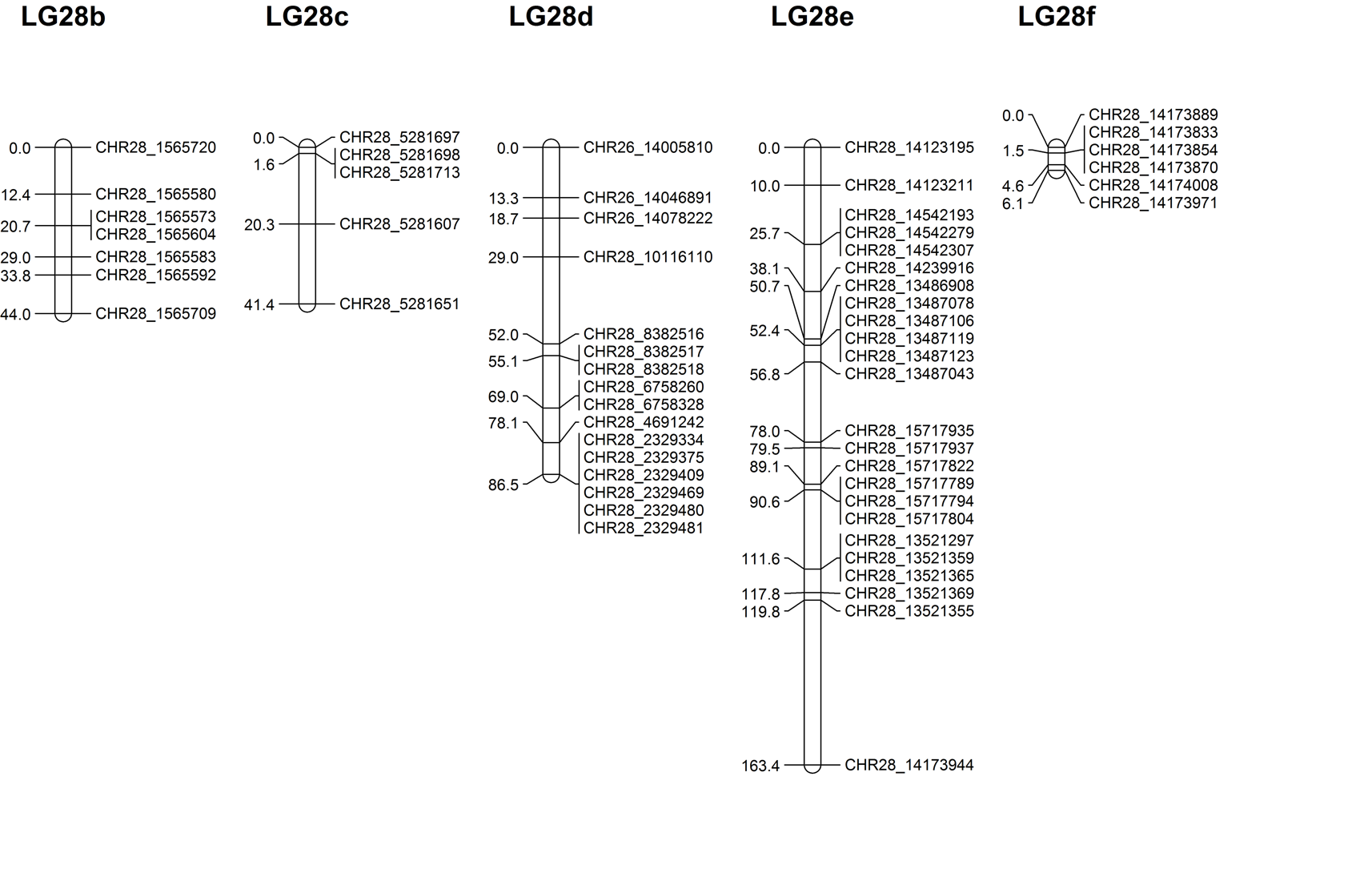
**

**Supplementary data 7.** Linkage maps (Maternal) continued**
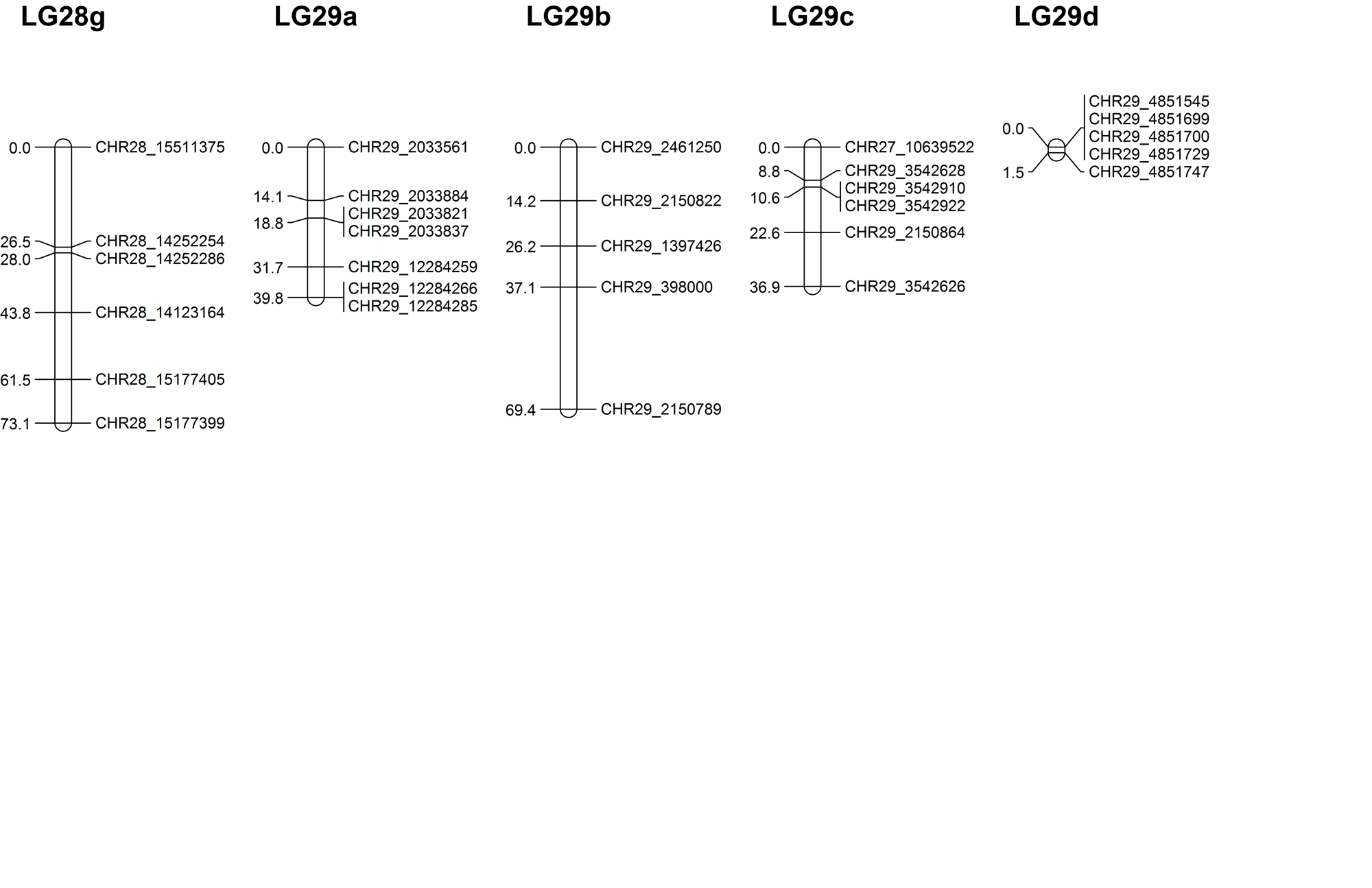
**

**
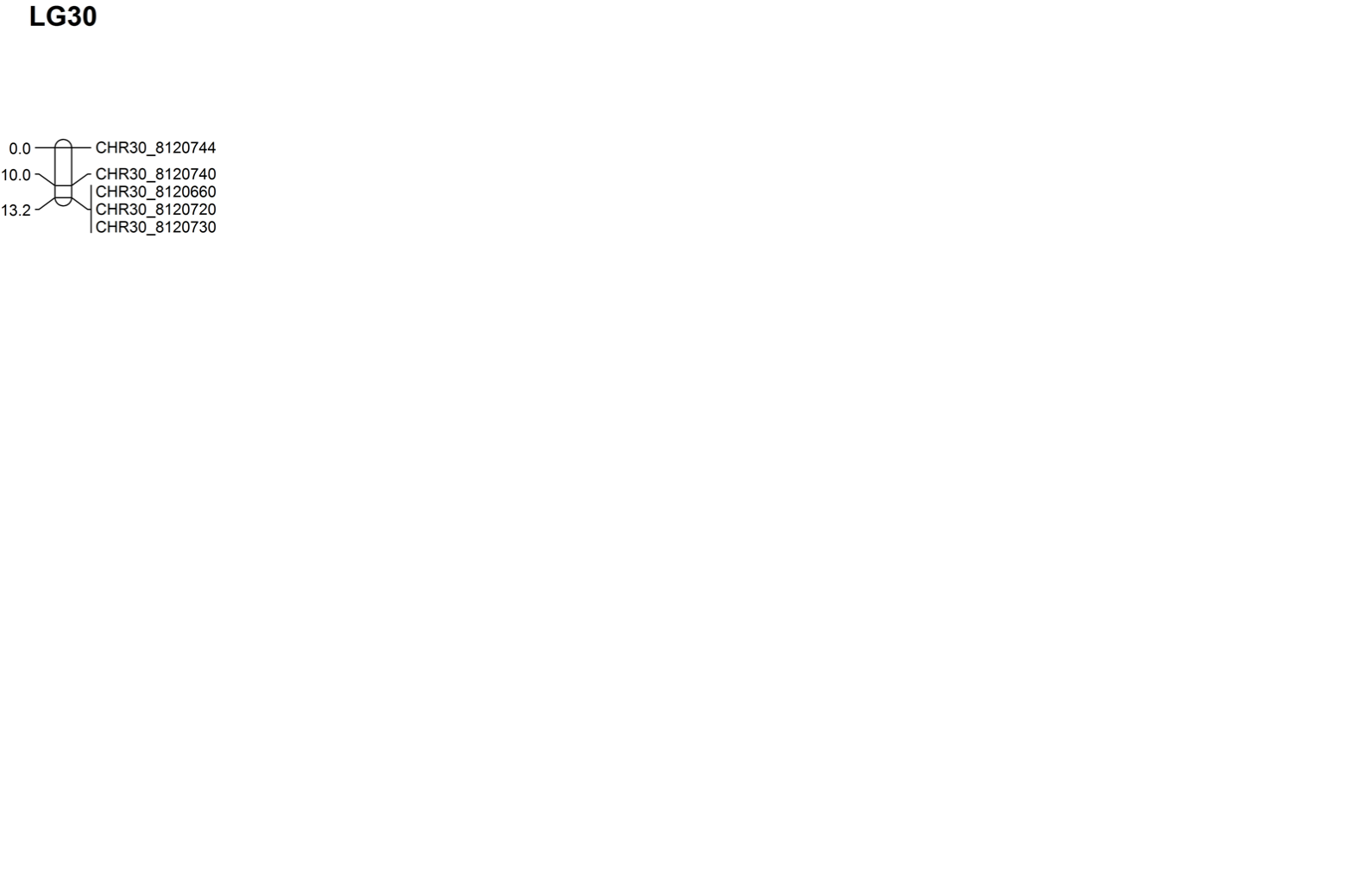
Supplementary data 7.** Linkage maps (Maternal) continued

**
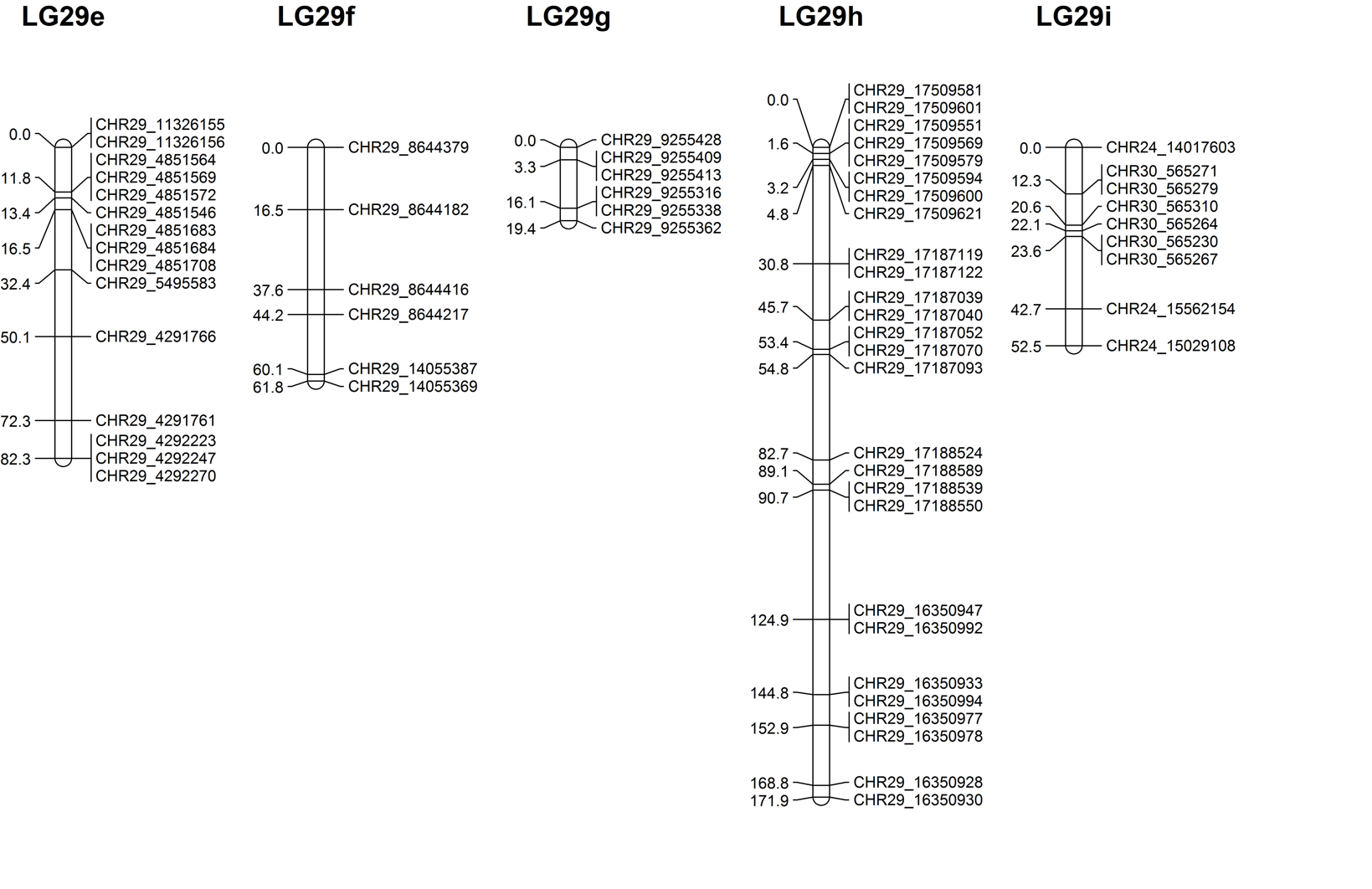
**

## Acknowledgements

The authors thank kiwifruit breeder Dr. Ron Beatson (PFR) that created the MECK crosses used in this study who works as a breeder for Plant and Food Research, and was responsible for the crosses that created the populations discussed in this paper.

# 
