# Supplementary material for: Peridermal fruit skin formation in Actinidia sp. (kiwifruit) is associated with genetic loci controlling russeting and cuticle formation
Source: BMC Plant Biol. 2021 Jul 14;21:334. doi: 10.1186/s12870-021-03025-2 (PMC8278711; doi:10.1186/s12870-021-03025-2)
Supplement: Supplementary file 2 — Additional file 2. MacNee et al. Mapping paper Supplementary Table 1. Cross referencing mapped markers to nearby genes matching genes in the literature already associated with pericarp/suberin/cuticle development [file 12870_2021_3025_MOESM2_ESM.docx]

**Supplementary table 1:** Candidate genes within QTL regions determined by literature analysis. Shading is for reader convenience and denotes blocks of genes grouped to their respective mapped QTL. Note: only genes within 2 Mbp of the marker location are listed.

| **Citation of bait origin** | **BLASTP Bait and provenance** | **Function** | **Kiwifruit genome assembly PS1.1.69.0 Hit** | **Expect** | **Distance from nearest mapped QTL (Mbp)** | **Mapped QTL and location** | **Reverse BLASTP best Arabidopsis match** |
| --- | --- | --- | --- | --- | --- | --- | --- |
| [30, 52-54] | GPAT5 (Arabidopsis); AT3G11430 | Biosynthesis of suberin polyester | Acc03744.1 | 0 | -1.17 | Mother:Periderm; CHR3: 13.50 Mbp | AT3G11430.1; GPAT5 |
| [41] | ESB1 (Arabidopsis); AT2G28670 | Unknown function but involved in Casparian strip formation, mutants have increased suberin | Acc03763.1 | 2.00E-55 | -0.84 | Mother:Periderm; CHR3: 13.50 Mbp | AT2G39430.1; Disease resistance-responsive (dirigent-like protein) |
| [30, 52-54] | CYP86B1/RALPH (Arabidopsis); AT5G23190 | VLCFA hydroxylase specifically involved in polyester monomer biosynthesis | Acc03882.1 | e-133 | 1.16 | Mother:Periderm; CHR3: 13.50 Mbp | AT3G56630.1; CYP94D2 |
| [12] | KCS11 (Arabidopsis); AT2G26640 | Biosynthesis of VLCFA | Acc04224.1 | 0 | -0.31 | Mother:Cuticle coverage/Mean periderm depth; CHR3: 19.90 Mbp | AT2G26640.1; KCS11 |
| [41] | WOX5 (Arabidopsis); AT3G11260 | Wuschel homeodomain transcription factor required for quiescent center (QC) function | Acc12050.1 | 1E-26 | -1.34 | Mother:Mean periderm depth; CHR11: 2.56 Mbp | AT1G46480.1; WOX4 |
| [41] ᶧ | UBP15 (Arabidopsis); AT1G17110 | Together with CUC2/CUC3-DA1 part of a regulatory module controlling initiation of axillary meristems. | Acc12149.1 | e-120 | 0.17 | Mother:Mean periderm depth/Cuticle thickness; CHR11: 2.56 Mbp | AT4G24560.1; UBP16 |
| [12] | MDP0000938736 (apple); AT2G26640 | Biosynthesis of VLCFA. | Acc12224.1 | 1.00E-118 | 1.36 | Mother:Mean periderm depth/Cuticle thickness; CHR11: 2.56 Mbp | AT1G19440.1; KCS4 |
| [41] ᶧ | ATCFL1 ASSOCIATED PROTEIN 1/FLOWERING BHLH 3 (Arabidopsis); AT1G51140 | bHLH transcription factor that interacts with CFL1 and along with CFLAP2 negatively regulates cuticle development. | Acc17096.1 | 1.00E-62 | 0.06 | Father:Periderm; CHR15: 10.39 Mbp | AT2G42280.1; ABA-RESPONSIVE KINASE SUBSTRATE 3 |
| [41] ᶧ | LORD OF THE RINGS 1 (Arabidopsis); AT5G50150 | Unknown function but mutants show defects in Casparian strip formation. | Acc17138.1 | e-152 | 0.52 | Father:Periderm; CHR15: 10.39 Mbp | AT5G56530.2; tRNA-splicing ligase (DUF239) |
| [12] | MDP0000069348 (apple); AT1G02205 | Likely aldehyde decarbonylase involved in wax synthesis. | Acc17172.1 | 0 | 1.04 | Father:Periderm; CHR15: 10.39 Mbp | AT1G02205.2; CER1 |
| [41] ᶧ | LACS2 (Arabidopsis); At1g49430 | Long chain acyl-CoA synthetase catalyzing synthesis of omega-hydroxy fatty acyl-CoA intermediates in the pathway to cutin synthesis. | Acc17262.1 | e-154 | -0.85 | Father:Periderm; CHR15: 13.13 Mbp | AT3G05970.1; LACS6 |
| [41] ᶧ | SCRAMBLED (Arabidopsis); AT1G11130 | Regulates cell shape and cell division planes in the L2 layer of floral meristems and the L1-derived outer integument of ovules. | Acc17248.1 | 2.00E-62 | -0.68 | Father:Periderm; CHR15: 13.13 Mbp | AT1G54820.1; Protein kinase superfamily protein |
| [41] ᶧ | ANAC073/SND2 (Arabidopsis); AT4G28500 | NAC Domain transcription factor involved in secondary wall biosynthesis. | Acc17306.1 | e-119 | 0.18 | Father:Periderm; CHR15: 13.13 Mbp | AT1G28470.1; ANAC010 |
| [41] ᶧ | NAM/ANAC018 (Arabidopsis); AT1G52880 | NAM which is required for the development of the shoot. | Acc17357.1 | 2.00E-85 | 0.71 | Father:Periderm; CHR15: 13.13 Mbp | AT3G15510.1; ANAC056 |
| [41] ᶧ | SCRAMBLED (Arabidopsis); AT1G11130 | Regulates cell shape and cell division planes in the L2 layer of floral meristems and the L1-derived outer integument of ovules. | Acc17365.1 | e-123 | 0.80 | Father:Periderm; CHR15: 13.13 Mbp | AT4G03390.1; STRUBBELIG-receptor family 3 |
| [41] ᶧ | AtMYB102 (Arabidopsis); AT4G21440 | Involved in cell differentiation. | Acc17393.1 | 2.00E-98 | 1.14 | Father:Periderm; CHR15: 13.13 Mbp | AT4G21440.1; AtMYB102 |
| [41] ᶧ | AtMYB42 (Arabidopsis); AT4G12350 | Regulation of secondary cell wall biogenesis. | Acc19921.1 | 1E-92 | -1.72 | Father:Semi russet; CHR18: 4.80 Mbp | AT4G22680.1; AtMYB85: regulation of secondary wall biogenesis |
| [41] ᶧ | AtMYB67 (Arabidopsis); AT3G12720 | Cell differentiation. | Acc20042.1 | 7E-61 | 0.34 | Father:Semi russet; CHR18: 4.80 Mbp | AT1G63910.1; AtMYB103: regulation of secondary wall biogenesis |
| [41] ᶧ | BDG/CED1 (Arabidopsis); At1g64670 | Extracellular protein that likely functions as an alpha-beta hydrolase and is required for normal cuticle formation. | Acc20103.1 | 0 | 1.89 | Father:Semi russet; CHR18: 4.80 Mbp | AT4G24140.1; alpha/beta-Hydrolases superfamily protein |
| [12] | MDP0000391122 (apple); MDP0000391122 | Acetyl-CoA-benzylalcohol acetyltransferase involved in cutin synthesis. | Acc33624.1 | 4.00E-58 | -1.76 | Mother:Russet/Periderm; CHR19: 12.10 Mbp | AT3G26040.1; HXXXD-type acyl-transferase family protein |
| [12] | MDP0000250127 (apple); AT5G46290 | Crucial for fatty acid synthesis. | Acc21320.1 | 0 | 0.30 | Mother:Russet/Periderm; CHR19: 12.10 Mbp | AT5G46290.1; KAS1 |
| [41] ᶧ | CYP77A6 (Arabidopsis); At3g10570 | Cytochrome p450 involved in cutin synthesis. | Acc21420.1 | e-103 | 0.97 | Mother:Russet; CHR19: 12.10Mbp | AT1G64950.1; CYP89A5, mRNA is cell-to-cell mobile |
| [41] ᶧ | AtMYB52 (Arabidopsis); AT1G17950 | Negative regulation of secondary cell wall biogenesis. | Acc23046.1; AcMYB56 | 2E-59 | -1.16 | Mother:Min depth ; CHR20: 12.99 Mbp | AT1G26780.2; AtMYB117, LATERAL ORGAN FUSION 1 |
| [41] ᶧ | BDG/CED1 (Arabidopsis); At1g64670 | Extracellular alpha-beta hydrolase required for normal cuticle formation. | Acc23049.1 | e-112 | -1.11 | Mother:Min depth ; CHR20: 12.99 Mbp | AT5G17780.1; alpha/beta-Hydrolases superfamily protein functioning in acyl-lipid metabolism |
| [41] ᶧ | AtMYB94 (Arabidopsis); AT3G47600 | Involved in cell differentiation. | Acc23052.1 | 1E-80 | -1.06 | Mother:Min depth ; CHR20: 12.99 Mbp | AT1G08810.1; AtMYB60. Expressed exclusively in guard cells and required for light-induced opening of stomata |
| [41] ᶧ | CASP1 (Arabidopsis); AT2G36100 | Membrane bound protein involved in formation of the Casparian strip. Along with CASP 2 it is required for the localization of ESB1. | Acc26219.1 | 2E-39 | -1.04 | Mother:Russet; CHR23: 11.76 Mbp | AT5G06200.1; CASP4 |
| [41] ᶧ | FAR4 (Arabidopsis); At3g44540 | One of the alcohol-forming fatty acyl-CoA reductase family which generates primary fatty alcohols associated with suberin deposition. | Acc26234.1 | e-155 | -0.86 | Mother:Russet; CHR23: 11.76 Mbp | AT4G33790.1; FAR3 |
| [11] | ABCG32/PEC1/ATPDR4/PDR4 (Arabidopsis); At2g26910 | ATP binding cassette transporters. Required for the formation of a functional cuticle. | Acc26238.1 | 0 | -0.81 | Mother:Russet; CHR23: 11.76 Mbp | AT2G36380.1; ABCG34 |
| [45] | (Populus trichocarpa); Potri.007G063300.1 | SHORT-ROOT-like gene. | Acc21589.1 | 7E-57 | 0.07 | Mother:Russet; CHR23: 11.76 Mbp | Potri.014G164400.1/AT5G48150.1; PAT1 phytochrome signalling/photomorphogenesis |
| [30] | ANAC78 (Arabidopsis); AT5G04410 | Regulates flavonoid synthesis. | Acc27590.1 | e-121 | 0.21 | Father:Semi russet; CHR24: 9.52 Mbp | AT5G04410.1; ANAC078 |
| [41] ᶧ | REVOLUTA (Arabidopsis); AT5G60690 | HD-Zip regulating lateral meristem initiation. Overlapping functions with PHAVOLUTA and PHABULOSA. mRNA cell-to-cell mobile. | Acc27611.1 | e-124 | 0.62 | Father:Semi russet; CHR24: 9.52 Mbp | AT5G60690.1; REVOLUTA, regulates meristem initiation at lateral positions |
| [41] ᶧ | ESB1 (Arabidopsis); AT2G28670 | Unknown function but involved in Casparian strip formation, localization dependent on CASP1 and 2. *esb1* mutants have increased levels of suberin. | Acc27679.1 | 3E-55 | -1.50 | Father:Mean periderm depth/Cuticle coverage/Perider; CHR24: 12.64 Mbp | AT3G55230.1; Disease resistance-responsive (dirigent-like protein) |
| [30, 52-54] | GPAT5 (Arabidopsis); AT3G11430 | Biosynthesis of suberin polyester. | Acc27732.1 | 0 | -0.86 | Father:Mean periderm depth/Cuticle coverage/Perider; CHR24: 12.64 Mbp | AT3G11430.1; GPAT5 |
| [41] ᶧ | WOX5 (Arabidopsis); AT3G11260 | Homeodomain transcription factor required for quiescent center (QC) function. | Acc27793.1 | 1E-49 | -0.16 | Father:Mean periderm depth/Cuticle coverage/Perider; CHR24: 12.64 Mbp | AT3G11260.1; WOX5 |
