# Supplementary material for: Peridermal fruit skin formation in Actinidia sp. (kiwifruit) is associated with genetic loci controlling russeting and cuticle formation
Source: BMC Plant Biol. 2021 Jul 14;21:334. doi: 10.1186/s12870-021-03025-2 (PMC8278711; doi:10.1186/s12870-021-03025-2)
Supplement: Supplementary file 3 — Additional file 3. CKMEXCK_PS1.1.69.0_K857. [file 12870_2021_3025_MOESM3_ESM.docx]

Compressed variant call file for GBS mapping [634.1 MB]; hosted at [zonodo.org](http://zonodo.org): [https://doi.org/10.5281/zenodo.4722054](http://dx.doi.org/10.5281/zenodo.4722054)
